# STAR: a simple TAL effector assembly reaction using isothermal assembly

**DOI:** 10.1038/srep33209

**Published:** 2016-09-12

**Authors:** Sabine Gogolok, Claudia Garcia-Diaz, Steven M. Pollard

**Affiliations:** 1 MRC Centre for Regenerative Medicine, University of Edinburgh, Edinburgh bioQuarter, 5 Little France Drive, Edinburgh EH16 4UU, UK

## Abstract

Transcription activator-like effectors (TALEs) contain modular programmable DNA binding domains. Fusing TALEs with effector domains creates synthetic transcription factors (TALE-TFs) or nucleases (TALENs), enabling precise gene manipulations. The construction of TALEs remains challenging due to their repetitive sequences. Here we report a simple TALE assembly reaction (STAR) that enables individual laboratories to generate multiple TALEs in a facile manner. STAR uses an isothermal assembly (‘Gibson assembly’) that is labour- and cost-effective, accessible, rapid and scalable. A small 68-part fragment library is employed, and the specific TALE repeat sequence is generated within ~8 hours. Sequence-verified TALENs or TALE-TF plasmids targeting 17 bp target sequences can be produced within three days, without the need for stepwise intermediate plasmid production. We demonstrate the utility of STAR through production of functional TALE-TFs capable of activating human *SOX2* expression. STAR addresses some of the shortcomings of existing Golden Gate or solid-phase assembly protocols and enables routine production of TALE-TFs that will complement emerging CRISPR/Cas9-based reagents across diverse applications in mammalian stem cell and synthetic biology.

The recent emergence of programmable DNA binding proteins, built upon transcription activator-like effector proteins (TALEs) and CRISPR/Cas9 architectures, has heralded a new era of simple and efficient genome and chromatin editing. These tools provide the foundations for new approaches to programming gene function and gene regulatory circuits across a multitude of model organisms and cell lines^1^.

TALEs were originally identified in the bacterial plant pathogen *Xanthomonas*, where they subvert gene expression of host cells^2^. The DNA binding domain of TALEs comprises an extended array of 34 amino acid repeats. Each repeat has one of four distinct repeat variable diresidues (RVDs)–differing only at amino acid positions 12 and 13– that confers nucleotide binding specificity^3^. This simple code enables straightforward customization of synthetic TALEs to bind pre-determined target sequences. TALEs can be fused to various effector domains such as: the endonuclease FokI, transcriptional regulatory domains (e.g. VP16 and KRAB)^4^, or histone modifiers (e.g. LSD1)^5^. This generates TALE nucleases (TALENs), TALE-transcription factors (TALE-TFs) or TALE-chromatin editors (TALE-CEs), respectively.

The RNA-guided clustered regularly interspaced short palindromic repeats (CRISPR)-associated protein 9 (Cas9) has emerged as an alternative to TALENs, with significant advantages^6^. The bi-partite nature of the CRISPR/Cas system has proven particularly useful for genome-wide screening, as large libraries of targeting guide RNAs (gRNAs) can be produced at little cost^6^,^7^. Despite great enthusiasm for CRISPR/Cas9, TALEs have certain inherent advantages–particularly when deployed as TALE-TFs and TALE-CEs. First, in contrast to Cas9, TALEs are directly tethered to the effector domains, which means it is possible to simultaneously deliver a multitude of distinct effectors across multiple loci. Second, TALE-TFs may display greater activity as transcriptional activators than comparable dCas9-TFs, due to their dynamic binding to target loci^6^,^7^,^8^. Third, unwanted unwinding of target enhancers occurs when dCas9-gRNA binds^8^. Further studies are needed to define the strengths and weaknesses of each technology, especially detailed head-to-head comparisons of performance of dCas9-TFs and TALE-TFs.

TALE expression plasmids are difficult to construct due to their repetitive nature. A popular protocol for TALE contruction is based on the Golden Gate (GG) cloning, i.e. stepwise cloning of TALEs using type II restriction enzymes^8^,^9^. The procedure typically requires three to five days and depends on production of intermediate vector products, which is time-consuming and cumbersome. Alternative scalable methods based on solid-phase assembly have been developed, such as FLASH and ICA^9^,^10^,^11^. These methods allow rapid and automated production of TALEs, but they also require significant expertise, equipment, and large plasmid libraries, especially when high-throughput production is desired. Ligation independent cloning (LIC)^10^,^11^,^12^, FairyTALE^13^ (a single reaction GG method), and reTALE^14^ provide alternative methods that address some of these shortcomings, but automated liquid handling is ideally required and the initial setup and investment is non-trivial.

There remains an unmet need for a user-friendly and rapid TALE assembly strategy requiring only a small and simple starting library. An ideal approach would involve simple setup, minimal hands-on time, have no requirement for intermediate plasmid assembly, and could be implemented by any individual laboratory, using standard tools of molecular biology to produce tens of TALENs or TALE-TFs. To address this need, we devised a simple TALE assembly reaction (STAR), using a novel strategy involving only a 68-part plasmid library. Manual production of many tens of TALEs can be achieved using a straightforward 8 hr protocol, with full length sequence-verified plasmids available within a few days. STAR will help drive forward TALE-based applications to complement CRISPR/Cas reagents.

## Results

### A strategy for assembling TALE repeats using enzymatic isothermal assembly

Gibson Assembly (GA) has emerged as a simple method for assembling overlapping DNA fragments, and has been widely adopted as an alternative to restriction enzyme-based cloning^14^,^15^. GA reactions comprise an enzyme cocktail (T5 Exonuclease, Taq Polymerase and Taq DNA ligase) and DNA fragments with at least 16 bp overlapping ends. Reactions are performed at 50 °C for an hour in a single tube reaction^15^.

**Figure 1:**
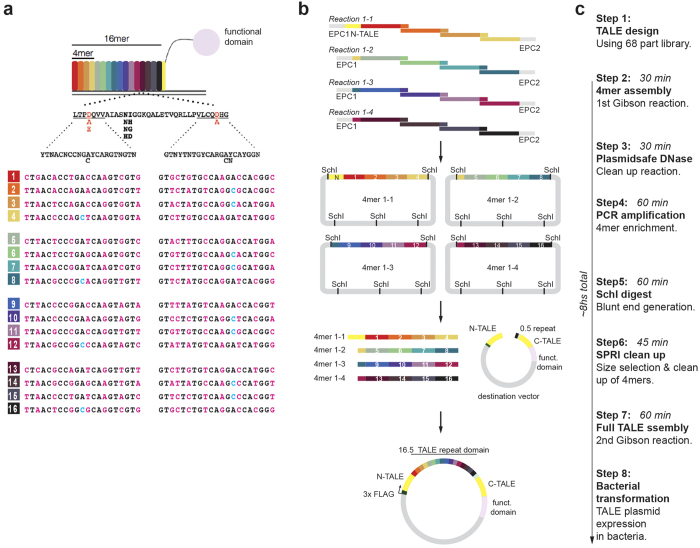
A simple strategy to generate TAL effectors using Gibson Assembly. (**a**) TALE repeat domain of 34 AAs with RVD at position 12 and 13. New sequences for the 21 bp ends were designed using alternative codons that enable position specific assembly. 16 × 2 different ends for TALE repeat domains were created. Each repeat position (1 to 16) has a unique sequence. (**b**) Summary of 8 h assembly of TALE repeat domain with STAR protocol using two separate Gibson assembly reactions; 4 × 1mers, and 4 × 4mers plus vector. (**c**) Reaction times for each step of the TALE assembly using the STAR method.

We considered whether GA could be exploited to assemble TALE DNA binding domains. However, TALE repeats have identical nucleotide end sequences that are incompatible with GA, and these would yield only random concatamers. We therefore exploited codon degeneracy and previously reported natural TALE variant sequences^10^, to re-design coding sequences at 21 bp end-sequences, thereby creating unique ends that could enable position specific assembly (Fig. 1a). Naturally occurring TALEs harbour 12 to 27 repeats, plus a terminal 0.5 repeat that also contributes to target specificity^2^,^10^. TALENs with 16 repeats have been demonstrated to provide unique binding in the mammalian genome with sufficient activity, and so we focussed on developing a strategy to produce TALENs with this repeat size^2^,^10^,^16^.

A single GA reaction with 16 parts would not be reliable. Our strategy uses two distinct assembly reactions; first, repeat monomers (referred herein as 1mers) are assembled into 4mers; then 4 × 4mers are assembled with the vector backbone to create the final 16mer plus 0.5 repeat (Fig. 1b). A 68-part library is all that is required to produce TALEs against any 17 bp target sequences (16 × 4 × 1mers, plus four distinct 0.5mer containing destination vectors). This library can be easily produced and managed by individual laboratories.

The design of a circularised end product in GA generally enriches the reaction for the desired product, as it protects potential free ends from T5 exonuclease. Circularised products can also be further enriched by removal of free DNA using dsDNA nucleases (e.g. Plasmidsafe)^17^. For these reasons we designed the distal 1mers of each 4mer to drive circularisation via inclusion of an end-protective and circularisation sequence (EPC) (Fig. 1b). Shared PCR primers within the EPC facilitate easy amplification of the four separate 4mers (see Supplementary Table S1). Multiple SchI restriction sites enable release of 4mers for the second assembly reaction and EPC sequence removal (Fig. 1b; Supplementary Tables S2 and S3). This simple TALE assembly reaction (STAR) strategy should enable construction of the repetitive sequence encoding the TALE DNA binding domain from a small 68-part library in just 8 hours (Fig. 1c).

### Assembly of TALE 4mers is highly efficient using the STAR method

To test the feasibility of STAR we first generated 64 custom gene plasmids encoding the synthetic TALE repeats flanked by BciVI sites (Supplementary Figure S1 and Supplementary Table S2). Each 1mer was PCR–amplified using a single universal primer pair, and further prepared for GA by BciVI digestion and gel extraction. >2 ug each of each 1mer fragment library was produced in a single day at little cost. Typically, ~20–40 ng was used in each 4mer reaction: a single library preparation is therefore sufficient for production of 4mers that can enable ~100 independent TALE assemblies.

**Figure 2:**
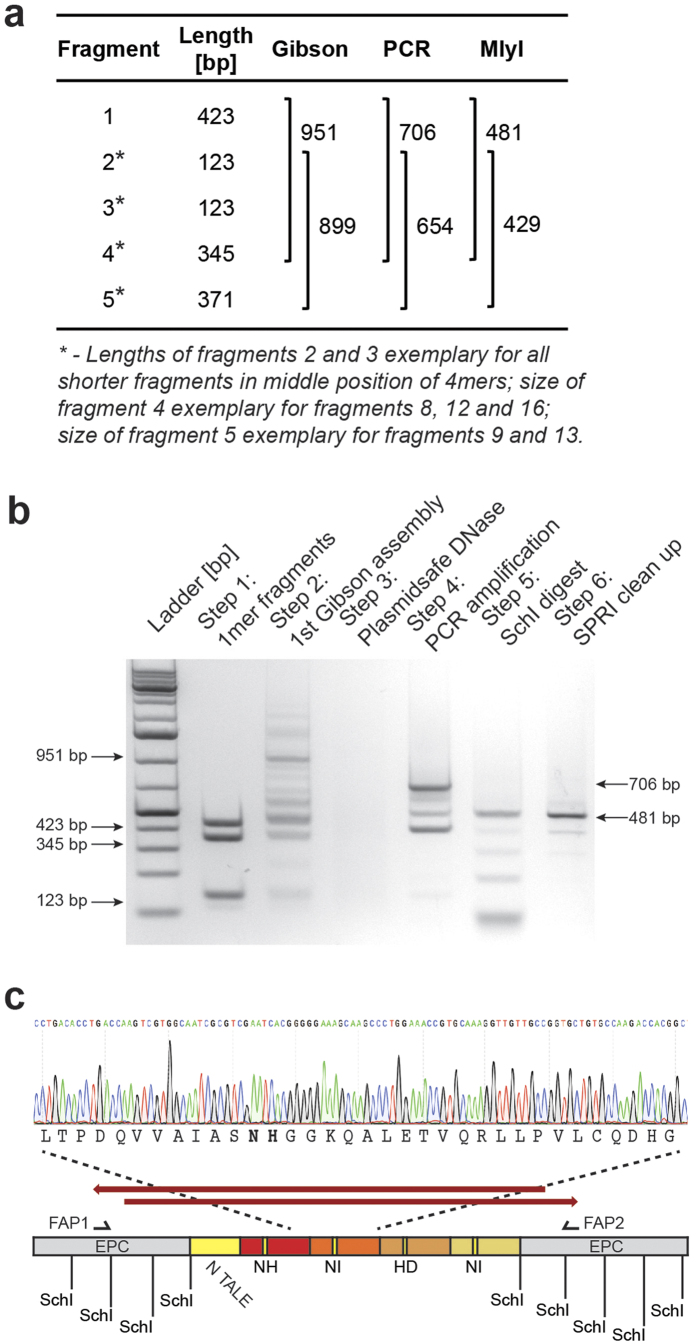
Overview of each step of the 4mer assembly. (**a**) Lengths of 1mer and resulting 4mer fragments at each step of the STAR protocol (before and after digestion and cleanup). (**b**) Starting from 4 × 1mer fragments of varying sizes (423 bp, 345 bp, 123 bp), 4mer DNA fragments are assembled in a first Gibson reaction (951 bp), misassembled products cleaned away in a Plasmidsafe DNase step, correct 4mers amplified via PCR (706 bp) and blunt end fragments for further assembly generated by SchI digest and SPRI clean up (both 481 bp). (**c**) Sequencing strategy for verification of 4mer assembly efficiency and example sequencing trace for Sanger sequencing.

To establish if GA of 1mers into 4mers was efficient and reliable, we generated twenty distinct 4mer GA reactions (random sequence). These would subsequently be used in Steps 2 to 6 of the STAR protocol (Fig. 2a). A 30 min isothermal reaction was found to give the most favourable assembly of 4mers (Supplementary Figure S2) and unassembled linear DNA fragments were indeed efficiently removed using Plasmidsafe DNase, prior to PCR amplification of the 4mer from the circularised template (Fig. 2b; Supplementary Figure S3). As the 4mer assembly is only a 30-minute GA and the EPC sequences are considerably longer than the overhangs between the 1mer products, it is likely that the amount of linear 4mers is higher than the amount of circularized product. Therefore, the PS reaction would clear away most of these products and leave only a small amount of circularized products undetectable by gel electrophoresis, which are recovered in the consecutive PCR amplification. We were not concerned with this as PCR amplification of the correct 4mer was always possible. For blunt end restriction digest of 4mer fragments, the isoschizomer SchI (Figs 1 and 2b) was preferred over MlyI (Supplementary Figures S2 and S3) due to higher reliability (Supplementary Figure S5). Sanger sequencing confirmed all twenty products were the expected 4mer sequence (Fig. 2c).

Using a basic automated liquid handling device, we were able to demonstrate the potential for scale up, as we set up 132 independent 4mer assemblies (sequence targeting proximal promoter region of *SOX2*) and found 98.5% (130/132) had the expected correct sequence following Sanger sequencing. 4mer assembly is therefore highly reliable using the STAR protocol. We calculated that a single 4mer Gibson reaction product can be re-used ~80 times as PCR template. Thus, STAR enables assembly of 4mers with high efficiency and little hands on time.

### Simple and rapid assembly of 16.5mer TALENs and TALE-TFs

We next determined whether the cleaned up 4mers could assemble efficiently into a full-length 16.5mer TALEN or TALE-TF using a second 4 × 4mer GA reaction (Fig. 1b). Initially 4mers were PCR-amplified for subcloning into a pCR-BluntII-TOPO backbone. This enabled retrieval of large amounts of clean 4mer products as a fragment from the digest of the TOPO clones. When using purified 4mers the efficiency of the full 16mer assembly reached up to 42% (n = 4), confirming proof-of-principle for the STAR method. Sequential assembly in which 8mers are formed prior to being incubated together with the destination vector were tested, but did not improve assembly of full-length TAL effectors substantially (33.7% efficiency for sequential vs. 40.5% efficiency for non-sequential assembly; tested with TOPO-cloned 4mers).

**Figure 3:**
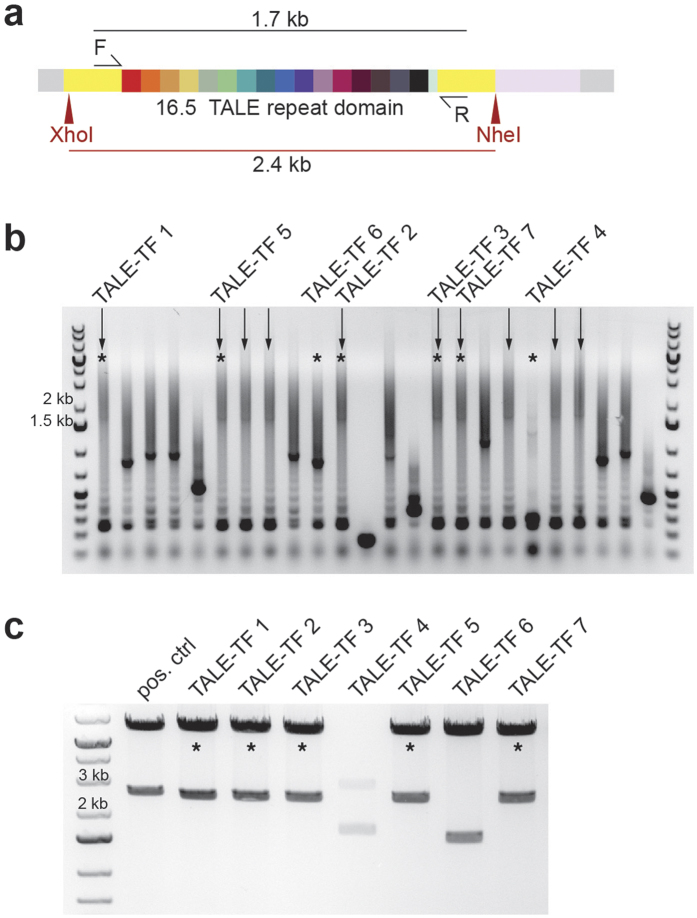
Full-length 16mer TALEs in destination vectors are generated in a second Gibson reaction. (**a**) Schematic of fully assembled TALE into destination vector showing both the primer binding sites for colony PCR and XhoI/NheI restriction sites for digest of TALE-TFs. (**b**) Colony PCR of bacterial colonies expressing a typical TALE-TF reaction. Clones expressing the fully assembled TALE-TF show amplicons of about 1.7 kb length (↓– clone potentially expressing full-length DNA binding domain; * – clones shown in panel c). (**c**) Clones identified in colony PCR are further characterised by restriction digest yielding a 2.4 kb fragment for fully assembled TALE-TFs (* – sequence-verified clones expressing full-length TALE-TF).

We further constructed a pair of TALENs targeting EGFP, which could easily be assayed using EGFP expressing mammalian cells and TALE-TFs designed to bind the upstream proximal promoter region of the human *SOX2* gene, as previously reported (Supplementary Tables S4 and S5)^17^,^18^. Several different experimental parameters were explored for correct 16.5mer assembly and a detailed protocol of final reaction conditions can be found in the Supplementary Methods. To avoid repeated TOPO plasmid cloning of 4mer intermediates, we used freshly PCR-amplified and digested 4mer fragments that were cleaned up using SPRI (Fig. 2b). Alternative cleanup procedures were explored, such as Sephadex spin columns and gel purification; however, these proved unfavourable due to loss of product and lack of scalability. Although not critical, prior to the final GA reaction we typically determined 4mer concentrations and estimated purity using the Agilent TapeStation. Final assembly of 16.5mers into the destination backbone of choice was performed in a 60 min Gibson assembly reaction. 0.5 μl of the reaction mix was used directly for transformation of competent bacteria without any additional cleanup. Bacterial colonies were analysed for full-length TALEs by colony PCR and subsequent restriction digest (Fig. 3), and the overall efficiency of TAL effector assembly as determined by the gold standard Sanger sequencing validation was typically ~5–20% (n = 12; independent TALE-TFs targeting *SOX2* and *ASCL1*). Incorrect sequences are random and include insertions, deletions and base pair exchanges (Supplementary Figure S6). This is most likely due to 4mer impurities present in the final GA reactions. While less efficient than the TOPO cloning strategy, an efficiency of 5–20% is still sufficient for construction of many sequence-verified TALENs or TALE-TFs in three days including screening of bacterial clones and sequence verification using Sanger sequencing (Supplementary Figure S7). In our experience this provides sufficient efficiency to enable production of up to 10 TALEs in a single run. However, the current protocol’s efficiency would need to be further increased, if production of larger numbers of TAL effectors is desired in a single run.

### TALEN and TALE-TF plasmids generated using STAR are functional

**Figure 4:**
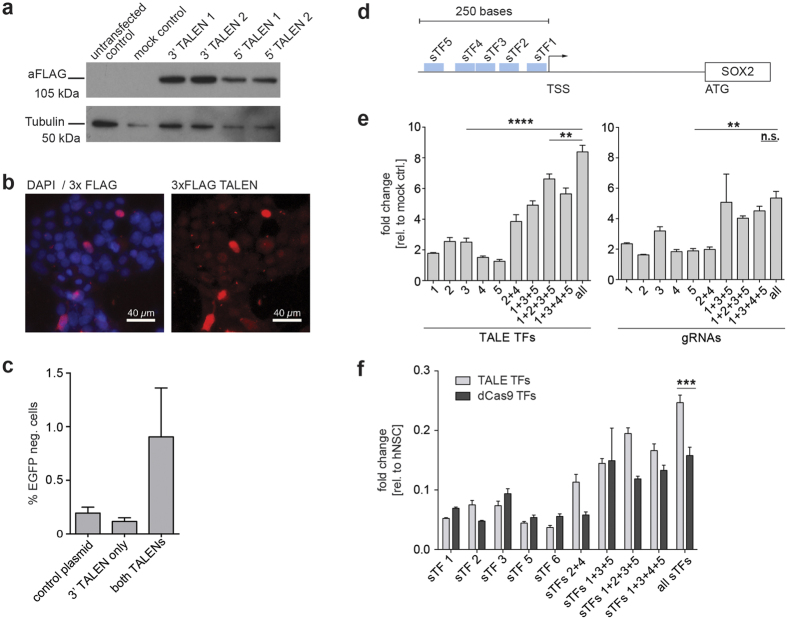
Full-length TALENs and TALE-TFs are expressed and functional. (**a**) Correct expression of TALE constructs is seen in target cells using western blot detecting full-length TALEN constructs of 110 kDa (detection of 3× FLAG in N-terminus of TALE). TALEN 1 and 2 target the same sequence and represent technical replicates assembled in two different reactions. (**b**) Immunocytochemistry of TALE-transfected cells shows nuclear expression of TALEs. (**c**) Quantification of flow cytometry analysis of EGFP levels in mNSCs transfected with EGFP TALENs assembled with the STAR method. Expression of both 3′ and 5′ TALEN in mNSCs induces reduction in EGFP levels. (**d**) sTFs targeted a region −5 to −230 bp from the predicted TSS of *SOX2*. (**e**) TALE-TFs against the proximal promoter region of *SOX2* induce tuneable transcriptional activation in 293 cells with sTF pools inducing significantly higher mRNA levels than single sTFs (****P < 0.0001; **P < 0.01). (**f**) Normalising mRNA levels in target cells against human NSCs as gold standard, we reached up to 25% of *SOX2* levels expressed in hNSCs. Pools of TALE-TFs lead to higher mRNA levels of *SOX2* in target cells compared to dCas9-TFs (P < 0.001).

We next tested whether the TALENs and TALE-TFs generated above were expressed in mammalian cells by transfection of HEK293 cells. All TALE constructs express an N-terminal triple FLAG-tag and nuclear localisation signal. 72 hr post-transfection we detected the correct sized protein using western immunoblotting, and nuclear localisation was confirmed using immunocytochemistry (Fig. 4a,b).

The pair of TALENs targeting EGFP were tested for their functionality in mouse neural stem cells harbouring a CAG-EGFP transgene, and quantified using flow cytometry (Fig. 4c). Single TALENs did not reduce EGFP-levels, confirming the low background of FokI homodimers. When expressing paired TALENs, there was a clear shift towards lower EGFP-levels in target cells. The amount of EGFP-negative cells was increased ~10-fold in cells targeted with a full pair of EGFP-TALENs, proving functionality of TALENs generated with the STAR protocol. We further validated these results in another adult NS cell line using both flow cytometry and Sanger sequencing of the EGFP^neg^ population (Supplementary Figure S8).

We next assessed whether the STAR-generated TALE-TFs designed for activation of *SOX2* were functional. We targeted these to DNA sequences <250 bp upstream of the *SOX2* transcription start site (TSS) and already published by Cheng *et al*.^18^ to activate *SOX2* expression with dCas9-TFs (Fig. 4d). Target cells were transfected with single TALE TFs and dCas9-TFs or pools of these synthetic transcription factors (sTFs) to test for tuneable gene activation. Analysing transcript levels 96 h post-transfection showed up to 8-fold activation of *SOX2* compared to cells transfected with a TALE-VP64 lacking the DNA binding domain (Fig. 4e). Using TALE-TF pools, we were able to reach gene activation of up to 25% of physiological *SOX2* levels seen in human NSCs (Fig. 4f). Moreover, comparing activation of *SOX2* using dCas9-TFs sharing the same target sites as TALE-TFs, we detected 1.6-fold higher activation using TALE-TFs.

## Discussion

Here we report a new approach to TAL effector assembly, which we term STAR (simple TALE assembly reaction). STAR enables production of TALENs, TALE-TFs and TALE-CEs using a strategy that will meet the demand for a user-friendly and simple, cheap and rapid production of different TALE-based reagents at low to medium throughput. We have focussed on mammalian cells, but STAR could readily be modified for other organisms or cell lines. Also, with a starting library of only 68 parts, enough flexibility is granted to re-engineer or design any new improvements or desired changes to the architecture of repeats. This could also allow changes to the length of the TALE-DBD, as STAR was designed specifically with the production of only 16.5 repeats in mind.

Introducing TALE variant sequences into the TALE repeats to generate unique ends allowing Gibson assembly, we introduced previously published ‘non-RVD variations’^19^. These have previously been reported to result in increased TALEN activities. TALEs assembled with the STAR protocol might therefore display advantageous activities compared to TALEs not incorporating non-RVD variations.

TALE assembly using the FairyTALE Golden Gate reactions has been reported to be more efficient than STAR^13^. However, Golden Gate reactions can still be highly variable, with the need of optimising cloning reactions and require greater initial investment in library production, which often results in more expensive and time-consuming setup costs. Furthermore, for TALE-TFs absolute purity may not be required, as the incorrectly assembled TALE-TF products might not interfere with transcriptional activation of the target gene and would be unlikely to have significant off target effects; thus, for some applications production of TALE-TFs could be carried out using polyclonal plasmid preparations. TALE assembly efficiencies using the scalable STAR protocol range between 5–20% and therefore still require identification of bacterial colonies using colony PCR. We identified the purity of 4mer constructs to be of importance for an increased rate of successful full-length assembly. Therefore, if higher assembly efficiencies are required, TOPO-subcloning of 4mers can be performed, thereby prolonging the STAR protocol by ~1d, but increasing the assembly efficiency to ~40%.

When modulating gene expression, it may be desirable to repress target genes or alter the chromatin state at target sites. Therefore, diverse effector domains would be useful to incorporate in future iterations of STAR. With this in mind we engineered a toolbox of distinct activators, repressors and chromatin modifiers by altering the TALE destination vector to be compatible with shuttling in any desired functional domain. An RFP cassette flanked with GG cloning sites was engineered into the STAR mammalian expression vectors. Thus, through a simple GG reaction any new functional domain of interest can be inserted and easily screened (white-/red-screening). We confirmed that GG-based exchange of domains was highly efficient by production of TALE-KRAB and TALE-p300core destination vector constructs. This toolbox of destination vectors therefore provides a convenient route for producing bespoke functional effector domains, and for screening novel effector domains.

Future use of TALE-TFs and chromatin editors is likely to hold great value in mammalian stem cell and synthetic biology for the creation of orthogonal circuits, or for use in reprogramming gene regulatory networks to control cell fate and study cell biology. Generating large numbers of TALEs may have particular use in the construction of TALE-TFs for ‘tuneable’ expression of target genes across large dynamic ranges^20^. Future studies should now be directed at determining how TALE-TFs and dCas9-TFs compare in performance across a larger set of loci and how these can be used in combinations for modulating cell behaviours. Routine production of TALE-TFs using STAR will complement the emerging CRISPR/Cas9-based reagents and open up new possibilities for a myriad of applications where gene regulatory circuits need to be interrogated or engineered.

## Methods

### 1mer custom gene library design and amplification

Custom genes (Life Technologies) encoding 1mer repeat sequence were designed with improved RVDs reported by Cong *et al*.^21^ Each 3′ end 21 bp sequence is identical to that of the respective adjacent repeat enabling assembly with GA. End protector and circularisation sequence (EPC) was derived from stuffer fragment sequence of standard pCAG expression vector. Library preparations were performed using Herculase II DNA polymerase (Agilent Technologies). PCR products were pooled and purified with QIAquick PCR Purification Kit (Qiagen). Fragments were digested with BciVI for 12 h and gel-purified (QIAquick Gel Extraction Kit, Qiagen). All plasmids required for STAR are available upon request or in future via Addgene.

### TAL effector mammalian expression vector construction

TALE destination vectors were derived from modification of the pCAG-GFP-IRES-puro expression plasmid (gift from Austin Smith). We removed a BciVI restriction site and exchange of GFP cassette for N and C terminal fragments (Δ152/+63) of the TALE plus the FokI domain using XhoI and NotI restriction digest, created four distinct vectors, each harbouring a different 0.5mer half repeat (0.5mer exchange with NruI/HpaI restriction digest and blunt end ligation; T4 DNA Ligase, NEB): pTALEN-STAR-A, pTALEN-STAR-T, pTALEN-STAR-G, and pTALEN-STAR-C. For TALE-TF backbones, FokI was replaced with VP64 domain (custom gene, Life Technologies) using HpaI/PflFI-based cloning: pTALE-STARVP64-A, pTALE-STARVP64-T, pTALE-STARVP64-G, and pTALE-STARVP64-C. TALE-RFP entry vector was constructed by exchange of VP64 domain with RFP cassette (RFP expression plasmid kind gift of Yizhi ‘Patrick’ Cai) using SacI-/BspEI-based restriction digest and ligation.

### 4mer assembly (1st Gibson reaction)

Gibson reaction mix was produced as previously reported^15^. 4mers were assembled with an equimolar 1mer ratio and an overall DNA input of 120 ng. Reactions were incubated at 50 °C for 30 min with subsequent clean up using Plasmidsafe DNAse (Epicentre). 0.5 μl of final reaction mix was used for PCR amplification with Herculase II DNA Polymerase (Agilent Technologies). PCR amplicons were digested with FastDigest SchI (Life Technologies) directly in PCR buffer for 1 h at 37 °C plus 5 min heat-inactivation at 80 °C. Subsequent 4mer purification utilised Agencourt AMPure XP (Beckman-Coulter) with PEG gradient size selection (final concentration of 7.5% PEG-8000 and 0.9 M NaCl)^22^.

### 16mer assembly (2nd Gibson reaction)

For full-length assembly of 16mer repeat domain, 4mers were used at equimolar ratios. Destination vectors were linearised prior to assembly using BciVI (alternative digest if BciVI sites are present in functional domain see Supplementary Methods). Fragments were Gibson assembled for 1 h at 50 °C. 0.5 μl of the Gibson mix containing fully assembled plasmids was directly transformed into 25 μl chemically competent bacteria and plated for overnight growth.

### Plasmid validation

Colony PCR was performed with DreamTaq polymerase (Thermo Scientific) and DNA of positive colonies was extracted using QIAprep Spin Miniprep Kit (Qiagen). TALENs and TALE-TFs were verified using a XhoI/NotI and XhoI/NheI restriction digest, respectively. Full-length products were sequence-verified with Sanger sequencing.

### Golden Gate exchange of functional domains in pTALE-STAR_RFP entry vector

For Golden Gate exchange of the RFP expression cassette a total reaction volume of 10 μl comprising 1,000U T4 Ligase (NEB), 7× BSA, 5U Esp3I, 1 μl Buffer Tango (Thermo Fischer), 50 ng of the respective pTALE-STAR_RFP entry vector and 100 ng of the respective expression vector with the functional domain of choice were incubated as follows: 30 cycles of 37 °C for 5 min and 16 °C for 10 min; 50 °C for 5 min, 80 °C for 10 min. Correctly assembled vectors were identified by red/white screening in *E. coli*.

### Preparation of gRNAs

gRNAs were ordered as single stranded oligonucleotides from Sigma-Aldrich, mixed and annealed. Oligonucleotides were phosphorylated using T4 PNK (Thermo Scientific) and ligated into a U6-promoter backbone (kind gift of William Skarnes laboratory, Sanger Institute) using T4 Ligase (Thermo Scientific). For activation the previously reported pAC91-pmax-dCas9VP64 plasmid^18^ was acquired via Addgene (plasmid #48223).

### Cell culture and transfection

Mouse neural stem (NS) cells were cultured as described before^23^. HEK293 cells were grown in GMEM supplemented with 10% FCS. Transfection was performed using PEI for 293 cells and nucleofection using an Amaxa 4D-Nucleofector™ System (Lonza) for NS cells, respectively. For both transfection protocols, an overall DNA amount of 1–2 μg was used. When transfecting TALE-TFs, the overall amount of DNA was split between the respective numbers of constructs used. For dCas9-TFs, the ratio of dCas9-VP64 plasmid to gRNAs was 1:1 (with gRNAs split evenly depending on numbers).

### Immunocytochemistry

Cells were stained with anti-FLAG tag antibody (eBioscience) and goat secondary Alexa594-coupled antibody (Life Technologies) was applied with DAPI nuclear counterstain (Sigma-Aldrich). Images were acquired using a Zeiss Axio Observer wide-field fluorescence microscope and AxioVision software.

### Quantitative real-time RT-PCR

RNA was extracted using RNeasy Plus Mini Kit (Qiagen) for cDNA synthesis with SuperScript^®^ III First-Strand Synthesis SuperMix (Life Technologies) and qPCR using TaqMan assays (Applied Biosystems) and a QuantStudio™ 7 Flex Real-Time PCR system (Life Tech).

### Flow cytometry

Cells were stained with 7-AAD as Live/Dead marker and analysed using the BD Accuri™ C6 Flow Cytometer (BD Biosciences). Subsequent analysis of flow cytometric data was performed with FlowJo Analysis Software (TreeStar).

## Additional Information

**How to cite this article**: Gogolok, S. *et al*. STAR: a simple TAL effector assembly reaction using isothermal assembly. *Sci. Rep.* **6**, 33209; doi: 10.1038/srep33209 (2016).

## Supplementary Material

Supplementary Information
