# Supplementary material for: STAR: a simple TAL effector assembly reaction using isothermal assembly
Source: Sci Rep. 2016 Sep 12;6:33209. doi: 10.1038/srep33209 (PMC5018962; doi:10.1038/srep33209)
Supplement: Supplementary Information [file srep33209-s1.pdf]

STAR: a simple TAL effector assembly reaction using isothermal assembly

Sabine Gogolok[1], Claudia Garcia-Diaz[1] and Steven M. Pollard[1,*]

[1] MRC Centre for Regenerative Medicine, University of Edinburgh, Edinburgh bioQuarter, 5 Little France Drive, Edinburgh EH16 4UU, UK

* To whom correspondence should be addressed:

Tel: +44 (0)131 6519544; Fax: +44 (0)131 6519501;

Email: steven.pollard@ed.ac.uk

1mer custom genes

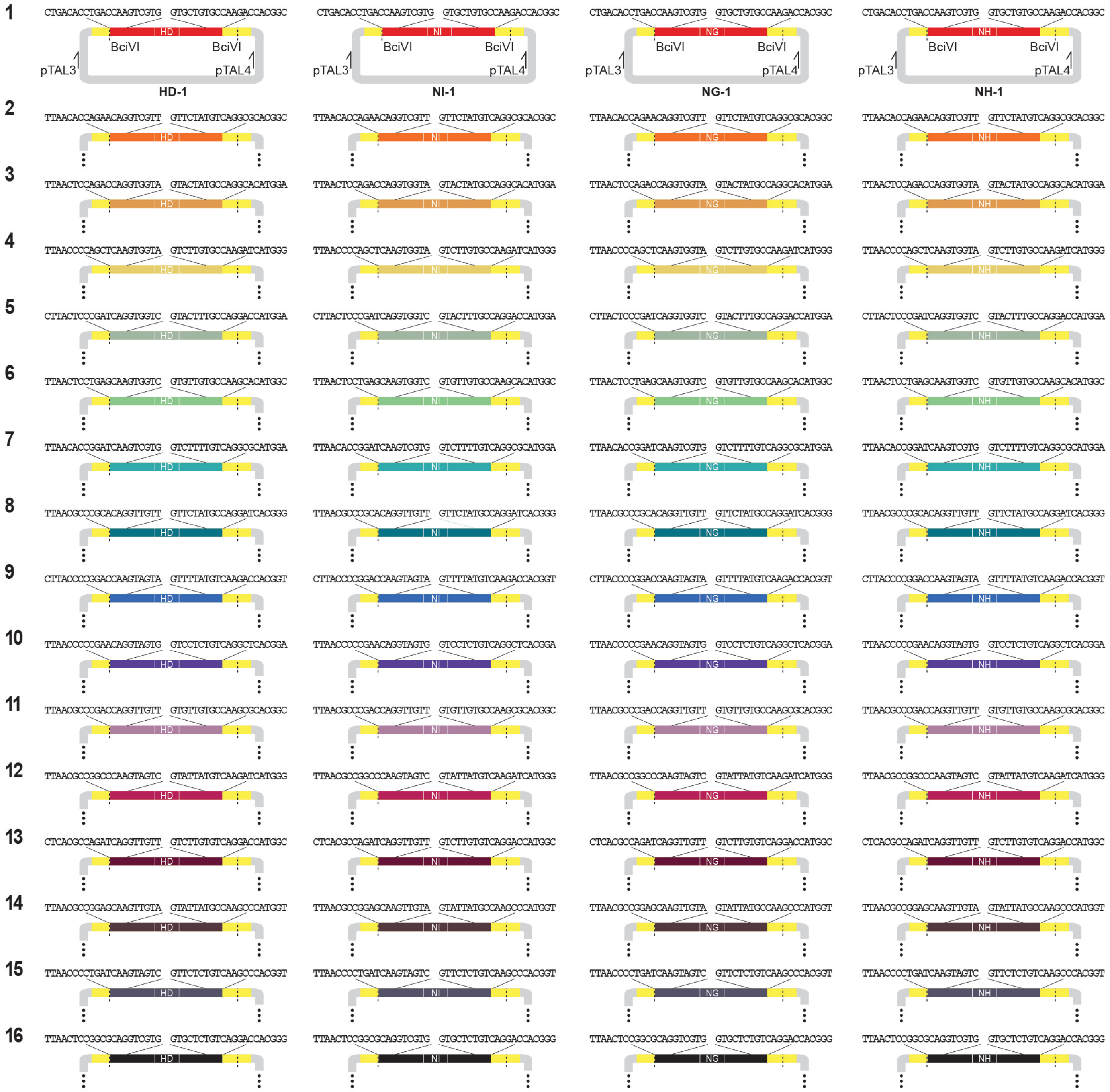

Destination vectors

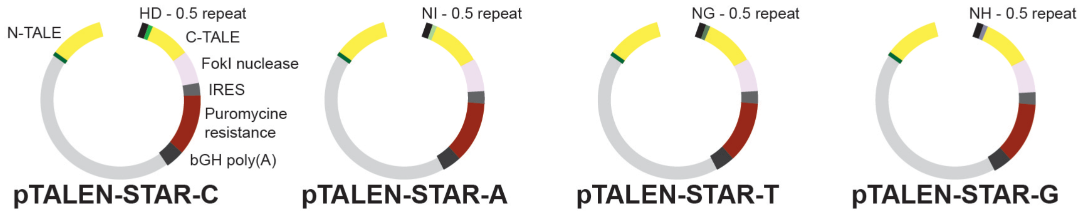

Supplementary Figure S1 | Overview of 68-part library for STAR method. Monomer repeats were ordered from Life Technologies as custom genes (1mer custom genes) and were received in universal plasmid backbones pMA and pMA-t, respectively (grey generic backbone). Universal primer binding sites in backbone (pTAL3/4) allow 1mer amplification and subsequent BciVI digest of amplicons generates final 1mer library. For assembly of full 16.5-repeat TAL effectors, 4 different destination vectors harbouring the functional TALE domains and the final 0.5 repeat are included in the library, therefore presenting a 68-part library for STAR.

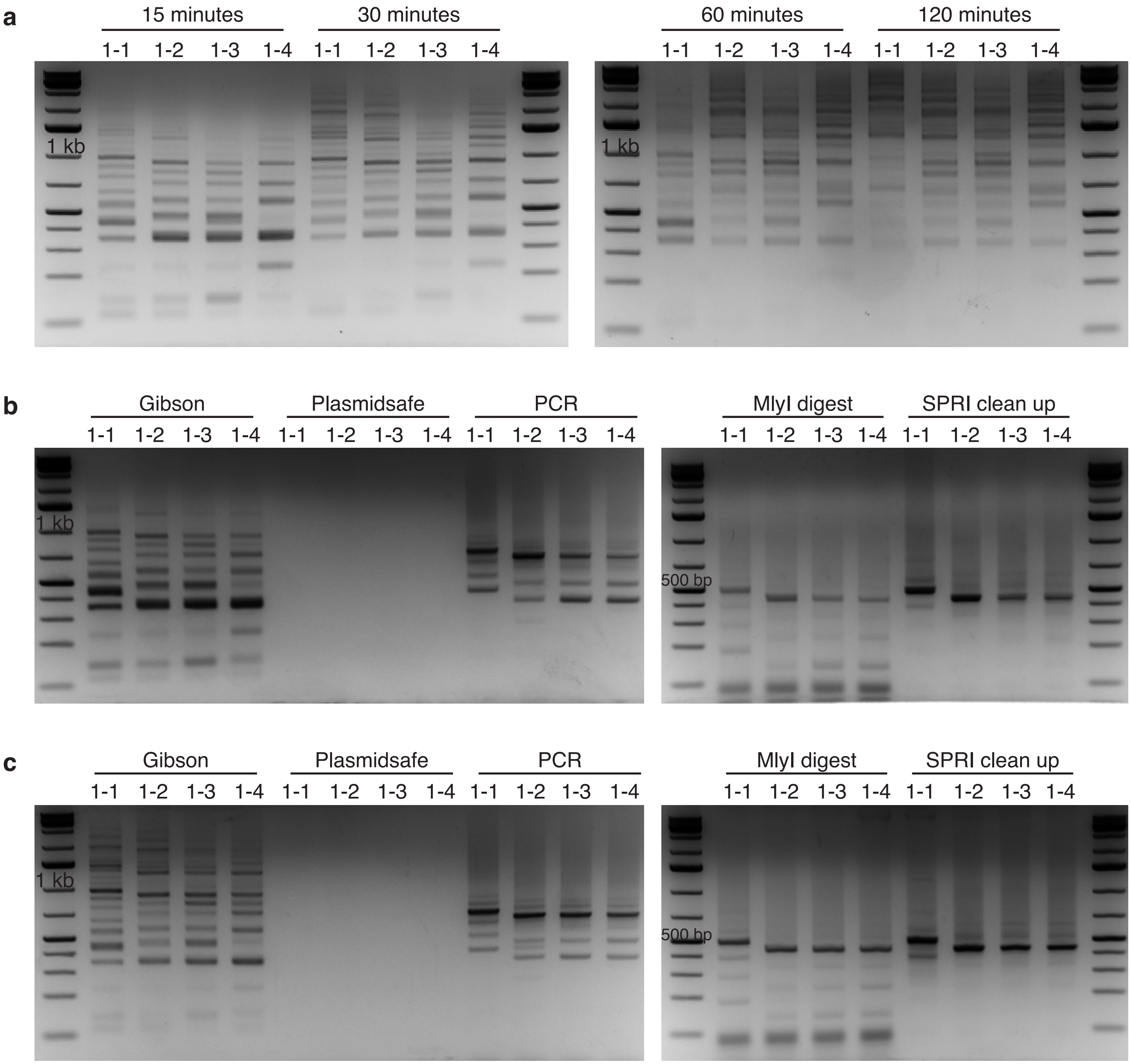

Supplementary Figure S2 | Optimisation of Gibson reaction times for 4mer assembly.
(a) 15-minute Gibson reaction times are sufficient to detect correct 4mers at 951 bp and 899 bp, respectively. However, considerable amounts of unassembled 1mers remain with a 30-minute assembly giving better yield. 60 min and 120 min reactions lead to increased proportions of undesirable concatemerised products, which might contaminate downstream reactions. Comparison of 15 min Gibson reaction (b) and 30 minute Gibson reaction (c) reveal higher yield of clean 4mers after 30 min with no increased background (4mers at 481 bp and 429 bp, respectively).

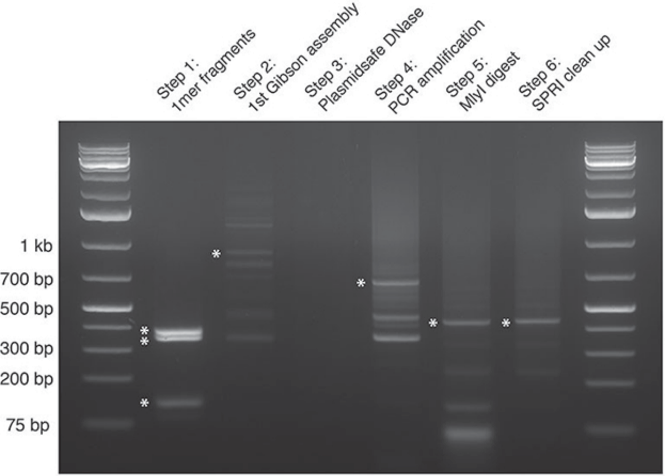

Supplementary Figure S3 | Overview of STAR assembly exemplified for 4mer 1-2. Starting from four 1mer fragments of varying sizes (371 bp, 345 bp, 123 bp), 4mer DNA fragments are assembled in a first Gibson reaction (899 bp), misassembled products cleaned away in a Plasmidsafe DNase step, correct 4mers amplified via PCR (654 bp) and blunt end fragments for further assembly are generated by MlyI digest and SPRI clean up (both 429 bp).

Supplementary Figure S4. Vector map of a STAR TALEN vector.

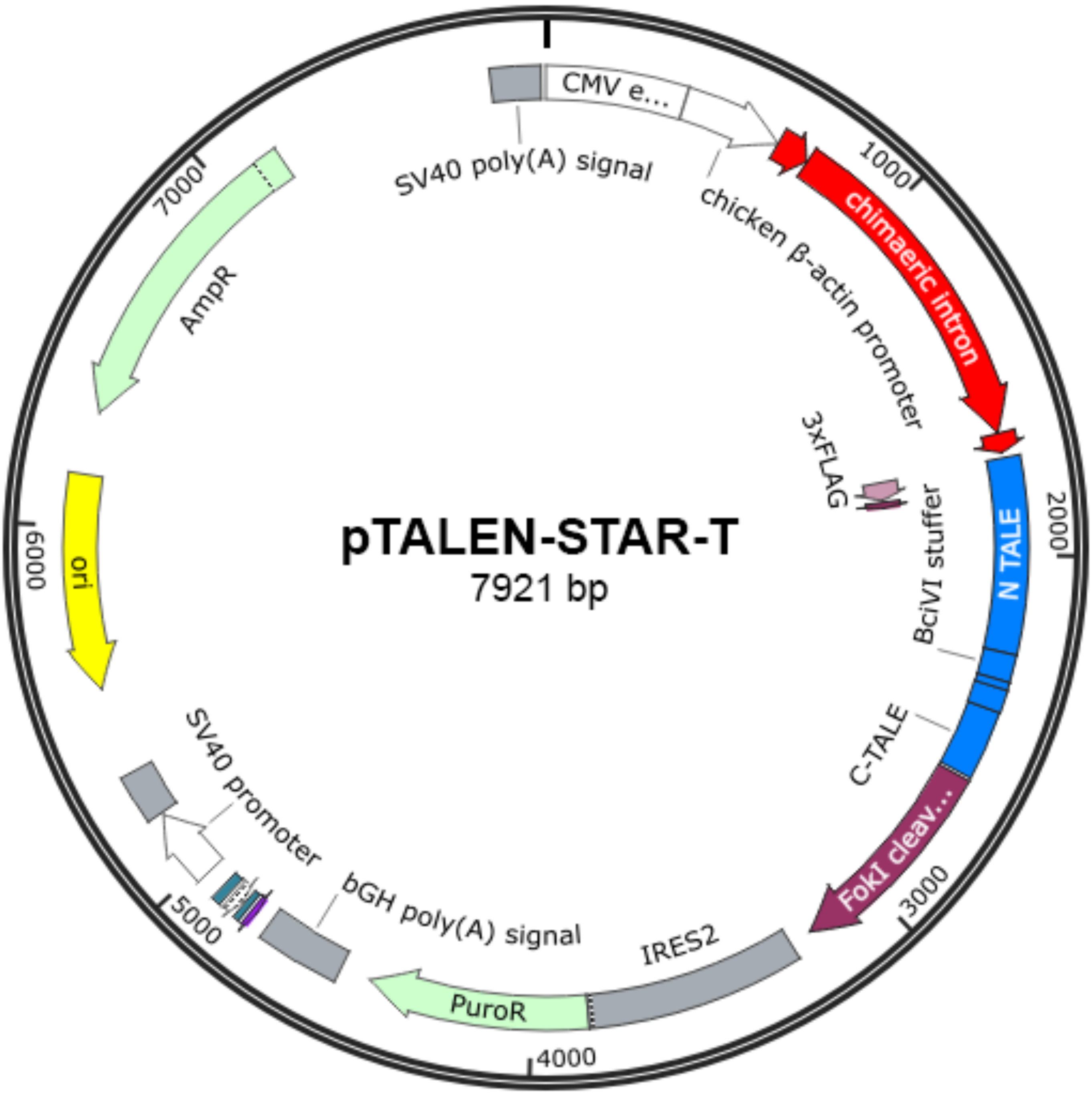

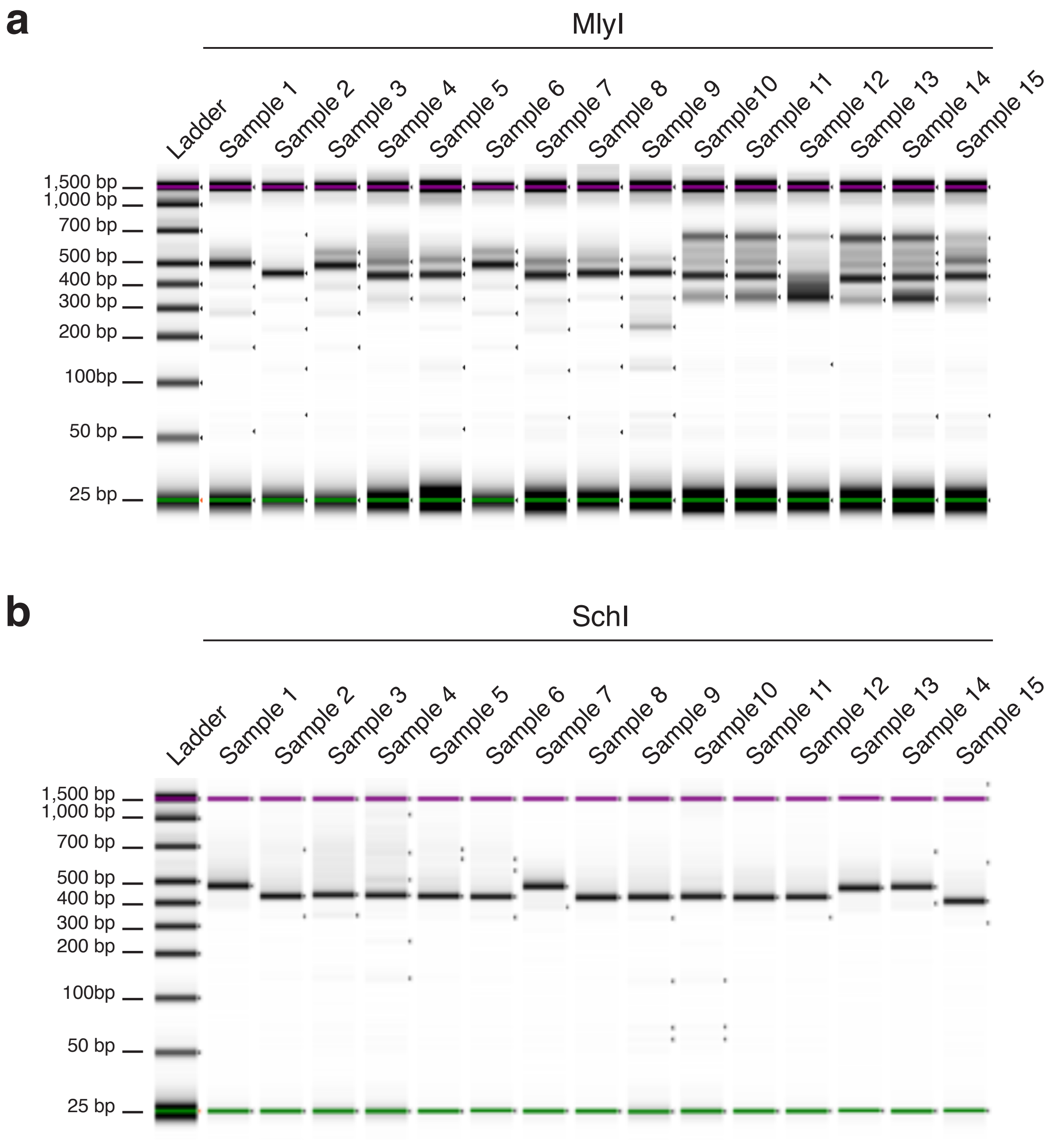

Supplementary Figure S5 | Comparison of SchI and MlyI for 4mer digest. TapeStation traces show 4mer fragments after restriction digest and clean up of small by-products of the digest. This is to focus entirely on remaining fragments of >500bp length indicating incomplete digest. (a) MlyI digest proved to be unreliable even after overnight restriction digest, as seen in samples 3 to 15 to varying degrees. (b) The isoschizomer SchI was tested and found to give reliable results after a restriction digest as short as 1h, exemplified by the presence of only the clean 4mer fragments of 481 bp and 429 bp length, respectively.

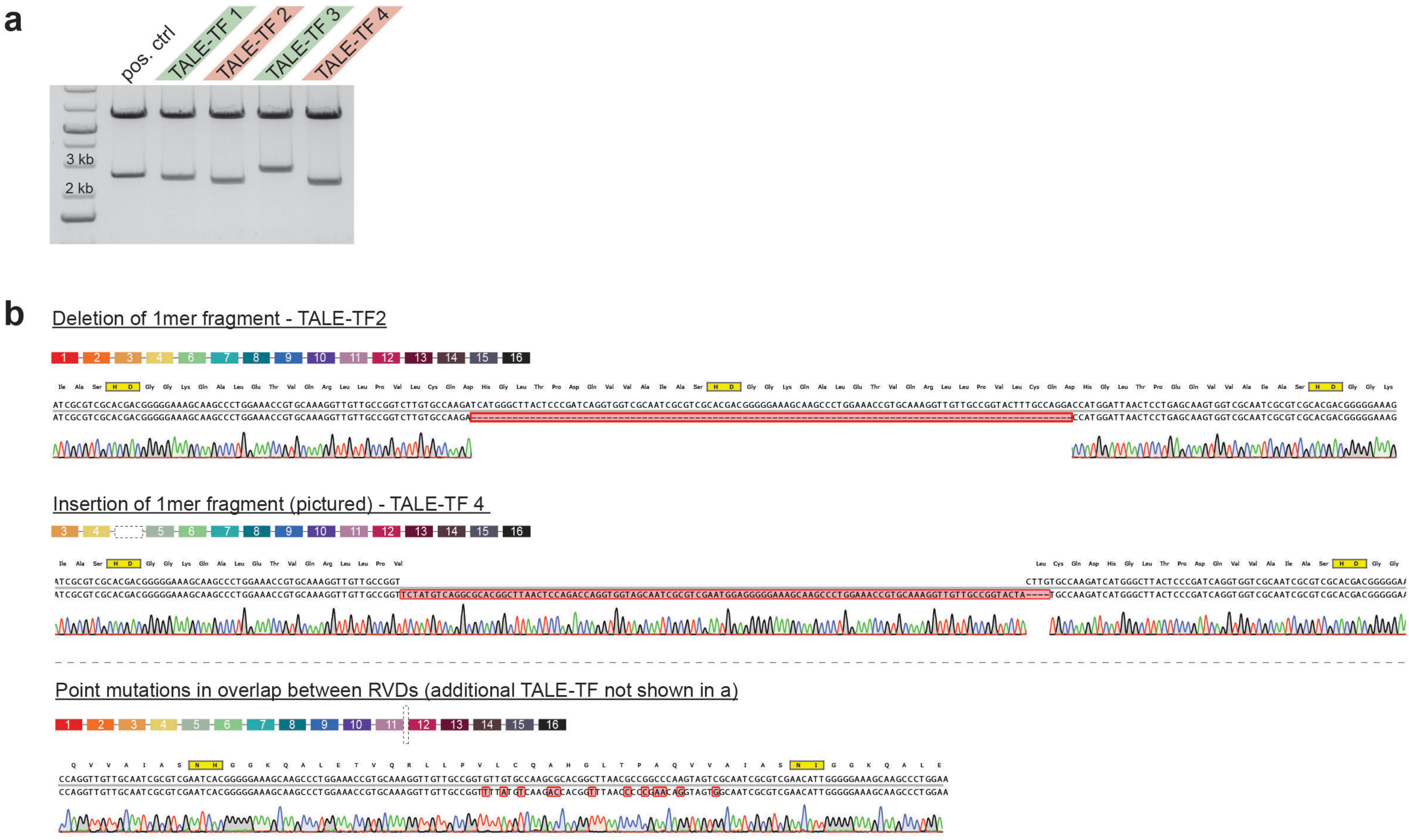

Supplementary Figure S6 | Sanger sequencing of full-length TALEs - Examples of incorrect assembly products. (a) Restriction digest of full-length TALE-TFs shows differences in size compared to 2.4kb fragment for positive control. TALE-TFs highlighted in green were confirmed to have the correct sequence in Sanger sequencing, TALE-TFs highlighted in red showed smaller products in digest and are further analysed in panel (b). (b) Examples of incorrect assembly products discovered in Sanger sequencing of TALE-TFs. Possible misassembly products included deletion of 1mer fragments, insertion of 1mer fragments, point mutations in the overlaps between 1mer RVDs or a combination of these (not pictured). Incorrect assembly was random and occured at any position in the 16mer.

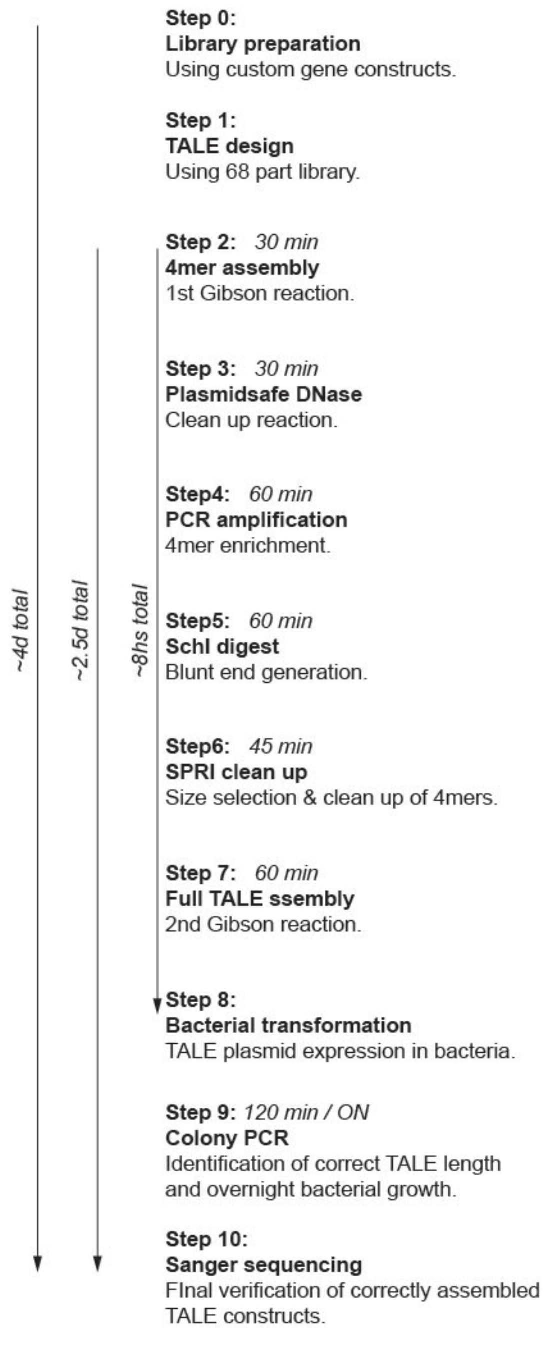

Supplementary Figure S7 | Overall timeline for STAR including TALE verification. Assembly of sequence-verified TAL effector constructs requires 2.5 days in total, including colony PCR for screening of correctly assembled bacterial clones and sequence verification of finale TALE constructs by Sanger sequencing.
If the initial 1mer library needs to be synthesised, this adds another 1.5 days to the protocol.

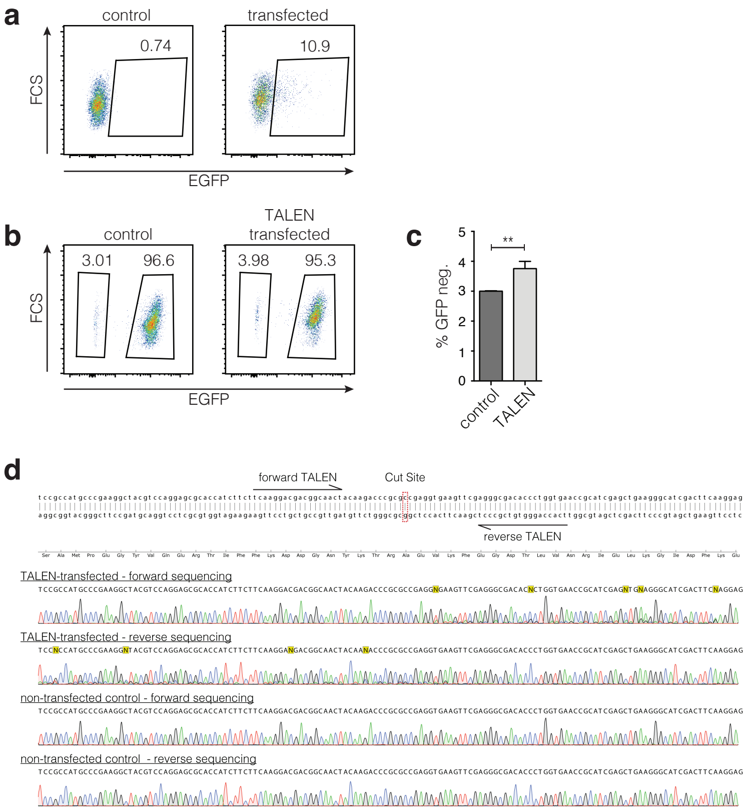

Supplementary Figure S8 | EGFP TALEN efficiency in adult mNS cells. To verify functionality of EGFP-TALENs their performance was tested in Rosa26-EGFP mouse NS cells. (a) Transfection efficiencies of ~10% were tested with a mCherry-expression plasmid as control. (b) Exemplary dot plot of an increase of EGFP-negative cells after TALEN transfection of target cells. (c) Quantification of flow cytometry data shows a significant increase of EGFP-negative target cells 4 days after TALEN transfection. Data shows independent biological replicates using different TALEN preparations. (n=4; $p < 0.01$) (d) EGFP-negative fraction was isolated via flow cytometry and targeted locus analysed for indel formation by Sanger sequencing. TALEN-transfected population shows indel formation indicated by a mixed sequencing trace.

Supplementary Figure S9. Vector map of a STAR TALE TF vector.

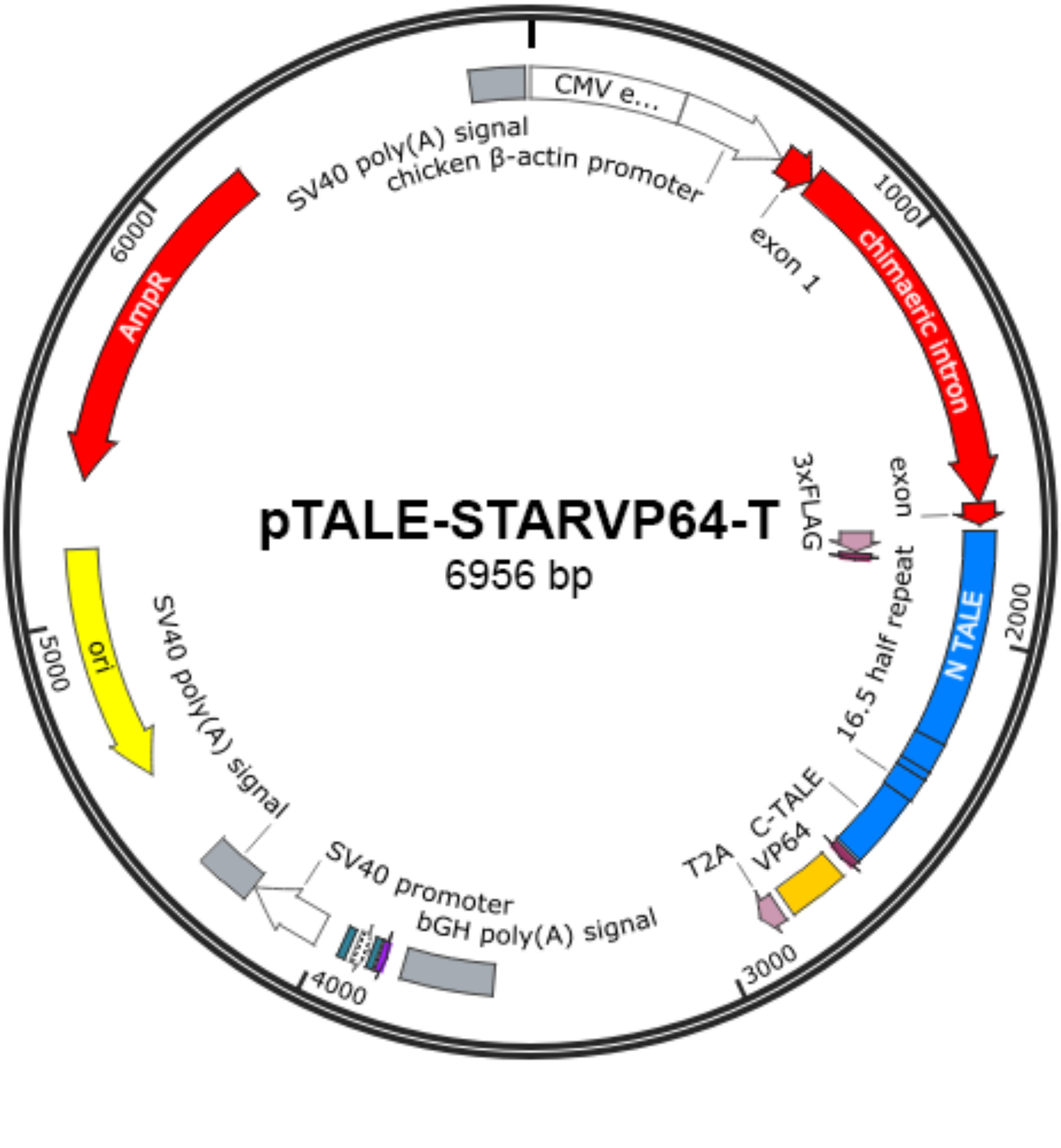

Supplementary Figure S10. Vector map of a STAR RFP entry vector.

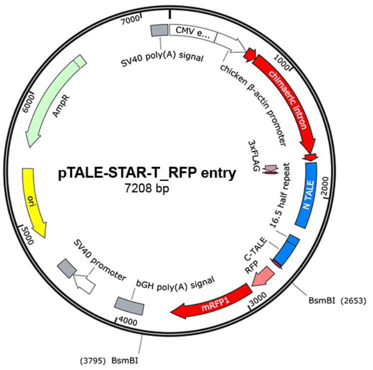

Supplementary File S1. Sequences of STAR TALEN destination vectors and component parts.

Sequences of complete STAR vectors and component parts

Below is the sequence for a STAR vector for TALEN assembly coding for FokI endonuclease.

General features and positions:

Feature	Position	Length [bp]
N TALE	1737..2264	528
3xFLAG	1740..1805	66
SV40 NLS	1812..1832	21
BciVI stuffer	2265..2338	74
3d16 Gibson overlap	2339..2359	21
16.5 half repeat	2360..2419	60
C TALE	2420..2608	189
FokI cleavage domain	2615..3202	588
IRES2	3262..3847	586
Pac	3848..4447	600
AmpR	6311..7171	861

pTALEN-STAR-T (NG) plasmid – a corresponding plasmid map is shown in Suppl. Fig. S4:

gacattgattattgactagttattaatagtaatcaattacggggtcattagttcatagcccatatatggagttccgcgttacat
aacttacggtaaatggcccgcctggctgaccgcccaacgacccccgcccattgacgtcaataatgacgtatgttccca
tagtaacgccaatagggactttccattgacgtcaatgggtggactatttacggtaaactgcccacttggcagtacatcaa
gtgtatcatatgccaagtacgccccctattgacgtcaatgacggtaaatggcccgcctggcattatgcccagtacatga
ccttatgggactttcctacttggcagtacatctacgtattagtcatcgctattaccatgggtcgaggtgagccccacgttctg
cttcactctccccatctcccccccctccccacccccaattttgtatttatttattttttaattattttgtgcagcgatgggggcgg
gggggggggggcgcgcgccaggcggggcggggcggggcgaggggcggggcggggcgaggcggagaggt
gcggcggcagccaatcagagcggcgcgctccgaaagtttccttttatggcgaggcggcggcggcggcggccctata
aaaagcgaagcgcgcggcgggcgggagtcgctgcgttgccttcgccccgtgccccgctccgcgccgcctcgcgcc
gcccgccccggctctgactgaccgcgttactcccacaggtgagcgggcgggacggcccttctcctccgggctgtaatt
agcgcttggtttaatgacggctcgtttcttttctgtggctgcgtgaaagccttaaagggctccgggagggccctttgtgcgg
ggggagcggctcgggggtgcgtgcgtgtgtgtgtgcgtggggagcgccgcgtgcggcccgcgctgcccggcgg
ctgtgagcgctgcgggcgcggcgcggggctttgtgcgctccgcgtgtgcgcgaggggagcgcggccgggggcggt
gccccgcggtgcggggggggctgcgaggggaacaaaggctgcgtgcggggtgtgtgcgtgggggggtgagcagg
gggtgtgggcgcggcggtcgggctgtaacccccccctgcacccccctccccgagttgctgagcacggcccggcttcg
ggtgcggggctccgtgcggggcgtggcgcggggctcgccgtgccgggcggggggtggcggcaggtgggggtgcc
gggcggggcggggccgcctcgggccggggagggctcgggggaggggcgcggcggcccccggagcgccggcgg
ctgtcgaggcgcggcgagccgcagccattgccttttatggtaatcgtgcgagagggcgcagggacttcctttgtcccaa
atctggcggagccgaaatctgggaggcgccgccgcacccctctagcgggcgcgggcgaagcggtgcggcgcc
ggcaggaaggaaatgggcggggagggccttcgtgcgtcgccgcgccgccgtccccttctccatctccagcctcggg

gctgccgcagggggacggctgccttcgggggggacggggcagggcggggttcggcttctggcgtgtgaccggcgg
ctctagagcctctgctaaccatgttcatgccttcttctttttcctacagctcctgggcaacgtgctggttgttgtgctgtctcatc
attttggcaaagaattcctcgaggccgccaccATGGACTACAAAGACCATGACGGTGATTATAA
AGATCATGACATCGATTACAAGGATGACGATGACAAGATGGCCCCCAAGAAGAA
GAGGAAGGTGGGCATTCACCGCGGGGTACCTATGGTGGACTTGAGGACACTCG
GTTATTCGCAACAGCAACAGGAGAAAATCAAGCCTAAGGTCAGGAGCACCGTC
GCGCAACACCACGAGGCGCTTGTGGGGCATGGCTTCACTCATGCGCATATTGT
CGCGCTTTCACAGCACCCTGCGGCGCTTGGGACGGTGGCTGTCAAATACCAAG
ATATGATTGCGGCCCTGCCCGAAGCCACGCACGAGGCAATTGTAGGGGTCGGT
AAACAGTGGTCGGGAGCGCGAGCACTTGAGGCGCTGCTGACTGTGGCGGGTG
AGCTTAGGGGGCCTCCGCTCCAGCTCGACACCGGGCAGCTGCTGAAGATCGC
GAAGAGAGGGGGAGTAACAGCGGTAGAGGCAGTGCACGCCTGGCGCAATGCG
CTCACCGGGGCCCCCTTGAAC**CTGCAGGATACGTTTATCAGTGATAAAGTGTC**
AAGCATGACAAAGTTGCAGCCGAATACAGTGGTATCCTGCAGGTGCTCTGTCA
GGACCACGGGCTTACGCCACAACAGGTGGTGGCGATTGCATCTAATGGAGGCG
GACGCCCTGCCTTGGAG*TCCATCGTAGCCCAATTGTCCAGGCCCGATCCCGCG*
TTGGCTGCGTTAACGAATGACCATCTGGTGGCGTTGGCATGTCTTGGTGGACG
ACCCGCGCTCGATGCAGTCAAAAAGGGTCTGCCTCATGCTCCCGCATTGATCAA
*AAGAACCAACCGGCGGATTCCCGAGAGAACTTCCCATCGAGTCGCG*GGATCCC
AACTAGTCAAAAGTGAACTGGAGGAGAAGAAATCTGAACTTCGTCATAAATTGAA
ATATGTGCCTCATGAATATATTGAATTAATTGAAATTGCCAGAAATTCCACTCAG
GATAGAATTCTTGAAATGAAGGTAATGGAATTTTTTATGAAAGTTTATGGATATAG
AGGTAAACATTTGGGTGGATCAAGGAAACCGGACGGAGCAATTTATACTGTCGG
ATCTCCTATTGATTACGGTGTGATCGTGGACACTAAAGCTTATAGCGGAGGTTAT
AATCTGCCAATTGGCCAAGCAGATGAAATGCAACGATATGTCGAAGAAAATCAA
ACACGAAACAAACATATCAACCCTAATGAATGGTGGAAAGTCTATCCATCTTCTG
TAACGGAATTTAAGTTTTTATTTGTGAGTGGTCACTTTAAAGGAAACTACAAAGC
TCAGCTTACACGATTAAATCATATCACTAATTGTAATGGAGCTGTTCTTAGTGTA
GAAGAGCTTTTAATTGGTGGAGAAATGATTAAAGCCGGCACATTAACCTTAGAG
GAAGTCAGACGGAAATTTAATAACGGCGAGATAAACTTTAGATCTTAAgagatcgac
ggtatcgagcggccgctcgataagcttgatatcgaattccgcccctctccctcccccccccctaacgttactggccgaa
gccgcttggaataaggccggtgtgcgtttgtctatatgttattttccaccatattgccgtcttttggcaatgtgagggcccgg
aaacctggccctgtcttcttgacgagcattcctaggggtctttcccctctcgccaaaggaatgcaaggtctgttgaatgtc
gtgaaggaagcagttcctctggaagcttcttgaagacaaacaacgtctgtagcgaccctttgcaggcagcggaaccc
cccacctggcgacaggtgcctctgcggccaaaagccacgtgtataagatacacctgcaaaggcggcacaacccca
gtgccacgttgtgagttggatagttgtggaaagagtcaaatggctctcctcaagcgtattcaacaaggggctgaaggat
gcccagaaggtaccccattgtatgggatctgatctggggcctcggtgcacatgctttacatgtgtttagtcgaggttaaaa
aacgtctaggccccccgaaccacggggacgtggttttcctttgaaaaacacgatgataatatggccacaaccatgac
cgagtacaagcccacggtgcgcctcgccacccgcgacgacgtccccagggccgtacgcaccctcgccgccgcgtt
cgccgactaccccgccacgcgccacaccgtcgatccggaccgccacatcgagcgggtcaccgagctgcaagaac
tcttcctcacgcgcgtcgggctcgacatcggcaaggtgtgggtcgcggacgacggcgccgcggtggcggtctggac
cacgccggagagcgtcgaagcgggggcggtgttcgccgagatcggcccgcgcatggccgagttgagcggttcccg
gctggccgcgcagcaacagatggaaggcctcctggcgccgcaccggcccaaggagcccgcgtggttcctggcca
ccgtcggcgtctcgcccgaccaccagggcaagggtctgggcagcgccgtcgtgctccccggagtggaggcggccg
agcgcgccggggtgcccgccttcctggagacctccgcgccccgcaacctccccttctacgagcggctcggcttcacc
gtcaccgccgacgtcgaggtgcccgaaggaccgcgcacctggtgcatgacccgcaagcccggtgcctgacgccc
gccccacgacccgcagcgcccgaccgaaaggagcgcacgaccccatgcatcgatgatctagagctcgctgatca
gcctcgactgtgccttctagttgccagccatctgttgtttgcccctcccccgtgccttccttgaccctggaaggtgccactcc

cactgtcctttcctaataaaatgaggaaattgcatcgcattgtctgagtaggtgtcattctattctggggggtggggtgggg
caggacagcaagggggaggattgggaagacaatagcaggcatgctggggatgcggtgggctctatggcttctgag
gcggaaagaacctgcagcccaagcttggcgtaatcatggtcatagctgtttcctgtgtgaaattgttatccgctcacaatt
ccacacaacatacgagccggaagcataaagtgtaaagcctggggtgcctaatgagtgagctaactcacattaattgc
gttgcgctcactgcccgctttccagtcgggaaacctgtcgtgccagcggatccgcatctcaattagtcagcaaccatag
tcccgcccctaactccgcccatcccgcccctaactccgcccagttccgcccattctccgccccatggctgactaattttttt
tatttatgcagaggccgaggccgcctcggcctctgagctattccagaagtagtgaggaggcttttttggaggcctaggct
tttgcaaaaagctaacttgtttattgcagcttataatggttacaaataaagcaatagcatcacaaatttcacaaataaagc
atttttttcactgcattctagttgtggtttgtccaaactcatcaatgtatcttatcatgtctggatccgctgcattaatgaatcggc
caacgcgcggggagaggcggtttgcgtattgggcgctcttccgcttcctcgctcactgactcgctgcgctcggtcgttcg
gctgcggcgagcggtatcagctcactcaaaggcggtaatacggttatccacagaatcaggggataacgcaggaaa
gaacatgtgagcaaaaggccagcaaaaggccaggaaccgtaaaaaggccgcgttgctggcgtttttccataggctc
cgcccccctgacgagcatcacaaaaatcgacgctcaagtcagaggtggcgaaacccgacaggactataaagata
ccaggcgtttccccctggaagctccctcgtgcgctctcctgttccgaccctgccgcttaccgcatacctgtccgcctttctc
ccttcgggaagcgtggcgctttctcaatgctcacgctgtaggtatctcagttcggtgtaggtcgttcgctccaagctgggct
gtgtgcacgaaccccccgttcagcccgaccgctgcgccttatccggtaactatcgtcttgagtccaacccggtaagac
acgacttatcgccactggcagcagccactggtaacaggattagcagagcgaggtatgtaggcggtgctacagagttc
ttgaagtggtggcctaactacggctacactagaaggacagtatttggtatctgcgctctgctgaagccagttaccttcgg
aaaaagagttggtagctcttgatccggcaaacaaaccaccgctggtagcggtggtttttttgtttgcaagcagcagatta
cgcgcagaaaaaaaggatctcaagaagatcctttgatcttttctacggggtctgacgctcagtggaacgaaaactcac
gttaagggattttggtcatgagattatcaaaaaggatcttcacctagatccttttaaattaaaaatgaagttttaaatcaatc
taaagtatatatgagtaaacttggtctgacagttaccaatgcttaatcagtgaggcacctatctcagcgatctgtctatttc
gttcatccatagttgcctgactccccgtcgtgtagataactacgatacgggagggcttaccatctggccccagtgctgca
atgataccgcgagacccacgctcaccggctccagatttatcagcaataaaccagccagccggaagggccgagcg
cagaagtggtcctgcaactttatccgcctccatccagtctattaattgttgccgggaagctagagtaagtagttcgccagt
taatagtttgcgcaacgttgttgccattgctacaggcatcgtggtgtcacgctcgtcgtttggtatggcttcattcagctccg
gttcccaacgatcaaggcgagttacatgatcccccatgttgtgcaaaaaagcggttagctccttcggtcctccgatcgtt
gtcagaagtaagttggccgcagtgttatcactcatggttatggcagcactgcataattctcttactgtcatgccatccgtaa
gatgcttttctgtgactggtgagtactcaaccaagtcattctgagaatagtgtatgcggcgaccgagttgctcttgcccgg
cgtcaatacgggataataccgcgccacatagcagaactttaaaagtgctcatcattggaaaacgttcttcggggcgaa
aactctcaaggatcttaccgctgttgagatccagttcgatgtaacccactcgtgcacccaactgatcttcagcatcttttac
tttcaccagcgtttctgggtgagcaaaaacaggaaggcaaaatgccgcaaaaaagggaataagggcgacacgga
aatgttgaatactcatactcttcctttttcaatgaccgctttggaaaaacaaagactgtatttcctggaaattaatgtttattca
ataaactgtgtattcagctatatcacatagtggtgaggctgaaatgaggcgggaagaggcggttggggcttaattatat
caatttgggtggccccacagcgcctccaaggcgccagtcctgttttgacaagttgcctctggaagcctctacaatgcctc
tcttctttttctccagagtaagcggaggccaggggcccccggcctctgcttaatactaaaaaaaacagctgttgtcatag
taatgattgggtggaaacattccaggcctgggtggagaggctttttgcttcctcttgcaaaaccacactgacattccagg
cctgggtggagaggctttttgcttcctcttgcaaaaccacactgccctctggagggcagttgcctagcaactaactaaaa
gaggatgtcgcacggccagctgcggtcagttagtcacttcctgcttaactgacttgacattttctattttaagagtcgggag
gaaaattactgtgttggaggccctccgccatcttctgaagctgaatcgaattaacttgtttattgcagcttataatggttaca
aataaagcaatagcatcacaaatttcacaaataaagcatttttttcactgcattctagttgtggtttgtccaaactcatcaat
gtatcttatcatgtctggatctgatatcatcgtc

N-TALE domain sequence:

ATGGACTACAAAGACCATGACGGTGATTATAAAGATCATGACATCGATTACAAG
GATGACGATGACAAGATGGCCCCCAAGAAGAAGAGGAAGGTGGGCATTCACCG

CGGGGTACCTATGGTGGACTTGAGGACACTCGGTTATTCGCAACAGCAACAGG
AGAAAATCAAGCCTAAGGTCAGGAGCACCGTCGCGCAACACCACGAGGCGCTT
GTGGGGCATGGCTTCACTCATGCGCATATTGTCGCGCTTTCACAGCACCCTGC
GGCGCTTGGGACGGTGGCTGTCAAATACCAAGATATGATTGCGGCCCTGCCCG
AAGCCACGCACGAGGCAATTGTAGGGGTCGGTAAACAGTGGTCGGGAGCGCG
AGCACTTGAGGCGCTGCTGACTGTGGCGGGTGAGCTTAGGGGGCCTCCGCTC
CAGCTCGACACCGGGCAGCTGCTGAAGATCGCGAAGAGAGGGGGAGTAACAG
CGGTAGAGGCAGTGCACGCCTGGCGCAATGCGCTCACCGGGGCCCCCTTGAA
C

BciVI stuffer:

CTGCAGGATACGTTTATCAGTGATAAAGTGTCAAGCATGACAAAGTTGCAGCC
GAATACAGTGGTATCCTGCAG

3d16 overlap:

GTGCTCTGTCAGGACCACGGG

16.5 half-repeat sequence:

CTTACGCCACAACAGGTGGTGGCGATTGCATCTAATGGAGGCGGACGCCCTGC
CTTGGAG

C-TALE domain:

TCCATCGTAGCCCAATTGTCCAGGCCCGATCCCGCGTTGGCTGCGTTAACGAAT
GACCATCTGGTGGCGTTGGCATGTCTTGGTGGACGACCCGCGCTCGATGCAGT
CAAAAAGGGTCTGCCTCATGCTCCCGCATTGATCAAAAGAACCAACCGGCGGAT
TCCCGAGAGAACTTCCCATCGAGTCGCG

FokI endonuclease domain:

CAACTAGTCAAAAGTGAACTGGAGGAGAAGAAATCTGAACTTCGTCATAAATTG
AAATATGTGCCTCATGAATATATTGAATTAATTGAAATTGCCAGAAATTCCACTCA
GGATAGAATTCTTGAAATGAAGGTAATGGAATTTTTTATGAAAGTTTATGGATATA
GAGGTAAACATTTGGGTGGATCAAGGAAACCGGACGGAGCAATTTATACTGTCG
GATCTCCTATTGATTACGGTGTGATCGTGGACACTAAAGCTTATAGCGGAGGTT
ATAATCTGCCAATTGGCCAAGCAGATGAAATGCAACGATATGTCGAAGAAAATC
AAACACGAAACAAACATATCAACCCTAATGAATGGTGGAAAGTCTATCCATCTTC
TGTAACGGAATTTAAGTTTTTATTTGTGAGTGGTCACTTTAAAGGAAACTACAAA
GCTCAGCTTACACGATTAAATCATATCACTAATTGTAATGGAGCTGTTCTTAGTG
TAGAAGAGCTTTTAATTGGTGGAGAAATGATTAAAGCCGGCACATTAACCTTAGA
GGAAGTCAGACGGAAATTTAATAACGGCGAGATAAACTTT

All four targeting vectors differ only in the 16.5 half repeat as this includes another RVD targeting the 17th nucleotide in a 17bp target sequence.
The 16.5 half repeat is double underlined in the pTALEN-STAR-T sequence above and has to be exchanged for the following sequences to generate the other destination vectors pTALEN-STAR-A, pTALEN-STAR-C and pTALEN-STAR-G.

- pTALEN-STAR-A – 16.5 half repeat – RVD sequence shown in bold
  CTTACGCCACAACAGGTGGTGGCGATTGCATCT**AACATT**GGCGGACGC
  CCTGCCTTGGTG
- pTALEN-STAR-C – 16.5 half repeat – RVD sequence shown in bold
  CTTACGCCACAACAGGTGGTGGCGATTGCATCT**CACGAC**GGCGGACGC
  CCTGCCTTGGTG
- pTALEN-STAR-G – 16.5 half repeat – RVD sequence shown in bold
  CTTACGCCACAACAGGTGGTGGCGATTGCATCT**AATCAC**GGCGGACGC
  CCTGCCTTGGTG

Supplementary File S2. Sequences of STAR TALE TF destination vectors and component parts.

Sequences of complete STAR vectors and component parts

Below is the sequence for a STAR vector for TALE TF assembly coding for a VP64 domain.

General features and positions:

Feature	Position	Length [bp]
N TALE	1737..2264	528
3xFLAG	1740..1805	66
SV40 NLS	1812..1832	21
BciVI stuffer	2265..2338	74
3d16 Gibson overlap	2339..2359	21
16.5 half repeat	2360..2419	60
C TALE	2420..2608	189
VP64	2660..2809	150
T2A	2831..2884	54
AmpR	5346..6206	861

pTALE-STARVP64-T (NG) plasmid – a corresponding plasmid map is shown in Suppl. Fig. S9:

gacattgattattgactagttattaatagtaatcaattacggggtcattagttcatagcccatatatggagttccgcgttacat
aacttacggtaaatggcccgcctggctgaccgcccaacgacccccgcccattgacgtcaataatgacgtatgttccca
tagtaacgccaatagggactttccattgacgtcaatgggtggactatttacggtaaactgcccacttggcagtacatcaa
gtgtatcatatgccaagtacgccccctattgacgtcaatgacggtaaatggcccgcctggcattatgcccagtacatga
ccttatgggactttcctacttggcagtacatctacgtattagtcatcgctattaccatgggtcgaggtgagccccacgttctg
cttcactctccccatctcccccccctccccacccccaattttgtatttatttattttttaattattttgtgcagcgatgggggcgg
gggggggggggcgcgcgccaggcggggcggggcggggcgaggggcggggcggggcgaggcggagaggt
gcggcggcagccaatcagagcggcgcgctccgaaagtttccttttatggcgaggcggcggcggcggcggccctata
aaaagcgaagcgcgcggcgggcgggagtcgctgcgttgccttcgccccgtgccccgctccgcgccgcctcgcgcc
gcccgccccggctctgactgaccgcgttactcccacaggtgagcgggcgggacggcccttctcctccgggctgtaatt
agcgcttggtttaatgacggctcgtttcttttctgtggctgcgtgaaagccttaaagggctccgggagggccctttgtgcgg
gggggagcggctcggggggtgcgtgcgtgtgtgtgtgcgtggggagcgccgcgtgcggcccgcgctgcccggcgg
ctgtgagcgctgcgggcgcggcgcggggctttgtgcgctccgcgtgtgcgcgaggggagcgcggccgggggcggt
gccccgcggtgcggggggggctgcgaggggaacaaaggctgcgtgcggggtgtgtgcgtgggggggtgagcagg
gggtgtgggcgcggcggtcgggctgtaacccccccctgcacccccctccccgagttgctgagcacggcccggcttcg
ggtgcggggctccgtgcggggcgtggcgcggggctcgccgtgccgggcggggggtggcggcaggtgggggtgcc

gggcggggcggggccgcctcgggccggggagggctcgggggaggggcgcggcggccccggagcgccggcgg
ctgtcgaggcgcggcgagccgcagccattgccttttatggtaatcgtgcgagagggcgcagggacttcctttgtcccaa
atctggcggagccgaaatctgggaggcgccgccgcacccccctctagcgggcgcgggcgaagcggtgcggcgcc
ggcaggaaggaaatgggcggggagggccttcgtgcgtcgccgcgccgccgtccccttctccatctccagcctcggg
gctgccgcaggggggacggctgccttcgggggggacggggcagggcggggttcggcttctggcgtgtgaccggcgg
ctctagagcctctgctaaccatgttcatgccttcttctttttcctacagctcctgggcaacgtgctggttgttgtgctgtctcatc
attttggcaaagaattcctcgaggccgccaccATGGACTACAAAGACCATGACGGTGATTATAA
AGATCATGACATCGATTACAAGGATGACGATGACAAGATGGCCCCCAAGAAGAA
GAGGAAGGTGGGCATTCACCGCGGGGTACCTATGGTGGACTTGAGGACACTCG
GTTATTCGCAACAGCAACAGGAGAAAATCAAGCCTAAGGTCAGGAGCACCGTC
GCGCAACACCACGAGGCGCTTGTGGGGCATGGCTTCACTCATGCGCATATTGT
CGCGCTTTCACAGCACCCTGCGGCGCTTGGGACGGTGGCTGTCAAATACCAAG
ATATGATTGCGGCCCTGCCCGAAGCCACGCACGAGGCAATTGTAGGGGTCGGT
AAACAGTGGTCGGGAGCGCGAGCACTTGAGGCGCTGCTGACTGTGGCGGGTG
AGCTTAGGGGGCCTCCGCTCCAGCTCGACACCGGGCAGCTGCTGAAGATCGC
GAAGAGAGGGGGAGTAACAGCGGTAGAGGCAGTGCACGCCTGGCGCAATGCG
CTCACCGGGGCCCCCTTGAAC**CTGCAGGATACGTTTATCAGTGATAAAGTGTC**
AAGCATGACAAAGTTGCAGCCGAATACAGTGGTATCCTGCAGGTGCTCTGTCA
GGACCACGGGCTTACGCCACAACAGGTGGTGGCGATTGCATCTAATGGAGGCG
GACGCCCTGCCTTGGAG*TCCATCGTAGCCCAATTGTCCAGGCCCGATCCCGCG*
TTGGCTGCGTTAACGAATGACCATCTGGTGGCGTTGGCATGTCTTGGTGGACG
ACCCGCGCTCGATGCAGTCAAAAAGGGTCTGCCTCATGCTCCCGCATTGATCAA
*AAGAACCAACCGGCGGATTCCCGAGAGAACTTCCCATCGAGTCGCG*GCTAGCC
CCAAGAAGAAGAGAAAGGTGGAGGCCAGCGGTTCCGGACGGGCTGACGCATT
GGACGATTTTGATCTGGATATGCTGGGAAGTGACGCCCTCGATGATTTTGACCT
TGACATGCTTGGTTCGGATGCCCTTGATGACTTTGACCTCGACATGCTCGGCAG
TGACGCCCTTGATGATTTCGACCTGGACATGCTGATTAACTCTAGAGGCAGTGG
AGAGGGCAGAGGAAGTCTGCTAACATGCGGTGACGTCGAGGAGAATCCTGGCC
CAACCGAGTACAAGCCCACGGTGCGCCTCGCCACCCGCGACGAacgtccccagggc
cgtacgcaccctcgccgccgcgttcgccgactaccccgccacgcgccacaccgtcgatccggaccgccacatcga
gcgggtcaccgagctgcaagaactcttcctcacgcgcgtcgggctcgacatcggcaaggtgtgggtcgcggacgac
ggcgccgcggtggcggtctggaccacgccggagagcgtcgaagcgggggcggtgttcgccgagatcggcccgcg
catggccgagttgagcggttcccggctggccgcgcagcaacagatggaaggcctcctggcgccgcaccggcccaa
ggagcccgcgtggttcctggccaccgtcggcgtctcgcccgaccaccagggcaagggtctgggcagcgccgtcgtg
ctccccggagtggaggcggccgagcgcgccggggtgcccgccttcctggagacctccgcgccccgcaacctcccc
ttctacgagcggctcggcttcaccgtcaccgccgacgtcgaggtgcccgaaggaccgcgcacctggtgcatgaccc
gcaagcccggtgcctgacgcccgccccacgacccgcagcgcccgaccgaaaggagcgcacgaccccatgcatc

gatgatctagagctcgctgatcagcctcgactgtgccttctagttgccagccatctgttgtttgcccctcccccgtgccttcc
ttgaccctggaaggtgccactcccactgtcctttcctaataaaatgaggaaattgcatcgcattgtctgagtaggtgtcatt
ctattctggggggtggggtggggcaggacagcaagggggaggattgggaagacaatagcaggcatgctggggat
gcggtgggctctatggcttctgaggcggaaagaacctgcagcccaagcttggcgtaatcatggtcatagctgtttcctgt
gtgaaattgttatccgctcacaattccacacaacatacgagccggaagcataaagtgtaaagcctggggtgcctaatg
agtgagctaactcacattaattgcgttgcgctcactgcccgctttccagtcgggaaacctgtcgtgccagcggatccgc
atctcaattagtcagcaaccatagtcccgcccctaactccgcccatcccgcccctaactccgcccagttccgcccattct
ccgccccatggctgactaattttttttatttatgcagaggccgaggccgcctcggcctctgagctattccagaagtagtga
ggaggcttttttggaggcctaggcttttgcaaaaagctaacttgtttattgcagcttataatggttacaaataaagcaatag
catcacaaatttcacaaataaagcatttttttcactgcattctagttgtggtttgtccaaactcatcaatgtatcttatcatgtct
ggatccgctgcattaatgaatcggccaacgcgcggggagaggcggtttgcgtattgggcgctcttccgcttcctcgctc
actgactcgctgcgctcggtcgttcggctgcggcgagcggtatcagctcactcaaaggcggtaatacggttatccaca
gaatcaggggataacgcaggaaagaacatgtgagcaaaaggccagcaaaaggccaggaaccgtaaaaaggc
cgcgttgctggcgtttttccataggctccgcccccctgacgagcatcacaaaaatcgacgctcaagtcagaggtggcg
aaacccgacaggactataaagataccaggcgtttccccctggaagctccctcgtgcgctctcctgttccgaccctgcc
gcttaccgcatacctgtccgcctttctcccttcgggaagcgtggcgctttctcaatgctcacgctgtaggtatctcagttcgg
tgtaggtcgttcgctccaagctgggctgtgtgcacgaaccccccgttcagcccgaccgctgcgccttatccggtaactat
cgtcttgagtccaacccggtaagacacgacttatcgccactggcagcagccactggtaacaggattagcagagcga
ggtatgtaggcggtgctacagagttcttgaagtggtggcctaactacggctacactagaaggacagtatttggtatctgc
gctctgctgaagccagttaccttcggaaaaagagttggtagctcttgatccggcaaacaaaccaccgctggtagcggt
ggtttttttgtttgcaagcagcagattacgcgcagaaaaaaaggatctcaagaagatcctttgatcttttctacggggtctg
acgctcagtggaacgaaaactcacgttaagggattttggtcatgagattatcaaaaaggatcttcacctagatccttttа
aattaaaaatgaagttttaaatcaatctaaagtatatatgagtaaacttggtctgacagttaccaatgcttaatcagtgag
gcacctatctcagcgatctgtctatttcgttcatccatagttgcctgactccccgtcgtgtagataactacgatacgggagg
gcttaccatctggccccagtgctgcaatgataccgcgagacccacgctcaccggctccagatttatcagcaataaacc
agccagccggaagggccgagcgcagaagtggtcctgcaactttatccgcctccatccagtctattaattgttgccggg
aagctagagtaagtagttcgccagttaatagtttgcgcaacgttgttgccattgctacaggcatcgtggtgtcacgctcgt
cgtttggtatggcttcattcagctccggttcccaacgatcaaggcgagttacatgatcccccatgttgtgcaaaaaagcg
gttagctccttcggtcctccgatcgttgtcagaagtaagttggccgcagtgttatcactcatggttatggcagcactgcata
attctcttactgtcatgccatccgtaagatgcttttctgtgactggtgagtactcaaccaagtcattctgagaatagtgtatgc
ggcgaccgagttgctcttgcccggcgtcaatacgggataataccgcgccacatagcagaactttaaaagtgctcatca
ttggaaaacgttcttcggggcgaaaactctcaaggatcttaccgctgttgagatccagttcgatgtaacccactcgtgca
cccaactgatcttcagcatcttttactttcaccagcgtttctgggtgagcaaaaacaggaaggcaaaatgccgcaaaa
aagggaataagggcgacacggaaatgttgaatactcatactcttcctttttcaatgaccgctttggaaaaacaaagact
gtatttcctggaaattaatgtttattcaataaactgtgtattcagctatatcacatagtggtgaggctgaaatgaggcgga
agaggcggttggggcttaattatatcaatttgggtggccccacagcgcctccaaggcgccagtcctgttttgacaagttg
cctctggaagcctctacaatgcctctcttctttttctccagagtaagcggaggccaggggcccccggcctctgcttaatac

taaaaaaaacagctgttgtcatagtaatgattgggtggaaacattccaggcctgggtggagaggctttttgcttcctcttg caaaaccacactgacattccaggcctgggtggagaggctttttgcttcctcttgcaaaaccacactgccctctggaggg cagttgcctagcaactaactaaaagaggatgtcgcacggccagctgcggtcagttagtcacttcctgcttaactgactt gacattttctattttaagagtcgggaggaaaattactgtgttggaggccctccgccatcttctgaagctgaatcgaattaa cttgtttattgcagcttataatggttacaaataaagcaatagcatcacaaatttcacaaataaagcatttttttcactgcattc tagttgtggtttgtccaaactcatcaatgtatcttatcatgtctggatctgatatcatcgtc

The half repeat sequence is double underlined.

N-TALE domain sequence:

ATGGACTACAAAGACCATGACGGTGATTATAAAGATCATGACATCGATTACAAG
GATGACGATGACAAGATGGCCCCCAAGAAGAAGAGGAAGGTGGGCATTCACCG
CGGGGTACCTATGGTGGACTTGAGGACACTCGGTTATTCGCAACAGCAACAGG
AGAAAATCAAGCCTAAGGTCAGGAGCACCGTCGCGCAACACCACGAGGCGCTT
GTGGGGCATGGCTTCACTCATGCGCATATTGTCGCGCTTTCACAGCACCCTGC
GGCGCTTGGGACGGTGGCTGTCAAATACCAAGATATGATTGCGGCCCTGCCCG
AAGCCACGCACGAGGCAATTGTAGGGGTCGGTAAACAGTGGTCGGGAGCGCG
AGCACTTGAGGCGCTGCTGACTGTGGCGGGTGAGCTTAGGGGGCCTCCGCTC
CAGCTCGACACCGGGCAGCTGCTGAAGATCGCGAAGAGAGGGGGAGTAACAG
CGGTAGAGGCAGTGCACGCCTGGCGCAATGCGCTCACCGGGGCCCCCTTGAA
C

BciVI stuffer:

CTGCAGGATACGTTTATCAGTGATAAAGTGTCAAGCATGACAAAGTTGCAGCC GAATACAGTGGTATCCTGCAG

3d16 overlap:

GTGCTCTGTCAGGACCACGGG

16.5 half-repeat sequence:

CTTACGCCACAACAGGTGGTGGCGATTGCATCTAATGGAGGCGGACGCCCTGC CTTGGAG

C-TALE domain:

TCCATCGTAGCCCAATTGTCCAGGCCCGATCCCGCGTTGGCTGCGTTAACGAAT GACCATCTGGTGGCGTTGGCATGTCTTGGTGGACGACCCGCGCTCGATGCAGT CAAAAAGGGTCTGCCTCATGCTCCCGCATTGATCAAAAGAACCAACCGGCGGAT TCCCGAGAGAACTTCCCATCGAGTCGCG

VP64 domain:

GACGCATTGGACGATTTTGATCTGGATATGCTGGGAAGTGACGCCCTCGATGAT
TTTGACCTTGACATGCTTGGTTCGGATGCCCTTGATGACTTTGACCTCGACATG
CTCGGCAGTGACGCCCTTGATGATTTCGACCTGGACATGCTG

All four targeting vectors differ only in the 16.5 half repeat as this includes another RVD targeting the 17th nucleotide in a 17bp target sequence.

The 16.5 half repeat is double underlined in the pTALE-STARVP64-T sequence above and has to be exchanged for the following sequences to generate the other destination vectors pTALE-STARVP64-A, pTALE-STARVP64-C and pTALE-STARVP64-G.

- pTALE-STARVP64-A – 16.5 half repeat – RVD sequence shown in bold
  CTTACGCCACAACAGGTGGTGGCGATTGCATCT**AACATT**GGCGGACGC
  CCTGCCTTGGTG
- pTALE-STARVP64-C – 16.5 half repeat – RVD sequence shown in bold
  CTTACGCCACAACAGGTGGTGGCGATTGCATCT**CACGAC**GGCGGACGC
  CCTGCCTTGGTG
- pTALE-STARVP64-G – 16.5 half repeat – RVD sequence shown in bold
  CTTACGCCACAACAGGTGGTGGCGATTGCATCT**AATCAC**GGCGGACGC
  CCTGCCTTGGTG

Supplementary File S3. Sequence of STAR RFP entry destination vectors and component parts.

Sequences of complete STAR vectors and component parts

Below is the sequence for a STAR RFP entry vector for easy exchange of functional domains in TALE assembly.

General features and positions:

Feature	Position	Length [bp]
N TALE	1737..2264	528
3xFLAG	1740..1805	66
SV40 NLS	1812..1832	21
BciVI stuffer	2265..2338	74
3d16 Gibson overlap	2339..2359	21
16.5 half repeat	2360..2419	60
C TALE	2420..2608	189
RFP cassette	2671..3596	904
5’ BsmBI	2659..2664	6
3’ BsmBI	3789..3794	6
AmpR	5598..6458	861

pTALE-STAR-T_RFP entry (NG) plasmid – a corresponding plasmid map is shown in Suppl. Fig. S10:

gacattgattattgactagttattaatagtaatcaattacggggtcattagttcatagcccatatatggagttccgcgttacat
aacttacggtaaatggcccgcctggctgaccgcccaacgacccccgcccattgacgtcaataatgacgtatgttccca
tagtaacgccaatagggactttccattgacgtcaatgggtggactatttacggtaaactgcccacttggcagtacatcaa
gtgtatcatatgccaagtacgccccctattgacgtcaatgacggtaaatggcccgcctggcattatgcccagtacatga
ccttatgggactttcctacttggcagtacatctacgtattagtcatcgctattaccatgggtcgaggtgagccccacgttctg
cttcactctccccatctcccccccctccccacccccaattttgtatttatttattttttaattattttgtgcagcgatgggggcgg
ggggggggggggcgcgcgccaggcggggcggggcggggcgaggggcggggcggggcgaggcggagaggt
gcggcggcagccaatcagagcggcgcgctccgaaagtttccttttatggcgaggcggcggcggcggcggccctata
aaaagcgaagcgcgcggcgggcgggagtcgctgcgttgccttcgccccgtgccccgctccgcgccgcctcgcgcc
gcccgccccggctctgactgaccgcgttactcccacaggtgagcgggcgggacggcccttctcctccgggctgtaatt
agcgcttggtttaatgacggctcgtttcttttctgtggctgcgtgaaagccttaaagggctccgggagggccctttgtgcgg
gggggagcggctcggggggtgcgtgcgtgtgtgtgtgcgtggggagcgccgcgtgcggcccgcgctgcccggcgg
ctgtgagcgctgcgggcgcggcgcggggctttgtgcgctccgcgtgtgcgcgaggggagcgcggccgggggcggt
gccccgcggtgcggggggggctgcgaggggaacaaaggctgcgtgcggggtgtgtgcgtgggggggtgagcagg
gggtgtgggcgcggcggtcgggctgtaacccccccctgcacccccctccccgagttgctgagcacggcccggcttcg
ggtgcggggctccgtgcggggcgtggcgcggggctcgccgtgccgggcggggggtggcggcaggtgggggtgcc
gggcggggcggggccgcctcgggccggggagggctcgggggagggggcgcggcggcccccggagcgccggcgg
ctgtcgaggcgcggcgagccgcagccattgccttttatggtaatcgtgcgagagggcgcagggacttcctttgtcccaa
atctggcggagccgaaatctgggaggcgccgccgcacccctctagcgggcgcgggcgaagcggtgcggcgcc
ggcaggaaggaaatgggcggggagggccttcgtgcgtcgccgcgccgccgtccccttctccatctccagcctcggg

gctgccgcagggggacggctgccttcgggggggacggggcagggcggggttcggcttctggcgtgtgaccggcgg
ctctagagcctctgctaaccatgttcatgccttcttctttttcctacagctcctgggcaacgtgctggttgttgtgctgtctcatc
attttggcaaagaattcctcgaggccgccaccATGGACTACAAAGACCATGACGGTGATTATAA
AGATCATGACATCGATTACAAGGATGACGATGACAAGATGGCCCCCAAGAAGAA
GAGGAAGGTGGGCATTCACCGCGGGGTACCTATGGTGGACTTGAGGACACTCG
GTTATTCGCAACAGCAACAGGAGAAAATCAAGCCTAAGGTCAGGAGCACCGTC
GCGCAACACCACGAGGCGCTTGTGGGGCATGGCTTCACTCATGCGCATATTGT
CGCGCTTTCACAGCACCCTGCGGCGCTTGGGACGGTGGCTGTCAAATACCAAG
ATATGATTGCGGCCCTGCCCGAAGCCACGCACGAGGCAATTGTAGGGGTCGGT
AAACAGTGGTCGGGAGCGCGAGCACTTGAGGCGCTGCTGACTGTGGCGGGTG
AGCTTAGGGGGCCTCCGCTCCAGCTCGACACCGGGCAGCTGCTGAAGATCGC
GAAGAGAGGGGGAGTAACAGCGGTAGAGGCAGTGCACGCCTGGCGCAATGCG
CTCACCGGGGCCCCCTTGAAC**CTGCAGGATACGTTTATCAGTGATAAAGTGTC**
AAGCATGACAAAGTTGCAGCCGAATACAGTGGTATCCTGCAGGTGCTCTGTCA
GGACCACGGGCTTACGCCACAACAGGTGGTGGCGATTGCATCTAATGGAGGCG
GACGCCCTGCCTTGGAG*TCCATCGTAGCCCAATTGTCCAGGCCCGATCCCGCG*
TTGGCTGCGTTAACGAATGACCATCTGGTGGCGTTGGCATGTCTTGGTGGACG
ACCCGCGCTCGATGCAGTCAAAAAGGGTCTGCCTCATGCTCCCGCATTGATCAA
*AAGAACCAACCGGCGGATTCCCGAGAGAACTTCCCATCGAGTCGCG*GCTAGCC
CCAAGAAGAAGAGAAAGGTGGAGGCCAGCGGTTCCGGAcagtagagacgccattggaat
tcgcggccgcttctagag**caatacgcaaaccgcctctccccgcgcgttggccgattcattaatgcagctggca**
cgacaggtttcccgactggaaagcgggcagtgagcgcaacgcaattaatgtgagttagctcactcattagg
caccccaggctttacactttatgcttccggctcgtatgttgtgtggaattgtgagcggataacaatttcacacat
actagagaaagaggagaaatactagatggcttcctccgaagacgttatcaaagagttcatgcgtttcaaagtt
cgtatggaaggttccgttaacggtcacgagttcgaaatcgaaggtgaaggtgaaggtcgtccgtacgaagg
tacccagaccgctaaactgaaagttaccaaaggtggtccgctgccgttcgcttgggacatcctgtccccgca
gttccagtacggttccaaagcttacgttaaacacccggctgacatcccggactacctgaaactgtccttcccg
gaaggtttcaaatgggaacgtgttatgaacttcgaagacggtggtgttgttaccgttacccaggactcctccct
gcaagacggtgagttcatctacaaagttaaactgcgtggtaccaacttcccgtccgacggtccggttatgca
gaaaaaaaccatgggttgggaagcttccaccgaacgtatgtacccggaagacggtgctctgaaaggtgaa
atcaaaatgcgtctgaaactgaaagacggtggtcactacgacgctgaagttaaaaccacctacatggctaa
aaaaccggttcagctgccgggtgcttacaaaaccgacatcaaactggacatcacctcccacaacgaagac
tacaccatcgttgaacagtacgaacgtgctgaaggtcgtcactccaccggtgcttaataacgctgatagtgcta
gtgtagatcgctactagagccaggcatcaaataaaacgaaaggctcagtcgaaagactgggcctttcgttttatctgtt
gtttgtcggtgaacgctctctactagagtcacactggctcaccttcgggtgggcctttctgcgtttatatactagtagcggcc
gctgcagtcagaccgtctcaagctgagctcgctgatcagcctcgactgtgccttctagttgccagccatctgttgtttgccc
ctcccccgtgccttccttgaccctggaaggtgccactcccactgtcctttcctaataaaatgaggaaattgcatcgcattgt
ctgagtaggtgtcattctattctggggggtggggtggggcaggacagcaagggggaggattgggaagacaatagca
ggcatgctggggatgcggtgggctctatggcttctgaggcggaaagaacctgcagcccaagcttggcgtaatcatggt
catagctgtttcctgtgtgaaattgttatccgctcacaattccacacaacatacgagccggaagcataaagtgtaaagc
ctggggtgcctaatgagtgagctaactcacattaattgcgttgcgctcactgcccgctttccagtcgggaaacctgtcgtg
ccagcggatccgcatctcaattagtcagcaaccatagtcccgcccctaactccgcccatcccgcccctaactccgccc
agttccgcccattctccgccccatggctgactaattttttttatttatgcagaggccgaggccgcctcggcctctgagctatt
ccagaagtagtgaggaggcttttttggaggcctaggcttttgcaaaaagctaacttgtttattgcagcttataatggttaca
aataaagcaatagcatcacaaatttcacaaataaagcatttttttcactgcattctagttgtggtttgtccaaactcatcaat
gtatcttatcatgtctggatccgctgcattaatgaatcggccaacgcgcggggagaggcggtttgcgtattgggcgctctt
ccgcttcctcgctcactgactcgctgcgctcggtcgttcggctgcggcgagcggtatcagctcactcaaaggcggtaat
acggttatccacagaatcaggggataacgcaggaaagaacatgtgagcaaaaggccagcaaaaggccaggaa

ccgtaaaaaggccgcgttgctggcgtttttccataggctccgcccccctgacgagcatcacaaaaatcgacgctcaa
gtcagaggtggcgaaacccgacaggactataaagataccaggcgtttccccctggaagctccctcgtgcgctctcct
gttccgaccctgccgcttaccgcatacctgtccgcctttctcccttcgggaagcgtggcgctttctcaatgctcacgctgta
ggtatctcagttcggtgtaggtcgttcgctccaagctgggctgtgtgcacgaaccccccgttcagcccgaccgctgcgc
cttatccggtaactatcgtcttgagtccaacccggtaagacacgacttatcgccactggcagcagccactggtaacag
gattagcagagcgaggtatgtaggcggtgctacagagttcttgaagtggtggcctaactacggctacactagaagga
cagtatttggtatctgcgctctgctgaagccagttaccttcggaaaaagagttggtagctcttgatccggcaaacaaacc
accgctggtagcggtggtttttttgtttgcaagcagcagattacgcgcagaaaaaaaggatctcaagaagatcctttgat
cttttctacggggtctgacgctcagtggaacgaaaactcacgttaagggattttggtcatgagattatcaaaaaggatctt
cacctagatccttttaaattaaaaatgaagttttaaatcaatctaaagtatatatgagtaaacttggtctgacagttaccaat
gcttaatcagtgaggcacctatctcagcgatctgtctatttcgttcatccatagttgcctgactccccgtcgtgtagataact
acgatacgggagggcttaccatctggccccagtgctgcaatgataccgcgagacccacgctcaccggctccagattt
atcagcaataaaccagccagccggaagggccgagcgcagaagtggtcctgcaactttatccgcctccatccagtct
attaattgttgccgggaagctagagtaagtagttcgccagttaatagtttgcgcaacgttgttgccattgctacaggcatc
gtggtgtcacgctcgtcgtttggtatggcttcattcagctccggttcccaacgatcaaggcgagttacatgatcccccatgt
tgtgcaaaaaagcggttagctccttcggtcctccgatcgttgtcagaagtaagttggccgcagtgttatcactcatggttat
ggcagcactgcataattctcttactgtcatgccatccgtaagatgcttttctgtgactggtgagtactcaaccaagtcattct
gagaatagtgtatgcggcgaccgagttgctcttgcccggcgtcaatacgggataataccgcgccacatagcagaact
ttaaaagtgctcatcattggaaaacgttcttcggggcgaaaactctcaaggatcttaccgctgttgagatccagttcgatg
taacccactcgtgcacccaactgatcttcagcatcttttactttcaccagcgtttctgggtgagcaaaaacaggaaggca
aaatgccgcaaaaaagggaataagggcgacacggaaatgttgaatactcatactcttcctttttcaatgaccgctttgg
aaaaacaaagactgtatttcctggaaattaatgtttattcaataaactgtgtattcagctatatcacatagtggtgaggctg
aaatgaggcgggaagaggcggttggggcttaattatatcaatttgggtggccccacagcgcctccaaggcgccagtc
ctgttttgacaagttgcctctggaagcctctacaatgcctctcttctttttctccagagtaagcggaggccaggggccccc
ggcctctgcttaatactaaaaaaaacagctgttgtcatagtaatgattgggtggaaacattccaggcctgggtggagag
gctttttgcttcctcttgcaaaaccacactgacattccaggcctgggtggagaggctttttgcttcctcttgcaaaaccaca
ctgccctctggagggcagttgcctagcaactaactaaaagaggatgtcgcacggccagctgcggtcagttagtcactt
cctgcttaactgacttgacattttctattttaagagtcgggaggaaaattactgtgttggaggccctccgccatcttctgaag
ctgaatcgaattaacttgtttattgcagcttataatggttacaaataaagcaatagcatcacaaatttcacaaataaagca
tttttttcactgcattctagttgtggtttgtccaaactcatcaatgtatcttatcatgtctggatctgatatcatcgtc

N-TALE domain sequence:

ATGGACTACAAAGACCATGACGGTGATTATAAAGATCATGACATCGATTACAAG
GATGACGATGACAAGATGGCCCCCAAGAAGAAGAGGAAGGTGGGCATTCACCG
CGGGGTACCTATGGTGGACTTGAGGACACTCGGTTATTCGCAACAGCAACAGG
AGAAAATCAAGCCTAAGGTCAGGAGCACCGTCGCGCAACACCACGAGGCGCTT
GTGGGGCATGGCTTCACTCATGCGCATATTGTCGCGCTTTCACAGCACCCTGC
GGCGCTTGGGACGGTGGCTGTCAAATACCAAGATATGATTGCGGCCCTGCCCG
AAGCCACGCACGAGGCAATTGTAGGGGTCGGTAAACAGTGGTCGGGAGCGCG
AGCACTTGAGGCGCTGCTGACTGTGGCGGGTGAGCTTAGGGGGCCTCCGCTC
CAGCTCGACACCGGGCAGCTGCTGAAGATCGCGAAGAGAGGGGGAGTAACAG
CGGTAGAGGCAGTGCACGCCTGGCGCAATGCGCTCACCGGGGCCCCCTTGAA
C

BciVI stuffer:

**CTGCAGGATACGTTTATCAGTGATAAAGTGTCAAGCATGACAAAGTTGCAGCC
GAATACAGTGGTATCCTGCAG**

3d16 overlap:
GTGCTCTGTCAGGACCACGGG

16.5 half-repeat sequence:
CTTACGCCACAACAGGTGGTGGCGATTGCATCTAATGGAGGCGGACGCCCTGC
CTTGGAG

C-TALE domain:
*TCCATCGTAGCCCAATTGTCCAGGCCCGATCCCGCGTTGGCTGCGTTAACGAAT
GACCATCTGGTGGCGTTGGCATGTCTTGGTGGACGACCCGCGCTCGATGCAGT
CAAAAAGGGTCTGCCTCATGCTCCCGCATTGATCAAAAGAACCAACCGGCGGAT
TCCCGAGAGAACTTCCCATCGAGTCGCG*

RFP cassette:
**caatacgcaaaccgcctctccccgcgcgttggccgattcattaatgcagctggcacgacaggtttcccgact
ggaaagcgggcagtgagcgcaacgcaattaatgtgagttagctcactcattaggcaccccaggctttacac
tttatgcttccggctcgtatgttgtgtggaattgtgagcggataacaatttcacacatactagagaaagaggag
aaatactagatggcttcctccgaagacgttatcaaagagttcatgcgtttcaaagttcgtatggaaggttccgtt
aacggtcacgagttcgaaatcgaaggtgaaggtgaaggtcgtccgtacgaaggtacccagaccgctaaac
tgaaagttaccaaaggtggtccgctgccgttcgcttgggacatcctgtccccgcagttccagtacggttccaa
agcttacgttaaacacccggctgacatcccggactacctgaaactgtccttcccggaaggtttcaaatggga
acgtgttatgaacttcgaagacggtggtgttgttaccgttacccaggactcctccctgcaagacggtgagttc
atctacaaagttaaactgcgtggtaccaacttcccgtccgacggtccggttatgcagaaaaaaaccatgggtt
gggaagcttccaccgaacgtatgtacccggaagacggtgctctgaaaggtgaaatcaaaatgcgtctgaaa
ctgaaagacggtggtcactacgacgctgaagttaaaaccacctacatggctaaaaaaccggttcagctgcc
gggtgcttacaaaaccgacatcaaactggacatcacctcccacaacgaagactacaccatcgttgaacagt
acgaacgtgctgaaggtcgtcactccaccggtgcttaa**

All four RFP entry vectors differ only in the 16.5 half repeat as this includes another RVD targeting the 17th nucleotide in a 17bp target sequence.
The 16.5 half repeat is double underlined in the pTALE-STAR-T_RFP entry sequence above and has to be exchanged for the following sequences to generate the other destination vectors pTALE-STAR-A_RFP entry, pTALE-STAR-C_RFP entry and pTALE-STAR-G_RFP entry.

- pTALE-STAR-A_RFP entry – 16.5 half repeat – RVD sequence shown in bold
  CTTACGCCACAACAGGTGGTGGCGATTGCATCT**AACATT**GGCGGACGC
CCTGCCTTGGTG
- pTALE-STAR-C_RFP entry – 16.5 half repeat – RVD sequence shown in bold
  CTTACGCCACAACAGGTGGTGGCGATTGCATCT**CACGAC**GGCGGACGC
CCTGCCTTGGTG
- pTALEN-STAR-G_RFP entry – 16.5 half repeat – RVD sequence shown in bold
  CTTACGCCACAACAGGTGGTGGCGATTGCATCT**AATCAC**GGCGGACGC
  CCTGCCTTGGTG

Supplementary Methods. Detailed STAR protocol.

Library preparation

Primers used: pTAL3: CGCGACGTAATACGACTCAC; pTAL4: CACGACAGGTTTCCCGACT.

Template input: 1 ng at 1 ng/µl (= 1µl)

Using Herculase Ⅱ DNA Polymerase (Agilent Technologies) two reactions are enough to use the 1mer for 32 assemblies; this protocol is for quadruplicate PCRs.

Master Mix:

	Per reaction [µl]	70x MasterMix [µl]
H_2O	64	4480
Buffer	20	1400
PrimerMix (10µM each)	5	350
dNTPs (10mmM)	10	700
Herculase Ⅱ	1	70
Total	*100*	*7000*

Cycling conditions: 98˚C - 3 min; 35 cycles of 98˚C - 10 sec, 60˚C - 10 sec, 72˚C - 30 sec; 72˚C - 5 min; 4˚C – forever.

3 µl of each PCR are run for QC on 1% agarose gel. PCR products are pooled and purified using the QIAquick PCR Purification Kit (Qiagen) and eluted in 40 µl (2x 20 µl) preheated ddH_2O (to 60˚C) to maximise yield.

DNA amount can be measured here and should range between 15-20µg in total.

Digest is optimised for complete digest of 4mers. 40 units of BciVi will accomplish this in most cases and is still cost-effective. CutSmart buffer and 4 µl BciVi (= 40 units) per reaction are added to a total volume of 50 µl and fragments are digested for 12 hours at 37˚C. Heat inactivation of the enzyme is performed for 20min at 80˚C. Samples are run on 2.5-3% agarose gels using SybrSafe.

Gels have to be as thin as possible to visualise bands of high DNA content and to minimize the amount of agarose in gel purification.

<u>Expected Band sizes:</u>
1 (Longest): 444bp +~80bp
4, 5, 8, 9, 12, 13, 16 (Long): 393bp +~80bp
2, 3, 6, 7, 10, 11, 14, 15 (Short): 145bp+~75-80bp
gel purify with Qiagen kit

DNA is eluted in 30µl (20+10µl) pre-heated ddH_2O. For 4mer assembly, 90 ng of the large fragments (1, 4, 5, 8, 9, 12, 13, 16) and 30 ng of the small fragments (2, 3, 6, 7, 10, 11, 14, 15) are needed, with 1.25 µl of each fragment, therefore it is convenient to adjust the 1mers to the according concentrations (72 ng/µl for big fragments, 24 ng/µl for small fragments).

4mer assembly

A total volume of 1mers of 5µl is mixed with 15 µl Gibson mix and incubated at 50˚C for 30 minutes. In the meanwhile, PlasmidSafe (Epicentre Plasmid-safe Cat No E3105K) Master Mix is prepared as follows:

	Per reaction [µl]
H_2O	13.9
buffer	4
ATP	1.6
enzyme	0.5
Total	*20*

PlasmidSafe is added and incubated for 15 minutes at 37˚C before heat-inactivating immediately at 72˚C for another 15 minutes.

Amplification and digest of 4mers

Use of 0.5 µl 4mer as PCR input with HerculaseII (Agilent Technologies).

Primers used: FAP1: CAAAGTTGCAGCCGAATACA; FAP2: GGCACCGAAGTGCTATGATT.

MasterMix:

	Per reaction [µl]
Buffer	10
dNTPs (10mM)	5
PrimerMix (10µM each)	2.5
Herculase II	0.5
H_2O	31.5
Total	*49.5*

Cycling parameters: 98°C for 2 minutes, 30 cycles of 98°C for 20 seconds, 60°C for 20 seconds and 72°C for 30 seconds, 72°C for 3 minutes.

For blunt end generation, restriction digest using FastDigest SchI (Life Technologies) is performed directly in PCR buffer.

	Per reaction [µl]
DNA	50
Buffer (10x)	10
SchI	1.5
H_2O	88.5
Total	*150*

Incubation for 1 h at 37°C with an additional 5 minutes at 80 °C for heat inactivation of the enzyme.

4mer purification using Agencourt AMPure XP (Beckman-Coulter)

Samples are mixed 1:1 with 15% PEG-8000 (in 1.8 M NaCl; final PEGconc. of 7.5%). It is important that samples are mixed exactly with a 1:1 ratio. Beads are warmed up to RT for 30 min. 20 µl of beads/sample are added and mixed. Incubation for 20 min at RT and before leaving on magnet for 5 min and discarding supernatant. Samples are washed twice with 50 µl of 70% EtOH (left on magnet for washes): incubate for 1 min, take off EtOH and repeat washing step. After wash steps, beads are dried for 2 min on magnet. It is important that there is no remaining alcohol, but beads should not 'overdry', either. DNA is eluted in 40ul H_2O / sample. For this, samples are taken off the magnet, incubated with water for 2 min, returned to magnet for 5 min and samples are collected.

Concentration measurement of clean 4mers is performed on Agilent TapeStation or Qubit Fluorometric Quantitation (Thermo Fisher Scientific). Qubit can overestimate concentration by 3-fold, therefore all 4mers should be measured with the same device to guarantee equimolar ratios in 16mer assembly.

TALE assembly

Prior to the assembly, 5 µg of the respective destination vector are digested with 20 units of BciVI for 12 hours and gel purified. Open backbone needs to be aliquoted to prevent numerous freeze and thaw cycles.

In case of BciVI sites present in the functional domain of choice (e.g. KRAB or p300core), an alternative strategy can be employed to insert DBDs into TALE destination backbones with the STAR method. Instead of linearising the backbone with BciVI, 2µg of the backbone are digested with 10U SbfI-HF (NEB) for 1h at 37˚C with

subsequent heat-inactivation at 80˚C for 20 min. For degradation of 3' overhangs, the open vector is incubated with 2U Mung Bean Endonuclease (NEB) directly in CutSmart buffer for 30 min at 30˚C. The reaction is directly loaded onto an agarose gel and gel extraction of the open backbone is performed.
A total of 51.5 ng of 4mers is assembled with an equimolar ratio, i.e. 14 ng of first 4mer and 12.5 ng each of the three other 4mers with 50 ng backbone of choice. If 4mers are too diluted for a final reaction volume of 5 µl, i.e. in more than 4µl with the backbone at 50ng/µl, they need to be vacuum-concentrated with caution not to overdry them. The backbone is added to the concentrated samples, before addition of 15 µl Gibson mix. Mixture is pipetted up and down several times to mix and wash tube and transfered back to a PCR tube for Gibson assembly of 1h at 50˚C.
1 µl of the Gibson reaction are directly transformed into competent bacteria and grow on Amp resistance plates ON at 37˚C.

Colony PCR is performed with DreamTaq:

	Per reaction [µl]	50x
buffer	2	100
dNTPs	0.4	20
primer mix (STAR2 seq)	2	100
DreamTaq	0.25	12.5
H_2O	14.35	717.5
Total	*19 µl*	*950*

For colony PCR, master mix is prepared in 96 well plate together with a second 96 well plate with 50 µl ddH_2O / well. Bacterial colonies are directly picked with pipette set to 1µl. Tip is first washed in H_2O plate and subsequently 1 µl of H_2O is transferred to the PCR mix plate with the same tip. Products are run on 1% agarose gels and analysed for ~1.77 kb product.
We have chosen DreamTaq polymerase for its cost-effective performance. However, the polymerase can be changed according to user preferences.

Supplementary Table S1. List of primers used in STAR method.

Name	Sequence
684_seq01	CCCAGAAGGTACCCCATTGT
684_seq02	CCACACCTTGCCGATGTC
684_seq03	GGATCTGATCTGGGGCCTC
684_seq04	GCGCGTGAGGAAGAGTTCT
684MOD F	CTGCCGCTTACCGCATAC
684MOD R	ATCTACACGACGGGGAGTCA
dCas9 ampli F	AATTCCTCGAGGACAAGAAGTACAGCATCGGC
dCas9 ampli R	GGGGCTAGCGCTGTCGCCTCCCAG
dCas9 Gibson F	TGAAGCGGCGGAGATACA
dCas9 Gibson R	CGGCTCAGCCTGCC
FAP1	CAAAGTTGCAGCCGAATACA
FAP2	GGCACCGAAGTGCTATGATT
FAP3a	AGTTGCAGCCGAATACAGTG
FAP3b	GCCCTAGACCTGTTGAACGA
FAP4	CCGAAGTGCTATGATTCTCGAC
FAP5	TGACAAAGTTGCAGCCGAAT
FAP6	CCTGCACCTGAGGAGTGAAT
FAP7	AGTTGCAGCCGAATACAGTG
FAP8	GAAGTGCTATGATTCTCGACTCC
FAP9	TGACAAAGTTGCAGCCGAAT
FAP10	CCTGCACCTGAGGAGTGAAT
GG VP160 F1	CGTCTCGCAGTCTCGCGCCGACGC
GG VP160 F2	CGTCTCGCAGTCTCGCGCCGACGCGCTGGA
GG VP160 R	CGTCTCGAGCTCTAGATCATCGATGCA
HA-NLS-dCas9 F	AATTCCTCGAGGCCACCATGTACCCATAC
KRAB GG F	TACGGATCGTCTCGCAGTGGATGGACGCGAAATCA
KRAB GG R	CCGTACGTCTCGAGCTTTATACCAGCCAAGGTTCT
KRAB MISO F 01	GAGGAGTGGAAATTGCTGGACACTGC
KRAB MISO F 02	CTCGTCAGTCTCGGATATCAGTTGAC
KRAB MISO R 01	TTTGTTGCGCAGTGTCCAGCAA
KRAB MISO R 02	CATCCGGTTTCGTCAACTGATATCCGA
p300 GG F	GCAGTGCGTCTCGCAGTC
p300 GG R	GAGCTTCGTCTCGAGCTCTTCAA
p300 MISO F	GTGATGCAAAGCCTTGGGTACTGTT
p300 MISO R	AACTTTCTGCCACAACAGTACCCAAGG
pTAL3	CGCGACGTAATACGACTCAC
pTAL4	CACGACAGGTTTCCCGACT
Seq dCas9 Cterm	CCGGAAGAGGTACACCAGCAC
STAR2 ALL01 F	AGGACACTCGGTTATTCGCA
STAR2 ALL01 R	CCTGGACAATTGGGCTACGA
STAR2 ALL02 F	CCAAGATATGATTGCGGCCC
STAR2 ALL02 R	GCGACTCGATGGGAAGTTCT
STAR2 SEQ F	AGTAACAGCGGTAGAGGCAG
STAR2 SEQ R	ATTGGGCTACGATGGACTCC
STAR2 VP160 F	GGAGTCCATCGTAGCCCAAT
STAR2 VP160 R	TTGTCTTCCCAATCCTCCCC
VP64-Puro GG F	TACGGATCGTCTCGCAGTGGGACGCATTGGACGATTTT
VP64-Puro GG R	CCGTACGTCTCGAGCTTCAGGCACCGGGCTTGCG
VP64-Puro MISO F 01	CACCCGCGACGACGTCCCCAGGGCCGTA
VP64-Puro MISO F 02	GCCACCGTCGGAGTCTCGCCCGACCACCAGGG
VP64-Puro MISO R 01	CCTGGGGACGTCGTCGCGGGTGGCGAGG
VP64-Puro MISO R 02	GGGCGAGACTCCGACGGTGGCCAGGAACCA
VpSeq R	AAGAGTTCTTGCAGCTCGGT
VpSeqF	AGAACTTCCCATCGAGTCGC

TaqMan assays

Gene	Assay	Order no
GAPDH	FAM-MGB	Hs02758991_g1
SOX2	FAM-MGB	Hs01053049_s1

Supplementary Table S2. 1mer library before BciVI digest (All sequences 5' to 3')

Fragment	Length	Sequence - nucleotide sequence and then encoded amino acid sequence
1-HD	588 bp	CGCGACGTAATAC**GACTC**ACTATAGGGCGAATTGGCGGAAGGCCGTCAAGGCCTAGGCGCGCCATGAGCTC*GTATCC*TGCAGCCATTGTGCTGGCGCGGATTCTTTATCACTGATAAGTTGGTGGACATATTAT**GAGTC**GTTTATCAGTGATAAAGTGTCAAGCATGACAAAGTTGCAGCCGAATACAGTG**GAGTC**ATCCGTGCCGCCCTAGACCTGTTGAACGAGGTCGGCGTAGACGGTCTGACGA**GAGTC**CACGCAAACTGGCGGAACGGTTGGGGGTTCAGCAGCCGGCGCTTTA**GAGTC**CTGGCGAAGAGAGGGGGAGTAACAGCGGTAGAGGCAGTGCACGCCTGGCGCAATGCGCTCACCGGGGCCCCCTTGAACCTGACACCTGACCAAGTCGTGGCAATCGCGTCG**CACGAC**GGGGGAAAGCAAGCCCTGGAAACCGTGCAAAGGTTGTTGCCGGTGCTGTGCCAAGACCACGGCTTAACACCAGAACAGGTCGTTCTGCA*GGATAC*GGTACCTCTTAATTAACTGGCCTCATGGGCCTTCCGCTCACTGCCCGCTTTCCAGTCGGGAAACCTGTCGTG
2-HD	292 bp	CGCGACGTAATAC**GACTC**ACTATAGGGCGAATTGGCGGAAGGCCGTCAAGGCCACGTGTCTTGTCCAGAGCTC*GTATCC*TGCAGTTAACACCAGAACAGGTCGTTGCAATCGCGTCG**CACGAC**GGGGGAAAGCAAGCCCTGGAAACCGTGCAAAGGTTGTTGCCGGTTCTATGTCAGGCGCACGGCTTAACTCCAGACCAGGTGGTACTGCA*GGATAC*GGTACCTGGAGCACAAGACTGGCCTCATGGGCCTTCCGCTCACTGCCCGCTTTCCAGTCGGGAAACCTGTCGTG
3-HD	292 bp	CGCGACGTAATAC**GACTC**ACTATAGGGCGAATTGGCGGAAGGCCGTCAAGGCCACGTGTCTTGTCCAGAGCTC*GTATCC*TGCAGTTAACTCCAGACCAGGTGGTAGCAATCGCGTCG**CACGAC**GGGGGAAAGCAAGCCCTGGAAACCGTGCAAAGGTTGTTGCCGGTACTATGCCAGGCACATGGATTAACCCCAGCTCAAGTGGTACTGCA*GGATAC*GGTACCTGGAGCACAAGACTGGCCTCATGGGCCTTCCGCTCACTGCCCGCTTTCCAGTCGGGAAACCTGTCGTG
4-HD	510 bp	CGCGACGTAATAC**GACTC**ACTATAGGGCGAATTGGCGGAAGGCCGTCAAGGCCTAGGCGCGCCATGAGCTC*GTATCC*TGCAGTTAACCCCAGCTCAAGTGGTAGCAATCGCGTCG**CACGAC**GGGGGAAAGCAAGCCCTGGAAACCGTGCAAAGGTTGTTGCCGGTCTTGTGCCAAGATCATGGGCTTCA**GACTC**GAACAAGCGGGCGCTGCTCGACGCACTGGCCGAAGCCATGCTGGCG**GAGTC**GAGAATCATAGCACTTCGGTGCCGAGAGCCGACGACGACTGGCGCTCATTTC**GAGTC**TGACTGGGAATGCCCGCAGCTTCAGGCAGGCGCTGCTCGCCTACCGCCAGCA**GAGTC**CAATGGATCTCGAGGAATTCACTCCTCAGGTGCAGGCTGCCTATCAG**GAGTC**CCATTGTGCTGGCGCGCTGCA*GGATAC*GGTACCTCTTAATTAACTGGCCTCATGGGCCTTCCGCTCACTGCCCGCTTTCCAGTCGGGAAACCTGTCGTG

5-HD	540 bp	CGCGACGTAATAC**GACTC**ACTATAGGGCGAATTGGCGGAAGGCCGTCAAGGCCACGTGTCTTGTCCAGAGCTC*GTATCC*TGCAGCCATTGTGCTGGCGCGGATTCTTTATCACTGATAAGTTGGTGGACATATTAT**GAGTC**GTTTATCAGTGATAAAGTGTCAAGCATGACAAAGTTGCAGCCGAATACAGTG**GAGTC**ATCCGTGCCGCCCTAGACCTGTTGAACGAGGTCGGCGTAGACGGTCTGACGA**GAGTC**CACGCAAACTGGCGGAACGGTTGGGGGTTCAGCAGCCGGCGCTTTA**GAGTC**CTGGCGTCTTGTGCCAAGATCATGGCTTACTCCCGATCAGGTGGTCGCAATCGCGTCG**CACGAC**GGGGGAAAGCAAGCCCTGGAAACCGTGCAAAGGTTGTTGCCGGTACTTTGCCAGGACCATGGATTAACTCCTGAGCAAGTGGTCCTGCA*GGATAC*GGTACCTGGAGCACAAGACTGGCCTCATGGCCTTCCGCTCACTGCCCGCTTTCCAGTCGGGAAACCTGTCGTG
6-HD	292 bp	CGCGACGTAATAC**GACTC**ACTATAGGGCGAATTGGCGGAAGGCCGTCAAGGCCACGTGTCTTGTCCAGAGCTC*GTATCC*TGCAGTTAACTCCTGAGCAAGTGGTCGCAATCGCGTCG**CACGAC**GGGGGAAAGCAAGCCCTGGAAACCGTGCAAAGGTTGTTGCCGGTGTTGTGCCAAGCACATGGCTTAACACCGGATCAAGTCGTGCTGCA*GGATAC*GGTACCTGGAGCACAAGACTGGCCTCATGGCCTTCCGCTCACTGCCCGCTTTCCAGTCGGGAAACCTGTCGTG
7-HD	292 bp	CGCGACGTAATAC**GACTC**ACTATAGGGCGAATTGGCGGAAGGCCGTCAAGGCCACGTGTCTTGTCCAGAGCTC*GTATCC*TGCAGTTAACACCGGATCAAGTCGTGGCAATCGCGTCG**CACGAC**GGGGGAAAGCAAGCCCTGGAAACCGTGCAAAGGTTGTTGCCGGTCCTGTGTCAGGCGCATGGATTAACGCCCGCACAGGTTGTTCTGCA*GGATAC*GGTACCTGGAGCACAAGACTGGCCTCATGGCCTTCCGCTCACTGCCCGCTTTCCAGTCGGGAAACCTGTCGTG
8-HD	514 bp	CGCGACGTAATAC**GACTC**ACTATAGGGCGAATTGGCGGAAGGCCGTCAAGGCCACGTGTCTTGTCCAGAGCTC*GTATCC*TGCAGTTAACGCCCGCACAGGTTGTTGCAATCGCGTCG**CACGAC**GGGGGAAAGCAAGCCCTGGAAACCGTGCAAAGGTTGTTGCCGGTTCTATGCCAGGATCACGGGCTTCA**GACTC**GAACAAGCGGGCGCTGCTCGACGCACTGGCCGAAGCCATGCTGGCG**GAGTC**GAGAATCATAGCACTTCGGTGCCGAGAGCCGACGACGACTGGCGCTCATTTC**GAGTC**TGACTGGGAATGCCCGCAGCTTCAGGCAGGCGCTGCTCGCCTACCGCCAGCA**GAGTC**CAATGGATCTCGAGGAATTCACTCCTCAGGTGCAGGCTGCCTATCAG**GAGTC**CCATTGTGCTGGCGCGCTGCA*GGATAC*GGTACCTGGAGCACAAGACTGGCCTCATGGCCTTCCGCTCACTGCCCGCTTTCCAGTCGGGAAACCTGTCGTG
9-HD	540 bp	CGCGACGTAATAC**GACTC**ACTATAGGGCGAATTGGCGGAAGGCCGTCAAGGCCACGTGTCTTGTCCAGAGCTC*GTATCC*TGCAGCCATTGTGCTGGCGCGGATTCTTTATCACTGATAAGTTGGTGGACATATTAT**GAGTC**GTTTATCAGTGATAAAGTGTCAAGCATGACAAAGTTGCAGCCGAATACAGTG**GAGTC**ATCCGTGCCGCCCTAGACCTGTTGAACGAGGTCGGCGTAGACGGTCTGACGA**GAGTC**CACGCAAACTGGCGGAACGGTTGGGGGTTCAGCAGCCGGCGCTTTA**GAGTC**CTGGCGTTCTATGCCAGGATCACGGGCTTA

		CCCCGGACCAAGTAGTAGCAATCGCGTCG**CACGAC**GGGGGAAAGCAAGCCCTGGAAACCGTGCAAAGGTTGTTGCCGGTTTTAT<br>GTCAAGACCACGGTTTAACCCCCGAACAGGTAGTGCTGCA*GGATAC*GGTACCTGGAGCACAAGACTGGCCTCATGGGCCTTCCG<br>CTCACTGCCCGCTTTCCAGTCGGGAAACCTGTCGTG
10-HD	292 bp	CGCGACGTAATAC**GACTC**ACTATAGGGCGAATTGGCGGAAGGCCGTCAAGGCCACGTGTCTTGTCCAGAGCTC*GTATCC*TGCAG<br>TTAACCCCCGAACAGGTAGTGGCAATCGCGTCG**CACGAC**GGGGGAAAGCAAGCCCTGGAAACCGTGCAAAGGTTGTTGCCGGTC<br>CTCTGTCAGGCTCACGGATTAACGCCCGACCAGGTTGTTCTGCA*GGATAC*GGTACCTGGAGCACAAGACTGGCCTCATGGGCCT<br>TCCGCTCACTGCCCGCTTTCCAGTCGGGAAACCTGTCGTG
11-HD	292 bp	CGCGACGTAATAC**GACTC**ACTATAGGGCGAATTGGCGGAAGGCCGTCAAGGCCTAGGCGCGCCATGAGCTC*GTATCC*TGCAGTT<br>AACGCCCGACCAGGTTGTTGCAATCGCGTCG**CACGAC**GGGGGAAAGCAAGCCCTGGAAACCGTGCAAAGGTTGTTGCCGGTGTT<br>GTGCCAAGCGCACGGCTTAACGCCGGCCCAAGTAGTCCTGCA*GGATAC*GGTACCTCTTAATTAACTGGCCTCATGGGCCTTCCG<br>CTCACTGCCCGCTTTCCAGTCGGGAAACCTGTCGTG
12-HD	514 bp	CGCGACGTAATAC**GACTC**ACTATAGGGCGAATTGGCGGAAGGCCGTCAAGGCCACGTGTCTTGTCCAGAGCTC*GTATCCT*GCAG<br>TTAACGCCGGCCCAAGTAGTCGCAATCGCGTCG**CACGAC**GGGGGAAAGCAAGCCCTGGAAACCGTGCAAAGGTTGTTGCCGGTA<br>TTATGTCAAGATCATGGGCTTCA**GACTC**GAACAAGCGGGCGCTGCTCGACGCACTGGCCGAAGCCATGCTGGCG**GAGTC**GAGAA<br>TCATAGCACTTCGGTGCCGAGAGCCGACGACGACTGGCGCTCATTTC**GAGTC**TGACTGGGAATGCCCGCAGCTTCAGGCAGGCG<br>CTGCTCGCCTACCGCCAGCA**GAGTC**CAATGGATCTCGAGGAATTCACTCCTCAGGTGCAGGCTGCCTATCAG**GAGTC**CCATTGT<br>GCTGGCGCGCTGCA*GGATAC*GGTACCTGGAGCACAAGACTGGCCTCATGGGCCTTCCGCTCACTGCCCGCTTTCCAGTCGGGAA<br>ACCTGTCGTG
13-HD	540 bp	CGCGACGTAATAC**GACTC**ACTATAGGGCGAATTGGCGGAAGGCCGTCAAGGCCACGTGTCTTGTCCAGAGCTC*GTATCC*TGCAG<br>CCATTGTGCTGGCGCGGATTCTTTATCACTGATAAGTTGGTGGACATATTAT**GAGTC**GTTTATCAGTGATAAAGTGTCAAGCAT<br>GACAAAGTTGCAGCCGAATACAGTG**GAGTC**ATCCGTGCCGCCCTAGACCTGTTGAACGAGGTCGGCGTAGACGGTCTGACGA**GA**<br>**GTC**CACGCAAACTGGCGGAACGGTTGGGGGTTCAGCAGCCGGCGCTTTA**GAGTC**CTGGCGTATTATGTCAAGATCATGGGCTCA<br>CGCCAGATCAGGTTGTTGCAATCGCGTCG**CACGAC**GGGGGAAAGCAAGCCCTGGAAACCGTGCAAAGGTTGTTGCCGGTCTTGT<br>GTCAGGACCATGGCTTAACGCCGGAGCAAGTTGTACTGCA*GGATAC*GGTACCTGGAGCACAAGACTGGCCTCATGGGCCTTCCG<br>CTCACTGCCCGCTTTCCAGTCGGGAAACCTGTCGTG

14-HD	292 bp	CGCGACGTAATAC**GACTC**ACTATAGGGCGAATTGGCGGAAGGCCGTCAAGGCCACGTGTCTTGTCCAGAGCTC*GTATCC*TGCAGTTAACGCCGGAGCAAGTTGTAGCAATCGCGTCG**CACGAC**GGGGGAAAGCAAGCCCTGGAAACCGTGCAAAGGTTGTTGCCGGTATTATGCCAAGCCCATGGTTTAACCCCTGATCAAGTAGTCCTGCA*GGATAC*GGTACCTGGAGCACAAGACTGGCCTCATGGGCCTTCCGCTCACTGCCCGCTTTCCAGTCGGGAAACCTGTCGTG
15-HD	292 bp	CGCGACGTAATAC**GACTC**ACTATAGGGCGAATTGGCGGAAGGCCGTCAAGGCCACGTGTCTTGTCCAGAGCTC*GTATCC*TGCAGTTAACCCCTGATCAAGTAGTCGCAATCGCGTCG**CACGAC**GGGGGAAAGCAAGCCCTGGAAACCGTGCAAAGGTTGTTGCCGGTTCTCTGTCAAGCCCACGGTTTAACTCCGGCGCAGGTCGTGCTGCA*GGATAC*GGTACCTGGAGCACAAGACTGGCCTCATGGGCCTTCCGCTCACTGCCCGCTTTCCAGTCGGGAAACCTGTCGTG
16-HD	514 bp	CGCGACGTAATAC**GACTC**ACTATAGGGCGAATTGGCGGAAGGCCGTCAAGGCCACGTGTCTTGTCCAGAGCTC*GTATCC*TGCAGTTAACTCCGGCGCAGGTCGTGGCAATCGCGTCG**CACGAC**GGGGGAAAGCAAGCCCTGGAAACCGTGCAAAGGTTGTTGCCGGTGCTCTGTCAGGACCACGGGCTTCA**GACTC**GAACAAGCGGGCGCTGCTCGACGCACTGGCCGAAGCCATGCTGGCG**GAGTC**GAGAATCATAGCACTTCGGTGCCGAGAGCCGACGACGACTGGCGCTCATTTC**GAGTC**TGACTGGGAATGCCCGCAGCTTCAGGCAGGCGCTGCTCGCCTACCGCCAGCA**GAGTC**CAATGGATCTCGAGGAATTCACTCCTCAGGTGCAGGCTGCCTATCAG**GAGTC**CCATTGTGCTGGCGCGCTGCA*GGATAC*GGTACCTGGAGCACAAGACTGGCCTCATGGGCCTTCCGCTCACTGCCCGCTTTCCAGTCGGGAAACCTGTCGTG

pTAL3 and pTAL4 primer sequences for library amplification are underlined.

FAP1 and FAP2 primers for 4mer amplification are double underlined.

BciVI recognition sites (GTATCC and GGATAC) are shown in *italics*. Sequences removed after BciVI digest are highlighted in yellow.

HD recognition site is shown in **bold**, and the alternative RVD sequences are:

- NH: AAT CAC
- NI: AAC ATT
- NG: AAT GGA

MlyI / SchI recognition sites (5’ GAGTC 3’ and 5’ GACTC 3’) are shown in **bold and underlined**. Sequences removed after MlyI digest are printed in orange.

EPC Sequences:

5’ EPC

CCATTGTGCTGGCGCGGATTCTTTATCACTGATAAGTTGGTGGACATATTATGAGTCGTTTATCAGTGATAAAGTGTCAAGCATGACAAAGT
TGCAGCCGAATACAGTGGAGTCATCCGTGCCGCCCTAGACCTGTTGAACGAGGTCGGCGTAGACGGTCTGACGAGAGTCCACGCAAACT
GGCGGAACGGTTGGGGGTTCAGCAGCCGGCGCTTTAGAGTCCTGGC

3’ EPC

CTTCAGACTCGAACAAGCGGGCGCTGCTCGACGCACTGGCCGAAGCCATGCTGGCGGAGTCGAGAATCATAGCACTTCGGTGCCGAGAG
CCGACGACGACTGGCGCTCATTTCGAGTCTGACTGGGAATGCCCGCAGCTTCAGGCAGGCGCTGCTCGCCTACCGCCAGCAGAGTCCAA
TGGATCTCGAGGAATTCACTCCTCAGGTGCAGGCTGCCTATCAGGAGTCCCATTGTGCTGGCGCG

Supplementary Table S3. Sequences of 1mer library ordered as custom genes (All sequences 5' to 3')

1mer library was ordered as custom genes from Life Technologies. Sequences given in the following table were provided to Life Technologies for synthesis and subsequent cloning into vector backbones pMA and pMA-T, respectively.

Fragment	Length	Sequence
HD-1	445 bp	GTATCCTGCAGCCATTGTGCTGGCGCGGATTCTTTATCACTGATAAGTTGGTGGACATATTATGAGTCGTTTATCAGTG<br>ATAAAGTGTCAAGCATGACAAAGTTGCAGCCGAATACAGTGGAGTCATCCGTGCCGCCCTAGACCTGTTGAACGAGGT<br>CGGCGTAGACGGTCTGACGAGAGTCCACGCAAACTGGCGGAACGGTTGGGGGTTCAGCAGCCGGCGCTTTAGAGTC<br>CTGGCGAAGAGAGGGGGAGTAACAGCGGTAGAGGCAGTGCACGCCTGGCGCAATGCGCTCACCGGGGCCCCCTTGA<br>ACCTGACACCTGACCAAGTCGTGGCAATCGCGTCGCACGACGGGGGAAAGCAAGCCCTGGAAACCGTGCAAAGGTTG<br>TTGCCGGTGCTGTGCCAAGACCACGGCTTAACACCAGAACAGGTCGTTCTGCAGGATAC
HD-2	145 bp	GTATCCTGCAGTTAACACCAGAACAGGTCGTTGCAATCGCGTCGCACGACGGGGGAAAGCAAGCCCTGGAAACCGTG<br>CAAAGGTTGTTGCCGGTTCTATGTCAGGCGCACGGCTTAACTCCAGACCAGGTGGTACTGCAGGATAC
HD-3	145 bp	GTATCCTGCAGTTAACTCCAGACCAGGTGGTAGCAATCGCGTCGCACGACGGGGGAAAGCAAGCCCTGGAAACCGTG<br>CAAAGGTTGTTGCCGGTACTATGCCAGGCACATGGATTAACCCCAGCTCAAGTGGTACTGCAGGATAC
HD-4	367 bp	GTATCCTGCAGTTAACCCCAGCTCAAGTGGTAGCAATCGCGTCGCACGACGGGGGAAAGCAAGCCCTGGAAACCGTG<br>CAAAGGTTGTTGCCGGTCTTGTGCCAAGATCATGGGCTTCAGACTCGAACAAGCGGGCGCTGCTCGACGCACTGGCC<br>GAAGCCATGCTGGCGGAGTCGAGAATCATAGCACTTCGGTGCCGAGAGCCGACGACGACTGGCGCTCATTTCGAGTC<br>TGACTGGGAATGCCCGCAGCTTCAGGCAGGCGCTGCTCGCCTACCGCCAGCAGAGTCCAATGGATCTCGAGGAATTC<br>ACTCCTCAGGTGCAGGCTGCCTATCAGGAGTCCCATTGTGCTGGCGCGCTGCAGGATAC
HD-5	393 bp	GTATCCTGCAGCCATTGTGCTGGCGCGGATTCTTTATCACTGATAAGTTGGTGGACATATTATGAGTCGTTTATCAGTG<br>ATAAAGTGTCAAGCATGACAAAGTTGCAGCCGAATACAGTGGAGTCATCCGTGCCGCCCTAGACCTGTTGAACGAGGT<br>CGGCGTAGACGGTCTGACGAGAGTCCACGCAAACTGGCGGAACGGTTGGGGGTTCAGCAGCCGGCGCTTTAGAGTC<br>CTGGCGTCTTGTGCCAAGATCATGGGCTTACTCCCGATCAGGTGGTCGCAATCGCGTCGCACGACGGGGGAAAGCAA<br>GCCCTGGAAACCGTGCAAAGGTTGTTGCCGGTACTTTGCCAGGACCATGGATTAACTCCTGAGCAAGTGGTCCTGCAG<br>GATAC
HD-6	145 bp	GTATCCTGCAGTTAACTCCTGAGCAAGTGGTCGCAATCGCGTCGCACGACGGGGGAAAGCAAGCCCTGGAAACCGTG<br>CAAAGGTTGTTGCCGGTGTTGTGCCAAGCACATGGCTTAACACCGGATCAAGTCGTGCTGCAGGATAC
HD-7	145 bp	GTATCCTGCAGTTAACACCGGATCAAGTCGTGGCAATCGCGTCGCACGACGGGGGAAAGCAAGCCCTGGAAACCGTG<br>CAAAGGTTGTTGCCGGTCCTGTGTCAGGCGCATGGATTAACGCCCGCACAGGTTGTTCTGCAGGATAC
HD-8	367 bp	GTATCCTGCAGTTAACGCCCGCACAGGTTGTTGCAATCGCGTCGCACGACGGGGGAAAGCAAGCCCTGGAAACCGTG<br>CAAAGGTTGTTGCCGGTTCTATGCCAGGATCACGGGCTTCAGACTCGAACAAGCGGGCGCTGCTCGACGCACTGGCC

		GAAGCCATGCTGGCGGAGTCGAGAATCATAGCACTTCGGTGCCGAGAGCCGACGACGACTGGCGCTCATTTCGAGTC<br>TGACTGGGAATGCCCGCAGCTTCAGGCAGGCGCTGCTCGCCTACCGCCAGCAGAGTCCAATGGATCTCGAGGAATTC<br>ACTCCTCAGGTGCAGGCTGCCTATCAGGAGTCCCATTGTGCTGGCGCGCTGCAGGATAC
HD-9	393 bp	GTATCCTGCAGCCATTGTGCTGGCGCGGATTCTTTATCACTGATAAGTTGGTGGACATATTATGAGTCGTTTATCAGTG<br>ATAAAGTGTCAAGCATGACAAAGTTGCAGCCGAATACAGTGGAGTCATCCGTGCCGCCCTAGACCTGTTGAACGAGGT<br>CGGCGTAGACGGTCTGACGAGAGTCCACGCAAACTGGCGGAACGGTTGGGGGTTCAGCAGCCGGCGCTTTAGAGTC<br>CTGGCGTTCTATGCCAGGATCACGGGCTTACCCCGGACCAAGTAGTAGCAATCGCGTCGCACGACGGGGGAAAGCAA<br>GCCCTGGAAACCGTGCAAAGGTTGTTGCCGGTTTTATGTCAAGACCACGGTTTAACCCCCGAACAGGTAGTGCTGCAG<br>GATAC
HD-10	145 bp	GTATCCTGCAGTTAACCCCCGAACAGGTAGTGGCAATCGCGTCGCACGACGGGGGAAAGCAAGCCCTGGAAACCGTG<br>CAAAGGTTGTTGCCGGTCCTCTGTCAGGCTCACGGATTAACGCCCGACCAGGTTGTTCTGCAGGATAC
HD-11	145 bp	GTATCCTGCAGTTAACGCCCGACCAGGTTGTTGCAATCGCGTCGCACGACGGGGGAAAGCAAGCCCTGGAAACCGTG<br>CAAAGGTTGTTGCCGGTGTTGTGCCAAGCGCACGGCTTAACGCCGGCCCAAGTAGTCCTGCAGGATAC
HD-12	367 bp	GTATCCTGCAGTTAACGCCGGCCCAAGTAGTCGCAATCGCGTCGCACGACGGGGGAAAGCAAGCCCTGGAAACCGTG<br>CAAAGGTTGTTGCCGGTATTATGTCAAGATCATGGGCTTCAGACTCGAACAAGCGGGCGCTGCTCGACGCACTGGCC<br>GAAGCCATGCTGGCGGAGTCGAGAATCATAGCACTTCGGTGCCGAGAGCCGACGACGACTGGCGCTCATTTCGAGTC<br>TGACTGGGAATGCCCGCAGCTTCAGGCAGGCGCTGCTCGCCTACCGCCAGCAGAGTCCAATGGATCTCGAGGAATTC<br>ACTCCTCAGGTGCAGGCTGCCTATCAGGAGTCCCATTGTGCTGGCGCGCTGCAGGATAC
HD-13	393 bp	GTATCCTGCAGCCATTGTGCTGGCGCGGATTCTTTATCACTGATAAGTTGGTGGACATATTATGAGTCGTTTATCAGTG<br>ATAAAGTGTCAAGCATGACAAAGTTGCAGCCGAATACAGTGGAGTCATCCGTGCCGCCCTAGACCTGTTGAACGAGGT<br>CGGCGTAGACGGTCTGACGAGAGTCCACGCAAACTGGCGGAACGGTTGGGGGTTCAGCAGCCGGCGCTTTAGAGTC<br>CTGGCGTATTATGTCAAGATCATGGGCTCACGCCAGATCAGGTTGTTGCAATCGCGTCGCACGACGGGGGAAAGCAA<br>GCCCTGGAAACCGTGCAAAGGTTGTTGCCGGTCTTGTGTCAGGACCATGGCTTAACGCCGGAGCAAGTTGTACTGCA<br>GGATAC
HD-14	145 bp	GTATCCTGCAGTTAACGCCGGAGCAAGTTGTAGCAATCGCGTCGCACGACGGGGGAAAGCAAGCCCTGGAAACCGTG<br>CAAAGGTTGTTGCCGGTATTATGCCAAGCCCATGGTTTAACCCCTGATCAAGTAGTCCTGCAGGATAC
HD-15	145 bp	GTATCCTGCAGTTAACCCCTGATCAAGTAGTCGCAATCGCGTCGCACGACGGGGGAAAGCAAGCCCTGGAAACCGTG<br>CAAAGGTTGTTGCCGGTTCTCTGTCAAGCCCACGGTTTAACTCCGGCGCAGGTCGTGCTGCAGGATAC
HD-16	367 bp	GTATCCTGCAGTTAACTCCGGCGCAGGTCGTGGCAATCGCGTCGCACGACGGGGGAAAGCAAGCCCTGGAAACCGTG<br>CAAAGGTTGTTGCCGGTGCTCTGTCAGGACCACGGGCTTCAGACTCGAACAAGCGGGCGCTGCTCGACGCACTGGCC<br>GAAGCCATGCTGGCGGAGTCGAGAATCATAGCACTTCGGTGCCGAGAGCCGACGACGACTGGCGCTCATTTCGAGTC<br>TGACTGGGAATGCCCGCAGCTTCAGGCAGGCGCTGCTCGCCTACCGCCAGCAGAGTCCAATGGATCTCGAGGAATTC<br>ACTCCTCAGGTGCAGGCTGCCTATCAGGAGTCCCATTGTGCTGGCGCGCTGCAGGATAC

All custom gene sequences shown shown for RVD HD (CAC GAC; highlitghted in green).

Custom genes for other monomers only differ in RVDs:
NI – AAC ATT
NG – AAT GGA
NH – AAT CAC

Overlapping ends of monomer repeats that will be available after MlyI / SchI digest are highlighted in yellow.

General vector backbones used for cloning of custom genes by Life Technologies:

	HD	NI	NG	NH
**1**	pMA	pMA-T	pMA-T	pMA-T
**2**	pMA-T	pMA-T	pMA-T	pMA
**3**	pMA-T	pMA-T	pMA	pMA-T
**4**	pMA	pMA-T	pMA	pMA-T
**5**	pMA-T	pMA-T	pMA	pMA-T
**6**	pMA-T	pMA-T	pMA-T	pMA-T
**7**	pMA-T	pMA-T	pMA-T	pMA-T
**8**	pMA-T	pMA-T	pMA-T	pMA
**9**	pMA-T	pMA-T	pMA-T	pMA-T
**10**	pMA-T	pMA-T	pMA-T	pMA-T
**11**	pMA	pMA	pMA	pMA
**12**	pMA-T	pMA-T	pMA-T	pMA
**13**	pMA-T	pMA-T	pMA-T	pMA-T
**14**	pMA-T	pMA	pMA-T	pMA-T

**15**	pMA-T	pMA-T	pMA-T	pMA-T
**16**	pMA-T	pMA-T	pMA-T	pMA-T

Sequence vector pMA:

ctaaattgtaagcgttaatattttgttaaaattcgcgttaaatttttgttaaatcagctcattttttaaccaataggccgaaatcggcaaaatcccttataaatcaaaagaatagaccgagatagggttgagtggccgcta
cagggcgctcccattcgccattcaggctgcgcaactgttgggaagggcgtttcggtgcgggcctcttcgctattacgccagctggcgaaaggggatgtgctgcaaggcgattaagttgggtaacgccagggtttt
cccagtcacgacgttgtaaaacgacggccagtgagcgcgacgtaatacgactcactatagggcgaattggcggaaggccgtcaaggcctaggcgcgccatgagctctaaagcttcgctcgagctgcggccg
cacggatcctcgaattcccaagcttatctcgagtagcggccgcttggatcccagaattctaggtacctcttaattaactggcctcatgggccttccgctcactgcccgctttccagtcgggaaacctgtcgtgccagct
gcattaacatggtcatagctgtttccttgcgtattgggcgctctccgcttcctcgctcactgactcgctgcgctcggtcgttcgggtaaagcctggggtgcctaatgagcaaaaggccagcaaaaggccaggaacc
gtaaaaaggccgcgttgctggcgtttttccataggctccgcccccctgacgagcatcacaaaaatcgacgctcaagtcagaggtggcgaaacccgacaggactataaagataccaggcgtttccccctggaa
gctccctcgtgcgctctcctgttccgaccctgccgcttaccggatacctgtccgcctttctcccttcgggaagcgtggcgctttctcatagctcacgctgtaggtatctcagttcggtgtaggtcgttcgctccaagctggg
ctgtgtgcacgaaccccccgttcagcccgaccgctgcgccttatccggtaactatcgtcttgagtccaacccggtaagacacgacttatcgccactggcagcagccactggtaacaggattagcagagcgaggt
atgtaggcggtgctacagagttcttgaagtggtggcctaactacggctacactagaagaacagtatttggtatctgcgctctgctgaagccagttaccttcggaaaaagagttggtagctcttgatccggcaaacaa
accaccgctggtagcggtggtttttttgtttgcaagcagcagattacgcgcagaaaaaaaggatctcaagaagatcctttgatcttttctacggggtctgacgctcagtggaacgaaaactcacgttaagggattttg
gtcatgagattatcaaaaaggatcttcacctagatccttttaaattaaaaatgaagttttaaatcaatctaaagtatatatgagtaaacttggtctgacagttaccaatgcttaatcagtgaggcacctatctcagcgatc
tgtctatttcgttcatccatagttgcctgactccccgtcgtgtagataactacgatacgggagggcttaccatctggccccagtgctgcaatgataccgcgagaaccacgctcaccggctccagatttatcagcaata
aaccagccagccggaagggccgagcgcagaagtggtcctgcaactttatccgcctccatccagtctattaattgttgccgggaagctagagtaagtagttcgccagttaatagtttgcgcaacgttgttgccattgc
tacaggcatcgtggtgtcacgctcgtcgtttggtatggcttcattcagctccggttcccaacgatcaaggcgagttacatgatcccccatgttgtgcaaaaaagcggttagctccttcggtcctccgatcgttgtcagaa
gtaagttggccgcagtgttatcactcatggttatggcagcactgcataattctcttactgtcatgccatccgtaagatgcttttctgtgactggtgagtactcaaccaagtcattctgagaatagtgtatgcggcgaccga
gttgctcttgcccggcgtcaatacgggataataccgcgccacatagcagaactttaaaagtgctcatcattggaaaacgttcttcggggcgaaaactctcaaggatcttaccgctgttgagatccagttcgatgtaa
cccactcgtgcacccaactgatcttcagcatcttttactttcaccagcgtttctgggtgagcaaaaacaggaaggcaaaatgccgcaaaaaagggaataagggcgacacggaaatgttgaatactcatactcttc
ctttttcaatattattgaagcatttatcagggttattgtctcatgagcggatacatatttgaatgtatttagaaaaataaacaaataggggttccgcgcacatttccccgaaaagtgccac

Sequence vector pMA-T:

ctaaattgtaagcgttaatattttgttaaaattcgcgttaaatttttgttaaatcagctcattttttaaccaataggccgaaatcggcaaaatcccttataaatcaaaagaatagaccgagatagggttgagtggccgcta
cagggcgctcccattcgccattcaggctgcgcaactgttgggaagggcgtttcggtgcgggcctcttcgctattacgccagctggcgaaaggggatgtgctgcaaggcgattaagttgggtaacgccagggtttt
cccagtcacgacgttgtaaaacgacggccagtgagcgcgacgtaatacgactcactatagggcgaattggcggaaggccgtcaaggccacgtgtcttgtccnnnggagcacaagactggcctcatgggcct
tccgctcactgcccgctttccagtcgggaaacctgtcgtgccagctgcattaacatggtcatagctgtttccttgcgtattgggcgctctccgcttcctcgctcactgactcgctgcgctcggtcgttcgggtaaagcctg
gggtgcctaatgagcaaaaggccagcaaaaggccaggaaccgtaaaaaggccgcgttgctggcgtttttccataggctccgcccccctgacgagcatcacaaaaatcgacgctcaagtcagaggtggcga
aacccgacaggactataaagataccaggcgtttccccctggaagctccctcgtgcgctctcctgttccgaccctgccgcttaccggatacctgtccgcctttctcccttcgggaagcgtggcgctttctcatagctcac
gctgtaggtatctcagttcggtgtaggtcgttcgctccaagctgggctgtgtgcacgaaccccccgttcagcccgaccgctgcgccttatccggtaactatcgtcttgagtccaacccggtaagacacgacttatcgc
cactggcagcagccactggtaacaggattagcagagcgaggtatgtaggcggtgctacagagttcttgaagtggtggcctaactacggctacactagaagaacagtatttggtatctgcgctctgctgaagcca
gttaccttcggaaaaagagttggtagctcttgatccggcaaacaaaccaccgctggtagcggtggtttttttgtttgcaagcagcagattacgcgcagaaaaaaaggatctcaagaagatcctttgatcttttctacg
gggtctgacgctcagtggaacgaaaactcacgttaagggattttggtcatgagattatcaaaaaggatcttcacctagatccttttaaattaaaaatgaagttttaaatcaatctaaagtatatatgagtaaacttggtc
tgacagttaccaatgcttaatcagtgaggcacctatctcagcgatctgtctatttcgttcatccatagttgcctgactccccgtcgtgtagataactacgatacgggagggcttaccatctggccccagtgctgcaatga

taccgcgagaaccacgctcaccggctccagatttatcagcaataaaccagccagccggaagggccgagcgcagaagtggtcctgcaactttatccgcctccatccagtctattaattgttgccgggaagctag
agtaagtagttcgccagttaatagtttgcgcaacgttgttgccattgctacaggcatcgtggtgtcacgctcgtcgtttggtatggcttcattcagctccggttcccaacgatcaaggcgagttacatgatcccccatgtt
gtgcaaaaaagcggttagctccttcggtcctccgatcgttgtcagaagtaagttggccgcagtgttatcactcatggttatggcagcactgcataattctcttactgtcatgccatccgtaagatgcttttctgtgactggt
gagtactcaaccaagtcattctgagaatagtgtatgcggcgaccgagttgctcttgcccggcgtcaatacgggataataccgcgccacatagcagaactttaaaagtgctcatcattggaaaacgttcttcggggc
gaaaactctcaaggatcttaccgctgttgagatccagttcgatgtaacccactcgtgcacccaactgatcttcagcatcttttactttcaccagcgtttctgggtgagcaaaaacaggaaggcaaaatgccgcaaa
aaagggaataagggcgacacggaaatgttgaatactcatactcttcctttttcaatattattgaagcatttatcagggttattgtctcatgagcggatacatatttgaatgtatttagaaaaataaacaaataggggttcc
gcgcacatttccccgaaaagtgccac

Example vector pMA with HD-1 inserted and highlighted in yellow:
CTAAATTGTAAGCGTTAATATTTTGTTAAAATTCGCGTTAAATTTTTGTTAAATCAGCTCATTTTTTAACCAATAGGCCGAAATCGGCAAAATCCC
TTATAAATCAAAAGAATAGACCGAGATAGGGTTGAGTGGCCGCTACAGGGCGCTCCCATTCGCCATTCAGGCTGCGCAACTGTTGGGAAGGG
CGTTTCGGTGCGGGCCTCTTCGCTATTACGCCAGCTGGCGAAAGGGGGATGTGCTGCAAGGCGATTAAGTTGGGTAACGCCAGGGTTTTCCC
AGTCACGACGTTGTAAAACGACGGCCAGTGAGCGCGACGTAATACGACTCACTATAGGGCGAATTGGCGGAAGGCCGTCAAGGCCTAGGCG
CGCCATGAGCTCGTATCCTGCAGCCATTGTGCTGGCGCGGATTCTTTATCACTGATAAGTTGGTGGACATATTATGAGTCGTTTATCAGTGATA
AAGTGTCAAGCATGACAAAGTTGCAGCCGAATACAGTGGAGTCATCCGTGCCGCCCTAGACCTGTTGAACGAGGTCGGCGTAGACGGTCTGA
CGAGAGTCCACGCAAACTGGCGGAACGGTTGGGGGTTCAGCAGCCGGCGCTTTAGAGTCCTGGCGAAGAGAGGGGGAGTAACAGCGGTAG
AGGCAGTGCACGCCTGGCGCAATGCGCTCACCGGGGCCCCCTTGAACCTGACACCTGACCAAGTCGTGGCAATCGCGTCGCACGACGGGG
GAAAGCAAGCCCTGGAAACCGTGCAAAGGTTGTTGCCGGTGCTGTGCCAAGACCACGGCTTAACACCAGAACAGGTCGTTCTGCAGGATACG
GTACCTCTTAATTAACTGGCCTCATGGGCCTTCCGCTCACTGCCCGCTTTCCAGTCGGGAAACCTGTCGTGCCAGCTGCATTAACATGGTCAT
AGCTGTTTCCTTGCGTATTGGGCGCTCTCCGCTTCCTCGCTCACTGACTCGCTGCGCTCGGTCGTTCGGGTAAAGCCTGGGGTGCCTAATGA
GCAAAAGGCCAGCAAAAGGCCAGGAACCGTAAAAAGGCCGCGTTGCTGGCGTTTTTCCATAGGCTCCGCCCCCCTGACGAGCATCACAAAAA
TCGACGCTCAAGTCAGAGGTGGCGAAACCCGACAGGACTATAAAGATACCAGGCGTTTCCCCCTGGAAGCTCCCTCGTGCGCTCTCCTGTTC
CGACCCTGCCGCTTACCGGATACCTGTCCGCCTTTCTCCCTTCGGGAAGCGTGGCGCTTTCTCATAGCTCACGCTGTAGGTATCTCAGTTCGG
TGTAGGTCGTTCGCTCCAAGCTGGGCTGTGTGCACGAACCCCCCGTTCAGCCCGACCGCTGCGCCTTATCCGGTAACTATCGTCTTGAGTCC
AACCCGGTAAGACACGACTTATCGCCACTGGCAGCAGCCACTGGTAACAGGATTAGCAGAGCGAGGTATGTAGGCGGTGCTACAGAGTTCTT
GAAGTGGTGGCCTAACTACGGCTACACTAGAAGAACAGTATTTGGTATCTGCGCTCTGCTGAAGCCAGTTACCTTCGGAAAAAGAGTTGGTAG
CTCTTGATCCGGCAAACAAACCACCGCTGGTAGCGGTGGTTTTTTTGTTTGCAAGCAGCAGATTACGCGCAGAAAAAAAGGATCTCAAGAAGA
TCCTTTGATCTTTTCTACGGGGTCTGACGCTCAGTGGAACGAAAACTCACGTTAAGGGATTTTGGTCATGAGATTATCAAAAAGGATCTTCACC
TAGATCCTTTTAAATTAAAAATGAAGTTTTAAATCAATCTAAAGTATATATGAGTAAACTTGGTCTGACAGTTACCAATGCTTAATCAGTGAGGCA
CCTATCTCAGCGATCTGTCTATTTCGTTCATCCATAGTTGCCTGACTCCCCGTCGTGTAGATAACTACGATACGGGAGGGCTTACCATCTGGCC
CCAGTGCTGCAATGATACCGCGAGAACCACGCTCACCGGCTCCAGATTTATCAGCAATAAACCAGCCAGCCGGAAGGGCCGAGCGCAGAAG
TGGTCCTGCAACTTTATCCGCCTCCATCCAGTCTATTAATTGTTGCCGGGAAGCTAGAGTAAGTAGTTCGCCAGTTAATAGTTTGCGCAACGTT
GTTGCCATTGCTACAGGCATCGTGGTGTCACGCTCGTCGTTTGGTATGGCTTCATTCAGCTCCGGTTCCCAACGATCAAGGCGAGTTACATGA
TCCCCCATGTTGTGCAAAAAAGCGGTTAGCTCCTTCGGTCCTCCGATCGTTGTCAGAAGTAAGTTGGCCGCAGTGTTATCACTCATGGTTATG
GCAGCACTGCATAATTCTCTTACTGTCATGCCATCCGTAAGATGCTTTTCTGTGACTGGTGAGTACTCAACCAAGTCATTCTGAGAATAGTGTAT

GCGGCGACCGAGTTGCTCTTGCCCGGCGTCAATACGGGATAATACCGCGCCACATAGCAGAACTTTAAAAGTGCTCATCATTGGAAAACGTTC
TTCGGGGCGAAAACTCTCAAGGATCTTACCGCTGTTGAGATCCAGTTCGATGTAACCCACTCGTGCACCCAACTGATCTTCAGCATCTTTTACT
TTCACCAGCGTTTCTGGGTGAGCAAAAACAGGAAGGCAAAATGCCGCAAAAAAGGGAATAAGGGCGACACGGAAATGTTGAATACTCATACTC
TTCCTTTTTCAATATTATTGAAGCATTTATCAGGGTTATTGTCTCATGAGCGGATACATATTTGAATGTATTTAGAAAAATAAACAAATAGGGGTT
CCGCGCACATTTCCCCGAAAAGTGCCAC

Example vector pMA-T with HD-2 inserted and highlighted in yellow:

CTAAATTGTAAGCGTTAATATTTTGTTAAAATTCGCGTTAAATTTTTGTTAAATCAGCTCATTTTTTAACCAATAGGCCGAAATCGGCAAAATCCC
TTATAAATCAAAAGAATAGACCGAGATAGGGTTGAGTGGCCGCTACAGGGCGCTCCCATTCGCCATTCAGGCTGCGCAACTGTTGGGAAGGG
CGTTTCGGTGCGGGCCTCTTCGCTATTACGCCAGCTGGCGAAAGGGGGATGTGCTGCAAGGCGATTAAGTTGGGTAACGCCAGGGTTTTCCC
AGTCACGACGTTGTAAAACGACGGCCAGTGAGCGCGACGTAATACGACTCACTATAGGGCGAATTGGCGGAAGGCCGTCAAGGCCACGTGT
CTTGTCCAGAGCTCGTATCCTGCAGTTAACACCAGAACAGGTCGTTGCAATCGCGTCGCACGACGGGGGAAAGCAAGCCCTGGAAACCGTGC
AAAGGTTGTTGCCGGTTCTATGTCAGGCGCACGGCTTAACTCCAGACCAGGTGGTACTGCAGGATACGGTACCTGGAGCACAAGACTGGCCT
CATGGGCCTTCCGCTCACTGCCCGCTTTCCAGTCGGGAAACCTGTCGTGCCAGCTGCATTAACATGGTCATAGCTGTTTCCTTGCGTATTGGG
CGCTCTCCGCTTCCTCGCTCACTGACTCGCTGCGCTCGGTCGTTCGGGTAAAGCCTGGGGTGCCTAATGAGCAAAAGGCCAGCAAAAGGCCA
GGAACCGTAAAAAGGCCGCGTTGCTGGCGTTTTTCCATAGGCTCCGCCCCCCTGACGAGCATCACAAAAATCGACGCTCAAGTCAGAGGTGG
CGAAACCCGACAGGACTATAAAGATACCAGGCGTTTCCCCCTGGAAGCTCCCTCGTGCGCTCTCCTGTTCCGACCCTGCCGCTTACCGGATA
CCTGTCCGCCTTTCTCCCTTCGGGAAGCGTGGCGCTTTCTCATAGCTCACGCTGTAGGTATCTCAGTTCGGTGTAGGTCGTTCGCTCCAAGCT
GGGCTGTGTGCACGAACCCCCCGTTCAGCCCGACCGCTGCGCCTTATCCGGTAACTATCGTCTTGAGTCCAACCCGGTAAGACACGACTTAT
CGCCACTGGCAGCAGCCACTGGTAACAGGATTAGCAGAGCGAGGTATGTAGGCGGTGCTACAGAGTTCTTGAAGTGGTGGCCTAACTACGG
CTACACTAGAAGAACAGTATTTGGTATCTGCGCTCTGCTGAAGCCAGTTACCTTCGGAAAAAGAGTTGGTAGCTCTTGATCCGGCAAACAAAC
CACCGCTGGTAGCGGTGGTTTTTTTGTTTGCAAGCAGCAGATTACGCGCAGAAAAAAAGGATCTCAAGAAGATCCTTTGATCTTTTCTACGGG
GTCTGACGCTCAGTGGAACGAAAACTCACGTTAAGGGATTTTGGTCATGAGATTATCAAAAAGGATCTTCACCTAGATCCTTTTAAATTAAAAAT
GAAGTTTTAAATCAATCTAAAGTATATATGAGTAAACTTGGTCTGACAGTTACCAATGCTTAATCAGTGAGGCACCTATCTCAGCGATCTGTCTA
TTTCGTTCATCCATAGTTGCCTGACTCCCCGTCGTGTAGATAACTACGATACGGGAGGGCTTACCATCTGGCCCCAGTGCTGCAATGATACCG
CGAGAACCACGCTCACCGGCTCCAGATTTATCAGCAATAAACCAGCCAGCCGGAAGGGCCGAGCGCAGAAGTGGTCCTGCAACTTTATCCGC
CTCCATCCAGTCTATTAATTGTTGCCGGGAAGCTAGAGTAAGTAGTTCGCCAGTTAATAGTTTGCGCAACGTTGTTGCCATTGCTACAGGCATC
GTGGTGTCACGCTCGTCGTTTGGTATGGCTTCATTCAGCTCCGGTTCCCAACGATCAAGGCGAGTTACATGATCCCCCATGTTGTGCAAAAAA
GCGGTTAGCTCCTTCGGTCCTCCGATCGTTGTCAGAAGTAAGTTGGCCGCAGTGTTATCACTCATGGTTATGGCAGCACTGCATAATTCTCTTA
CTGTCATGCCATCCGTAAGATGCTTTTCTGTGACTGGTGAGTACTCAACCAAGTCATTCTGAGAATAGTGTATGCGGCGACCGAGTTGCTCTTG
CCCGGCGTCAATACGGGATAATACCGCGCCACATAGCAGAACTTTAAAAGTGCTCATCATTGGAAAACGTTCTTCGGGGCGAAAACTCTCAAG
GATCTTACCGCTGTTGAGATCCAGTTCGATGTAACCCACTCGTGCACCCAACTGATCTTCAGCATCTTTTACTTTCACCAGCGTTTCTGGGTGA
GCAAAAACAGGAAGGCAAAATGCCGCAAAAAAGGGAATAAGGGCGACACGGAAATGTTGAATACTCATACTCTTCCTTTTTCAATATTATTGAA
GCATTTATCAGGGTTATTGTCTCATGAGCGGATACATATTTGAATGTATTTAGAAAAATAAACAAATAGGGGTTCCGCGCACATTTCCCCGAAAA
GTGCCAC

Supplementary Table S4. 4mers and fully assembled 16mers for a TALEN targeting EGFP (All sequences 5' to 3').

Plus strand target sequence: TCAAGGACGACGGCAACT acaagacccgcgccgaggtgaagttcg AGGGCGACACCCTGGTGA

Upstream TALEN - RVD composition of 16mer – HD NI NI NH NH NI HD NH NI HD NH NH HD NI NI HD; RVDs are shown in bold.

4mer	Length	Sequence - nucleotide sequence and then encoded amino acid sequence
L1-1	481 bp	GAAGAGAGGGGGAGTAACAGCGGTAGAGGCAGTGCACGCCTGGCGCAATGCGCTCACCGGGGCCCCCTTGAACC<br>TGACACCTGACCAAGTCGTGGCAATCGCGTCGCACGACGGGGGAAAGCAAGCCCTGGAAACCGTGCAAAGGTTGT<br>TGCCGGTGCTGTGCCAAGACCACGGCTTAACACCAGAACAGGTCGTTGCAATCGCGTCGAACATTGGGGGAAAGC<br>AAGCCCTGGAAACCGTGCAAAGGTTGTTGCCGGTTCTATGTCAGGCGCACGGCTTAACTCCAGACCAGGTGGTAG<br>CAATCGCGTCGAACATTGGGGGAAAGCAAGCCCTGGAAACCGTGCAAAGGTTGTTGCCGGTACTATGCCAGGCAC<br>ATGGATTAACCCCAGCTCAAGTGGTAGCAATCGCGTCGAATCACGGGGGAAAGCAAGCCCTGGAAACCGTGCAAA<br>GGTTGTTGCCGGTCTTGTGCCAAGATCATGGG<br><br>KRGGVTAVEAVHAWRNALTGAPLNLTPDQVVAI AS**HD**GGKQALETVQRLLPVLCQDHGLTPEQVVAI AS**NI** GGKQALET<br>VQRLLPVLCQAHGLTPDQVVAI AS**NI** GGKQALETVQRLLPVLCQAHGLTPAQVVAI AS**NH**GGKQALETVQRLLPVLCQDH<br>G
L1-2	429 bp	GTCTTGTGCCAAGATCATGGGCTTACTCCCGATCAGGTGGTCGCAATCGCGTCGAATCACGGGGGAAAGCAAGCC<br>CTGGAAACCGTGCAAAGGTTGTTGCCGGTACTTTGCCAGGACCATGGATTAACTCCTGAGCAAGTGGTCGCAATCG<br>CGTCGAACATTGGGGGAAAGCAAGCCCTGGAAACCGTGCAAAGGTTGTTGCCGGTGTTGTGCCAAGCACATGGCT<br>TAACACCGGATCAAGTCGTGGCAATCGCGTCGCACGACGGGGGAAAGCAAGCCCTGGAAACCGTGCAAAGGTTGT<br>TGCCGGTCCTGTGTCAGGCGCATGGATTAACGCCCGCACAGGTTGTTGCAATCGCGTCGAATCACGGGGGAAAGC<br>AAGCCCTGGAAACCGTGCAAAGGTTGTTGCCGGTTCTATGCCAGGATCACGGG<br><br>VLCQDHGLTPDQVVAI AS**NH**GGKQALETVQRLLPVLCQDHGLTPEQVVAI AS**NI** GGKQALETVQRLLPVLCQAHGLTPDQ

		VVAI AS**HD**GGKQALETVQRLLPVLCQAHGLTPAQVVAI AS**NH**GGKQALETVQRLLPVLCQDHG
L1-3	429 bp	GTTCTATGCCAGGATCACGGGCTTACCCCGGACCAAGTAGTAGCAATCGCGTCGAACATTGGGGGAAAGCAAGCC<br>CTGGAAACCGTGCAAAGGTTGTTGCCGGTTTTATGTCAAGACCACGGTTTAACCCCCGAACAGGTAGTGGCAATCG<br>CGTCGCACGACGGGGGAAAGCAAGCCCTGGAAACCGTGCAAAGGTTGTTGCCGGTCCTCTGTCAGGCTCACGGAT<br>TAACGCCCGACCAGGTTGTTGCAATCGCGTCGAATCACGGGGGAAAGCAAGCCCTGGAAACCGTGCAAAGGTTGT<br>TGCCGGTGTTGTGCCAAGCGCACGGCTTAACGCCGGCCCAAGTAGTCGCAATCGCGTCGAATCACGGGGGAAAG<br>CAAGCCCTGGAAACCGTGCAAAGGTTGTTGCCGGTATTATGTCAAGATCATGGG<br><br>VLCQDHGLTPDQVVAI AS**NI** GGKQALETVQRLLPVLCQDHGLTPEQVVAI AS**HD**GGKQALETVQRLLPVLCQAHGLTPDQ<br>VVAI AS**NH**GGKQALETVQRLLPVLCQAHGLTPAQVVAI AS**NH**GGKQALETVQRLLPVLCQDHG
L1-4	429 bp	GTATTATGTCAAGATCATGGGCTCACGCCAGATCAGGTTGTTGCAATCGCGTCGCACGACGGGGGAAAGCAAGCC<br>CTGGAAACCGTGCAAAGGTTGTTGCCGGTCTTGTGTCAGGACCATGGCTTAACGCCGGAGCAAGTTGTAGCAATC<br>GCGTCGAACATTGGGGGAAAGCAAGCCCTGGAAACCGTGCAAAGGTTGTTGCCGGTATTATGCCAAGCCCATGGT<br>TTAACCCCTGATCAAGTAGTCGCAATCGCGTCGAACATTGGGGGAAAGCAAGCCCTGGAAACCGTGCAAAGGTTGT<br>TGCCGGTTCTCTGTCAAGCCCACGGTTTAACTCCGGCGCAGGTCGTGGCAATCGCGTCGCACGACGGGGGAAAG<br>CAAGCCCTGGAAACCGTGCAAAGGTTGTTGCCGGTGCTCTGTCAGGACCACGGG<br><br>VLCQDHGLTPDQVVAI AS**HD**GGKQALETVQRLLPVLCQDHGLTPEQVVAI AS**NI** GGKQALETVQRLLPVLCQAHGLTPDQ<br>VVAI AS**NI** GGKQALETVQRLLPVLCQAHGLTPAQVVAI AS**HD**GGKQALETVQRLLPVLCQDHG
16mer	1632 bp	CTGACACCTGACCAAGTCGTGGCAATCGCGTCGCACGACGGGGGAAAGCAAGCCCTGGAAACCGTGCAAAGGTTG<br>TTGCCGGTGCTGTGCCAAGACCACGGCTTAACACCAGAACAGGTCGTTGCAATCGCGTCGAACATTGGGGGAAAG

		CAAGCCCTGGAAACCGTGCAAAGGTTGTTGCCGGTTCTATGTCAGGCGCACGGCTTAACTCCAGACCAGGTGGTA<br>GCAATCGCGTCGAACATTGGGGGAAAGCAAGCCCTGGAAACCGTGCAAAGGTTGTTGCCGGTACTATGCCAGGCA<br>CATGGATTAACCCCAGCTCAAGTGGTAGCAATCGCGTCGAATCACGGGGGAAAGCAAGCCCTGGAAACCGTGCAA<br>AGGTTGTTGCCGGTCTTGTGCCAAGATCATGGGCTTACTCCCGATCAGGTGGTCGCAATCGCGTCGAATCACGGG<br>GGAAAGCAAGCCCTGGAAACCGTGCAAAGGTTGTTGCCGGTACTTTGCCAGGACCATGGATTAACTCCTGAGCAA<br>GTGGTCGCAATCGCGTCGAACATTGGGGGAAAGCAAGCCCTGGAAACCGTGCAAAGGTTGTTGCCGGTGTTGTGC<br>CAAGCACATGGCTTAACACCGGATCAAGTCGTGGCAATCGCGTCGCACGACGGGGGAAAGCAAGCCCTGGAAACC<br>GTGCAAAGGTTGTTGCCGGTCTTTTGTCAGGCGCATGGATTAACGCCCGCACAGGTTGTTGCAATCGCGTCGAATC<br>ACGGGGGAAAGCAAGCCCTGGAAACCGTGCAAAGGTTGTTGCCGGTTCTATGCCAGGATCACGGGCTTACCCCGG<br>ACCAAGTAGTAGCAATCGCGTCGAACATTGGGGGAAAGCAAGCCCTGGAAACCGTGCAAAGGTTGTTGCCGGTTC<br>TATGTCAAGACCACGGTTTAACCCCCGAACAGGTAGTGGCAATCGCGTCGCACGACGGGGGAAAGCAAGCCCTGG<br>AAACCGTGCAAAGGTTGTTGCCGGTCCTCTGTCAGGCTCACGGATTAACGCCCGACCAGGTTGTTGCAATCGCGTC<br>GAATCACGGGGGAAAGCAAGCCCTGGAAACCGTGCAAAGGTTGTTGCCGGTGTTGTGCCAAGCGCACGGCTTAAC<br>GCCGGCCCAAGTAGTCGCAATCGCGTCGAATCACGGGGGAAAGCAAGCCCTGGAAACCGTGCAAAGGTTGTTGCC<br>GGTATTATGTCAAGATCATGGGCTCACGCCAGATCAGGTTGTTGCAATCGCGTCGCACGACGGGGGAAAGCAAGC<br>CCTGGAAACCGTGCAAAGGTTGTTGCCGGTCTTGTGTCAGGACCATGGCTTAACGCCGGAGCAAGTTGTAGCAATC<br>GCGTCGAACATTGGGGGAAAGCAAGCCCTGGAAACCGTGCAAAGGTTGTTGCCGGTATTATGCCAAGCCCATGGT<br>TTAACCCCTGATCAAGTAGTCGCAATCGCGTCGAACATTGGGGGAAAGCAAGCCCTGGAAACCGTGCAAAGGTTGT<br>TGCCGGTTCTCTGTCAAGCCCACGGTTTAACTCCGGCGCAGGTCGTGGCAATCGCGTCGCACGACGGGGGAAAG<br>CAAGCCCTGGAAACCGTGCAAAGGTTGTTGCCGGTGCTCTGTCAGGACCACGGG<br><br>LTPDQVVAIASHDGGKQALETVQRLLPVLCQDHGLTPEQVVAIASNIGGKQALETVQRLLPVLCQAHGLTPDQVVAIASNI<br>GGKQALETVQRLLPVLCQAHGLTPAQVVAIASNHGGKQALETVQRLLPVLCQDHGLTPDQVVAIASNHGGKQALETVQR<br>LLPVLCQDHGLTPEQVVAIASNIGGKQALETVQRLLPVLCQAHGLTPDQVVAIASHDGGKQALETVQRLLPVFCQAHGLT<br>PAQVVAIASNHGGKQALETVQRLLPVLCQDHGLTPDQVVAIASNIGGKQALETVQRLLPVLCQDHGLTPEQVVAIASHDG<br>GKQALETVQRLLPVLCQAHGLTPDQVVAIASNHGGKQALETVQRLLPVLCQAHGLTPAQVVAIASNHGGKQALETVQRL<br>LPVLCQDHGLTPDQVVAIASHDGGKQALETVQRLLPVLCQDHGLTPEQVVAIASNIGGKQALETVQRLLPVLCQAHGLTP

		DQVVAI ASNI GGKQALETVQRLLPVLCQAHGLTPAQVVAI ASHDGGKQALETVQRLLPVLCQDHG

Downstream TALEN - RVD composition of 16mer - HD NI HD HD NI NH NH NH NG NH NG HD NH HD HD HD; RVDs are shown in bold.

4mer	Length	Sequence - nucleotide sequence and then encoded amino acid sequence
R1-1	481 bp	GAAGAGAGGGGGAGTAACAGCGGTAGAGGCAGTGCACGCCTGGCGCAATGCGCTCACCGGGGCCCCTTGAACCTGACACCTGACCAAGTCGTGGCAATCGCGTCGCACGACGGGGGAAAGCAAGCCCTGGAAACCGTGCAAAGGTTGTTGCCGGTGCTGTGCCAAGACCACGGCTTAACACCAGAACAGGTCGTTGCAATCGCGTCGAACATTGGGGGAAAGCAAGCCCTGGAAACCGTGCAAAGGTTGTTGCCGGTTCTATGTCAGGCGCACGGCTTAACTCCAGACCAGGTGGTAGCAATCGCGTCGCACGACGGGGGAAAGCAAGCCCTGGAAACCGTGCAAAGGTTGTTGCCGGTACTATGCCAGGCACATGGATTAACCCCAGCTCAAGTGGTAGCAATCGCGTCGCACGACGGGGGAAAGCAAGCCCTGGAAACCGTGCAAAGGTTGTTGCCGGTCTTGTGCCAAGATCATGGG<br><br>KRGGVTAVEAVHAWRNALTGAPLNLTPDQVVAI AS**HD**GGKQALETVQRLLPVLCQDHGLTPEQVVAI AS**NI** GGKQALETVQRLLPVLCQAHGLTPDQVVAI AS**HD**GGKQALETVQRLLPVLCQAHGLTPAQVVAI AS**HD**GGKQALETVQRLLPVLCQDHG
R1-2	429 bp	GTCTTGTGCCAAGATCATGGGCTTACTCCCGATCAGGTGGTCGCAATCGCGTCGAACATTGGGGGAAAGCAAGCCCTGGAAACCGTGCAAAGGTTGTTGCCGGTACTTTGCCAGGACCATGGATTAACTCCTGAGCAAGTGGTCGCAATCGCGTCGAATCACGGGGGAAAGCAAGCCCTGGAAACCGTGCAAAGGTTGTTGCCGGTGTTGTGCCAAGCACATGGCTTAACACCGGATCAAGTCGTGGCAATCGCGTCGAATCACGGGGGAAAGCAAGCCCTGGAAACCGTGCAAAGGTTGTTGCCGGTCCTGTGTCAGGCGCATGGATTAACGCCCGCACAGGTTGTTGCAATCGCGTCGAATCACGGGGGAAAGCAAGCCCTGGAAACCGTGCAAAGGTTGTTGCCGGTTCTATGCCAGGATCACGGG

		VLCQDHGLTPDQVVAIAS**NI**GGKQALETVQRLLPVLCQDHGLTPEQVVAIAS**NH**GGKQALETVQRLLPVLCQAHGLTPDQ<br>VVAIAS**NH**GGKQALETVQRLLPVLCQAHGLTPAQVVAIAS**NH**GGKQALETVQRLLPVLCQDHG
R1-3	429 bp	GTTCTATGCCAGGATCACGGGCTTACCCCGGACCAAGTAGTAGCAATCGCGTCGAATGGAGGGGGAAAGCAAGCC<br>CTGGAAACCGTGCAAAGGTTGTTGCCGGTTTTATGTCAAGACCACGGTTTAACCCCCGAACAGGTAGTGGCAATCG<br>CGTCGAATCACGGGGGAAAGCAAGCCCTGGAAACCGTGCAAAGGTTGTTGCCGGTCCTCTGTCAGGCTCACGGAT<br>TAACGCCCGACCAGGTTGTTGCAATCGCGTCGAATGGAGGGGGAAAGCAAGCCCTGGAAACCGTGCAAAGGTTGT<br>TGCCGGTGTTGTGCCAAGCGCACGGCTTAACGCCGGCCCAAGTAGTCGCAATCGCGTCGCACGACGGGGGAAAG<br>CAAGCCCTGGAAACCGTGCAAAGGTTGTTGCCGGTATTATGTCAAGATCATGGG<br><br>VLCQDHGLTPDQVVAIAS**NG**GGKQALETVQRLLPVLCQDHGLTPEQVVAIAS**NH**GGKQALETVQRLLPVLCQAHGLTPD<br>QVVAIAS**NG**GGKQALETVQRLLPVLCQAHGLTPAQVVAIAS**HD**GGKQALETVQRLLPVLCQDHG
R1-4	429 bp	GTATTATGTCAAGATCATGGGCTCACGCCAGATCAGGTTGTTGCAATCGCGTCGAATCACGGGGGAAAGCAAGCCC<br>TGGAAACCGTGCAAAGGTTGTTGCCGGTCTTGTGTCAGGACCATGGCTTAACGCCGGAGCAAGTTGTAGCAATCG<br>CGTCGCACGACGGGGGAAAGCAAGCCCTGGAAACCGTGCAAAGGTTGTTGCCGGTATTATGCCAAGCCCATGGTT<br>TAACCCCTGATCAAGTAGTCGCAATCGCGTCGCACGACGGGGGAAAGCAAGCCCTGGAAACCGTGCAAAGGTTGT<br>TGCCGGTTCTCTGTCAAGCCCACGGTTTAACTCCGGCGCAGGTCGTGGCAATCGCGTCGCACGACGGGGGAAAG<br>CAAGCCCTGGAAACCGTGCAAAGGTTGTTGCCGGTGCTCTGTCAGGACCACGGG<br><br>VLCQDHGLTPDQVVAIAS**NH**GGKQALETVQRLLPVLCQDHGLTPEQVVAIAS**HD**GGKQALETVQRLLPVLCQAHGLTPD<br>QVVAIAS**HD**GGKQALETVQRLLPVLCQAHGLTPAQVVAIAS**HD**GGKQALETVQRLLPVLCQDHG
16mer	1632	CTGACACCTGACCAAGTCGTGGCAATCGCGTCGCACGACGGGGGAAAGCAAGCCCTGGAAACCGTGCAAAGGTTG

	bp	TTGCCGGTGCTGTGCCAAGACCACGGCTTAACACCAGAACAGGTCGTTGCAATCGCGTCGAACATTGGGGGAAAG<br>CAAGCCCTGGAAACCGTGCAAAGGTTGTTGCCGGTTCTATGTCAGGCGCACGGCTTAACTCCAGACCAGGTGGTA<br>GCAATCGCGTCGCACGACGGGGGAAAGCAAGCCCTGGAAACCGTGCAAAGGTTGTTGCCGGTACTATGCCAGGC<br>ACATGGATTAACCCCAGCTCAAGTGGTAGCAATCGCGTCGCACGACGGGGGAAAGCAAGCCCTGGAAACCGTGCA<br>AAGGTTGTTGCCGGTCTTGTGCCAAGATCATGGGCTTACTCCCGATCAGGTGGTCGCAATCGCGTCGAACATTGGG<br>GGAAAGCAAGCCCTGGAAACCGTGCAAAGGTTGTTGCCGGTACTTTGCCAGGACCATGGATTAACTCCTGAGCAA<br>GTGGTCGCAATCGCGTCGAATCACGGGGGAAAGCAAGCCCTGGAAACCGTGCAAAGGTTGTTGCCGGTGTTGTGC<br>CAAGCACATGGCTTAACACCGGATCAAGTCGTGGCAATCGCGTCGAATCACGGGGGAAAGCAAGCCCTGGAAACC<br>GTGCAAAGGTTGTTGCCGGTCTTTTGTCAGGCGCATGGATTAACGCCCGCACAGGTTGTTGCAATCGCGTCGAATC<br>ACGGGGGAAAGCAAGCCCTGGAAACCGTGCAAAGGTTGTTGCCGGTTCTATGCCAGGATCACGGGCTTACCCCGG<br>ACCAAGTAGTAGCAATCGCGTCGAATGGAGGGGGAAAGCAAGCCCTGGAAACCGTGCAAAGGTTGTTGCCGGTTC<br>TATGTCAAGACCACGGTTTAACCCCCGAACAGGTAGTGGCAATCGCGTCGAATCACGGGGGAAAGCAAGCCCTGG<br>AAACCGTGCAAAGGTTGTTGCCGGTCCTCTGTCAGGCTCACGGATTAACGCCCGACCAGGTTGTTGCAATCGCGTC<br>GAATGGAGGGGGAAAGCAAGCCCTGGAAACCGTGCAAAGGTTGTTGCCGGTGTTGTGCCAAGCGCACGGCTTAAC<br>GCCGGCCCAAGTAGTCGCAATCGCGTCGCACGACGGGGGAAAGCAAGCCCTGGAAACCGTGCAAAGGTTGTTGC<br>CGGTATTATGTCAAGATCATGGGCTCACGCCAGATCAGGTTGTTGCAATCGCGTCGAATCACGGGGGAAAGCAAG<br>CCCTGGAAACCGTGCAAAGGTTGTTGCCGGTCTTGTGTCAGGACCATGGCTTAACGCCGGAGCAAGTTGTAGCAAT<br>CGCGTCGCACGACGGGGGAAAGCAAGCCCTGGAAACCGTGCAAAGGTTGTTGCCGGTATTATGCCAAGCCCATGG<br>TTTAACCCCTGATCAAGTAGTCGCAATCGCGTCGCACGACGGGGGAAAGCAAGCCCTGGAAACCGTGCAAAGGTT<br>GTTGCCGGTTCTCTGTCAAGCCCACGGTTTAACTCCGGCGCAGGTCGTGGCAATCGCGTCGCACGACGGGGGAAA<br>GCAAGCCCTGGAAACCGTGCAAAGGTTGTTGCCGGTGCTCTGTCAGGACCACGGG<br><br>LTPDQVVAIASHDGGKQALETVQRLLPVLCQDHGLTPEQVVAIASNIGGKQALETVQRLLPVLCQAHGLTPDQVVAIASH<br>DGGKQALETVQRLLPVLCQAHGLTPAQVVAIASHDGGKQALETVQRLLPVLCQDHGLTPDQVVAIASNIGGKQALETVQ<br>RLLPVLCQDHGLTPEQVVAIASNHGGKQALETVQRLLPVLCQAHGLTPDQVVAIASNHGGKQALETVQRLLPVFCQAHG<br>LTPAQVVAIASNHGGKQALETVQRLLPVLCQDHGLTPDQVVAIASNGGGKQALETVQRLLPVLCQDHGLTPEQVVAIASN<br>HGGKQALETVQRLLPVLCQAHGLTPDQVVAIASNGGGKQALETVQRLLPVLCQAHGLTPAQVVAIASHDGGKQALETVQ

		RLLPVLCQDHGLTPDQVVAIASNHGGKQALETVQRLLPVLCQDHGLTPEQVVAIASHDGGKQALETVQRLLPVLCQAHG LTPDQVVAIASHDGGKQALETVQRLLPVLCQAHGLTPAQVVAIASHDGGKQALETVQRLLPVLCQDHG

Supplementary Table S5. Target sequences of hSox2 TALE TFs.

Target gene	System	gRNA equivalent	TALE TF ID	Target sequence
hSox2	TALE TF	gRNA 3	TALE TF 3	TCCCTGACAGCCCCCGTC
		gRNA 4	TALE TF 4	TTTCATGCAAAACCCGGC
		gRNA 5	TALE TF 5	TCCCCCGGCCTCCCCCGC
		gRNA 6	TALE TF 6	TCCTCCCCCTCCTCGCCT
		gRNA 7	TALE TF 7	TGCTGCGAGAGGGGATAC

Target gene	System	gRNA name	Target sequence	Oligo 1	Oligo 2
hSox2	dCas9 TF	gRNA 3	gCATGTGACGGGGGCTGTCAGGG	accGCATGTGACGGGGGCTGTCA	aaacTGACAGCCCCCGTCACATG
		gRNA 4	GCTGCCGGGTTTTGCATGAAAGG	accGCTGCCGGGTTTTGCATGAA	aaacTTCATGCAAAACCCGGCAG
		gRNA 5	GCCGGCCGCGCGGGGGAGGCCGG	accGCCGGCCGCGCGGGGGAGGC	aaacGCCTCCCCCGCGCGGCCGG
		gRNA 6	GGCAGGCGAGGAGGGGGAGGAGG	accGGCAGGCGAGGAGGGGGAGG	aaacCCTCCCCCTCCTCGCCTGC
		gRNA 7	GTATCCCCTCTCGCAGCAACAGG	accGTATCCCCTCTCGCAGCAAC	aaacGTTGCTGCGAGAGGGGATA

Supplementary Table S6. Functional domain sequences for TALE toolbox.

Domain	Sequence (BsmBI recognition site bold/underlined.)
VP160_2A-mCherry	GTATCCCGAGGCCAGCGGTTTCCGGAgcagtg**CGTCTC**gCagtCTCGCGCCGACGCGCTGGACGATTTCGATCTCGACATGCT<br>GGGTTCTGATGCCCTCGATGACTTTGACCTGGATATGTTGGGAAGCGACGCATTGGATGACTTTGATCTGGACATGCTCGGCT<br>CCGATGCTCTGGACGATTTCGATCTCGATATGTTAgggtcagacgcactggatgatttcgaccttgatatgttgGGAAGCGAT<br>GCCCTTGATGATTTCGACCTGGACATGCTCggcagcgacgccctggacgatttcgatctggacatgctgGGGTCCGATGCCTT<br>GGATGATTTTGACTTGGATATGCTGgggagtgatgccctggacgactttgacctggacatgctgGGCTCCGATGCGCTCGATG<br>ACTTCGATTTGGAtATGTTGATTAACTCTAGAGGCAGTGGAgccactaacttctccctgttgaaacaagcaggggatgtcgaa<br>gagaatcccgggccaatggtgagcaagggcgaggaggataacatggccatcatcaaggagttcatgcgcttcaaggtgcacat<br>ggagggctccgtgaacggccacgagttcgagatcgagggcgagggcgagggccgcccctacgagggcacccagaccgccaagc<br>tgaaggtgaccaagggtggcccctgcccttcgcctgggacatcctgtcccctcagttcatgtacggctccaaggcctacgtg<br>aagcaccccgccgacatccccgactacttgaagctgtccttccccgagggcttcaagtgggagcgcgtgatgaacttcgagga<br>cggcggcgtggtgaccgtgacccaggactcctccctgcaggacggcgagttcatctacaaggtgaagctgcgcggcaccaact<br>tcccctccgacggccccgtaatgcagaagaagaccatgggctgggaggcctcctccgagcggatgtaccccgaggacggcgcc<br>ctgaagggcgagatcaagcagaggctgaagctgaaggacggcggccactacgacgctgaggtcaagaccacctacaaggccaa<br>gaagcccgtgcagctgcccggcgcctacaacgtcaacatcaagttggacatcacctcccacaacgaggactacaccatcgtgg<br>aacagtacgaacgcgccgagggccgccactccaccggcggcatggacgagctgtacaagtaacgcccgccccacgacccgcag<br>cgcccgaccgaaaggagcgcacgaccccatgcatcgatgatctagagctc**GAGACG**aagctcctcgggGGATAC
KRAB	TACGGAT**CGTCTC**GCAGTggATGGACGCGAAATCACTTACGGCATGGTCGAGAACACTGGTTACGTTCAAGGACGTGTTTGTG<br>GACTTTACACGTGAGGAGTGGAAATTGCTGGATACTGCGCAACAAATTGTGTATCGAAATGTCATGCTTGAGAATTACAAGAA<br>CCTCGTCAGTCTCGGATACCAGTTGACGAAACCGGATGTGATCCTTAGGCTCGAAAAGGGGGAAGAACCTTGGCTGGTATAAA<br>GCTCG**AGACG**TACGG
p300core	GTATCCCGAGGCCAGCGGTTTCCGGAgcagtg**CGTCTC**gCagtCTATTTTCAAACCAGAAGAACTACGACAGGCACTGATGCC<br>AACTTTGGAGGCACTTTACCGTCAGGATCCAGAATCCCTTCCCTTTCGTCAACCTGTGGACCCTCAGCTTTTAGGAATCCCTG<br>ATTACTTTGATATTGTGAAGAGCCCCATGGATCTTTCTACCATTAAGAGGAAGTTAGACACTGGACAGTATCAGGAGCCCTGG<br>CAGTATGTCGATGATATTTGGCTTATGTTCAATAATGCCTGGTTATATAACCGGAAAACATCACGGGTATACAAATACTGCTC<br>CAAGCTCTCTGAGGTCTTTGAACAAGAAATTGACCCAGTGATGCAAAGCCTTGGATACTGTTGTGGCAGAAAGTTGGAGTTCT<br>CTCCACAGACACTGTGTTGCTACGGCAAACAGTTGTGCACAATACCTCGTGATGCCACTTATTACAGTTACCAGAACAGGTAT<br>CATTTCTGTGAGAAGTGTTTCAATGAGATCCAAGGGGAGAGCGTTTCTTTGGGGGATGACCCTTCCCAGCCTCAAACTACAAT<br>AAATAAAGAACAATTTTCCAAGAGAAAAAATGACACACTGGATCCTGAACTGTTTGTTGAATGTACAGAGTGCGGAAGAAAGA<br>TGCATCAGATCTGTGTCCTTCACCATGAGATCATCTGGCCTGCTGGATTCGTCTGTGATGGCTGTTTAAAGAAAAGTGCACGA<br>ACTAGGAAAGAAAATAAGTTTTCTGCTAAAAGGTTGCCATCTACCAGACTTGGCACCTTTCTAGAGAATCGTGTGAATGACTT<br>TCTGAGGCGACAGAATCACCCTGAGTCAGGAGAGGTCACTGTTAGAGTAGTTCATGCTTCTGACAAAACCGTGGAAGTAAAAC<br>CAGGCATGAAAGCAAGGTTTGTGGACAGTGGAGAGATGGCAGAATCCTTTCCATACCGAACCAAAGCCCTCTTTGCCTTTGAA<br>GAAATTGATGGTGTTGACCTGTGCTTCTTTGGCATGCATGTTCAAGAGTATGGCTCTGACTGCCCTCCACCCAACCAGAGGAG<br>AGTATACATATCTTACCTCGATAGTGTTCATTTCTTCCGTCCTAAATGCTTGAGGACTGCAGTCTATCATGAAATCCTAATTG<br>GATATTTAGAATATGTCAAGAAATTAGGTTACACAACAGGGCATATTTGGGCATGTCCACCAAGTGAGGGAGATGATTATATC<br>TTCCATTGCCATCCTCCTGACCAGAAGATACCCAAGCCCAAGCGACTGCAGGAATGGTACAAAAAAATGCTTGACAAGGCTGT<br>ATCAGAGCGTATTGTCCATGACTACAAGGATATTTTTAAACAAGCTACTGAAGATAGATTAACAAGTGCAAAGGAATTGCCTT<br>ATTTCGAGGGTGATTTCTGGCCCAATGTTCTGGAAGAAAGCATTAAGGAACTGGAACAGGAGGAAGAAGAGAGAAAACGAGAG<br>GAAAACACCAGCAATGAAAGCACAGATGTGACCAAGGGAGACAGCAAAAATGCTAAAAAGAAGAATAATAAGAAAACCAGCAA<br>AAATAAGAGCAGCCTGAGTAGGGGCAACAAGAAGAAACCCGGGATGCCCAATGTATCTAACGACCTCTCACAGAAACTATATG<br>CCACCATGGAGAAGCATAAAGAGGTCTTCTTTGTGATCCGCCTCATTGCTGGCCCTGCTGCCAACTCCCTGCCTCCCATTGTT<br>GATCCTGATCCTCTCATCCCCTGCGATCTGATGGATGGTCGGGATGCGTTTCTCACGCTGGCAAGGGACAAGCACCTGGAGTT<br>CTCTTCACTCCGAAGAGCCCAGTGGTCCACCATGTGCATGCTGGTGGAGCTGCACACGCAGAGCCAGGACTACCCGTACGACG<br>TTCCGGACTACGCTTCTTGAagagctc**GAGACG**aagctcctcgggGGATAC
